# Supplementary material for: Longitudinal Resilience and Risk Factors in Pediatric Postoperative Pain (LORRIS): Protocol for a Prospective Longitudinal Swiss University Children’s Hospitals-Based Study
Source: BMJ Open. 2024 Mar 28;14(3):e080174. doi: 10.1136/bmjopen-2023-080174 (PMC10982766; doi:10.1136/bmjopen-2023-080174)
Supplement: Supplementary data [file bmjopen-2023-080174supp001.pdf]

Supplement: Overview of reliability, validity, benefits, and limitations of questionnaires used in the study.

**Domain**		**Measure**	**Reliability**	**Validity**	**Benefits**	**Limitations**
**Experience-sampling method**	Positive and negative emotions*	Items from the Positive and Negative Affect Schedule (1)	Cronbach's Alpha high, .86 for PA and .87 for NA scale (1).	Excellent factorial validity (convergent correlation high: .89 to .95; discriminant correlation low: -.02 to -.18); items good markers of corresponding factors (1).	External validity demonstrated by correlation with other scales. Stability over 2-month time period (1).	Heterogeneity the factorial structure of the PANAS scores (2).
	Pain severity	Numeric Rating Scale (NRS), 0-10				
**Psychosocial factors child**						
	Pain severity (intensity, distress, inter-ference)*	Numeric Rating Scale (NRS), 0-10			Individual scores of pain severity can be converted into the ICD-11 categories of none (0), mild (1), moderate (2) or severe (3) and merged into one extension code endorsed by the WHO (3,4).	More field testing is needed to further validate NRS scales in pediatric populations (5).
	Pre-operative screening	Pediatric Pain Screening Tool (6,7)	Overall PPST: adequate to excellent discrimination; psychosocial subscale AUC	Sensitivity and specificity for the PPST ranged from adequate to excellent, with regard to significant disability (78%, 68%) and high	Defines risk groups to inform efficient treatment decision-making; valid screening tool that allows early	Generalizability of results needs further studies in surgeries with different pathology and requires further

		adequate to acceptable (6). Total score (ICC = 0.75) and psychosocial subscale (ICC = 0.70) acceptable 2-week test-retest reliability (6).	emotional distress (81%, 63%) (6).	identification of patients presenting with impairing pain-related comorbidities (6).	stratification by race and surgical/pain characteristics, and interventions (7). German translation not yet validated.
Functional Disability	Functional Disability Index (FDI; (8))	Good internal reliability of the total FDI in the German cohort (Cronbach's α = .9) similar to the original US version (8).	Good construct validity with significant correlations between the FDI and different variables (e.g. Childhood Health Assessment Questionnaire [total score], pain-related interference in everyday life, affective distress) (8).	Tested in a large cohort of German children and adolescents. Data confirm that the German version of the FDI retains the characteristics of the original FDI and is a reliable and valid instrument (8).	Majority of females subjects; inclusion of patients from a single specialized center restrict the generalizability of the results (8).
Fear of Pain*	Fear of Pain Questionnaire (9)	Good internal consistency of the total scale Cronbach's α = 0.87. Internal consistency also good for both subscales (Fear subscale: α = 0.89; avoidance subscale: α = 0.76) (9)	The two-factor structure of the GFOPQ-C shows that fear of pain includes two key dimensions: Fear of Pain and Avoidance of Activities (9).	Accurate translation of the English version conducted on the basis of the forward–backward translation. Instrument applied in a mixed pain sample (9).	To evaluate the degree of increased fear of pain in pain samples, it would be beneficial a comparison to healthy controls (9)

Pain cata-strophizing *	Pain Catastrophizin g Scale – 3 item version (10)	Chronbach's Alpha high α = .84 and .79 for PCS-C state (10)	Significant relationships with child pain-related outcomes (ie, child pain intensity and child state anxiety), no issue with multicollinearity (10)	Short (3-item scale) can be efficiently used (= high correlation with 6-item scale). Large, diverse sample of children (10)	Due to small number of items per factor, additional research needed to support the 1-factor model. (10)
Attachment parent–child relationship *	Security Scale (11)	Cronbach's α = .74 for both mothers and fathers of sixth graders; and α = .75 and α = .52 for mothers and fathers of third graders respectively (11)	Moderate stability and meaningful associations with other attachment measures, caregiver sensitivity, and indexes of child's adjustment (1).	One of the most widely used measures of attachment in middle childhood and early adolescence. Some associations of the scale with child's outcomes hold regardless of the child's sex and age (12).	Many cross-sectional rather than longitudinal studies (12).
Symptoms of anxiety and depression*	Revised Child Anxiety and Depression Scale-short version (13).	15-item Anxiety Total scale evidenced significant correspondence with anxiety diagnostic groups,10-item Depression Total scale associated with acceptable reliability (Cronbach's α =	Based on a cross-sample validation strategy, values fell in the "acceptable" classification category (AUC=.74, SE=.03) (13).	Potentially useful for repeated measurement in clinical settings as well as wide-scale screenings. Assesses both anxiety and depressive symptoms (13).	Additional test development efforts needed to ensure that the shortened RCADS meets the clinical demands of reduced test length, while maintaining adequate reliability of its broad scale scores (13).

		.80 and .79, respectively) (13).			
Sleep*	Pittsburgh Sleep Quality Index (PSQI; (14))	High internal consistency (Cronbach's α = 0.83) (14)	Sensitive and specific measure: Global PSQI score > 5, sensitivity of 89.6% and specificity of 86.5% (kappa = 0.75, p < 0.001) (14)	Provides a standardized, quantitative measure of sleep quality that quickly identifies good and poor sleepers (14)	Adult population sample (14)
Sensory processing sensitivity	Highly Sensitive Child Scale (15)	HSC-12-items: acceptable internal consistency with Cronbach's α = .71 and .74. The subscales showed lower internal consistency with α = .73/.69 (15).	Divergent validity was established (15).	12-item HSC scale is a psychometrically robust measure that performs well in both children and adolescents. Studies with large sample, replication of results, previous use in samples with chronic pain (15,16).	All data are based on self-report. Most data were provided by children and adolescents residing in the United Kingdom (15).
Emotion regulation*	Emotion Regulation Questionnaire – Child and Adolescent version (17,18)	Sound internal consistency: cognitive reappraisal subscale Cronbach's α = .82, expressive suppression subscale α = .79 (17).	Good factor structure, convergent validity supported (17).	Tested on participants aged between 10 and 18 years (17), previously used in samples with chronic pain (16,19).	Measures only two emotion regulation strategies, cognitive reappraisal and expressive suppression (20).
Quality of life	Pediatric Quality of Life Inventory (21)	High internal consistency (Cronbach's α =	Across scales, item-level internal consistency supported content	Cronbach α good in various samples, including	Issue of detecting small changes in quality of life only

			0.83 for self-report and α = 0.86 for parent-report). α for self-report ranged from .70 to .89 (19).	validity. Item-level discriminant validity tests showed more variability (21)	adolescent samples with chronic pain (16).	partially addressed, more longitudinal data could help to better understand this aspect (21).
	Social support*	Social Support Questionnaire for Children (22)	High internal consistency. Cronbach's α for total scale and five subscales ranging from .89 to .97 (23).	Good factorial and construct validity.Convergent validity was partially supported (23).	The SSQC identifies important sources of social support in varying samples (e.g. for particular ethnic populations the extended family and community member (23).	Substantially longer than a widely used measure of social support in children (23).
	Posttraumatic stress symptoms	Child and Adolescent Trauma Screening (CATS) (24).	Good to excellent reliability with Cronbach's α ranging between .88 and .94 for all three language samples (US, Germany, Norway) (25).	Convergent discriminant validity: medium to strong correlations with measures of depression (r =.62–.82) and anxiety (r =.40–.77). Low to medium correlations with externalizing symptoms (r =−.15–.43) (25).	Different countries involved in the validation (USA, Germany, and Norway) (25).	Pending external validation with a DSM-5 based semi-structured clinical interview (25).
**Psychosocial factors parents**						
	Parents' own pain history*	2 questions: "(1) Have you ever and (2) are you currently				

	living with persistent or recurrent pain for at least 3 months?"				
Postoperative pain measure (child's pain)	Postoperative Pain Measure for Parents (26)	Cronbach's α = .88 and .87 on postoperative days 1 and 2, respectively (26).	Excellent sensitivity (>80%) and specificity (>80%) in children who reported clinically significant levels of pain (26).	The minimal training required for parents to use this tool supports its utility as a clinical tool (26).	The items were developed based on parent reports and thus could miss important behavior changes in children (26).
Fear of pain	Parent Fear of Pain Questionnaire (27)	The 21-item scale showed a Cronbach's α = .91(27).	Solid construct validity, supported for the subscales (27).	Excellent fit for a complex model, after a minor modification to include parent avoidance as an indicator of parent behavior (27).	Tested in samples of predominantly mothers (92%), Caucasian (90%) and female children (74.8%) (27).
Pain cata-strophizing *	Pain Cata-strophizing Scale – 3 item version (10)	Chronbach's Alpha high α = .79 and .73 for PCS-P state (10).	Significant relationships with parent pain-related outcomes (i.e., parental distress), no issue with multicollinearity (10).	Short (3-item scale) can be efficiently used (= high correlation with 6-item scale). Large, diverse sample of parents (10).	Due to small number of items per factor, additional research needed to support the 1-factor model (10).
Anxiety and depression*	Anxiety and Depression Scale (28)	Cronbach's alpha high α = .78 for the depression scale and α = .81 for the anxiety scale (29).	Original model (28) demonstrates the best fit. Good discriminant validity in large study and across various populations and countries (29).	Simple and reliable tool for medical practice. In addition to validation for use in the elderly the HADS has been validated for use in adolescents (28).	Few studies validated the questionnaire with a general population or included a control group taken from

					the general population (29).
Sensory processing sensitivity	Highly Sensitive Person Scale (30,31)	Reliability of 27-item version Cronbach's α = .87 and .85 in 2 different studies (31).	The 27-item version showed good content validity and the measure's discriminant, convergent (31). Study confirmed that the HSP scale reflects a unitary dimension of environmental sensitivity and identified in addition three sensitivity groups in the general population (high, low and medium sensitivity) (32).	Diversity of samples as well as qualitative and quantitative methods used to develop scale and support conlcusions (31).	Parental impact on sensitive individuals not explored in depth (31).
Emotion regulation*	Emotion Regulation Questionnaire (33)	Cronbach's alpha satisfactory α = .74 for cognitive reappraisal and .76 for expressive suppression (34).	Indices of CFA were satisfactory for the German sample; support for measurement invariance of the ERQ (35).	Good conformity of the translation with the original (34).	Mainly tested in student samples (36).
Quality of life	World Health Organization Well-Being Index (37)	Internal consistency of the WHO-Five and the Mental Health subscale acceptable (Cronbach's α = 0.84. and 0.8) (37).	Adequate validity both as a screening tool for depression and as an outcome measure in clinical trials (38).	Includes subscales measuring both physical health and mental health. Compare a scale containing a mixture of distress and well being items (37). Validated in both younger and	Has been researched predominantly in the field of depression (38).

		elderly persons (38).
**Primary outcome**		
Chronic postsurgical pain	Presence of CPSP: pain diary for 7 consecutive days (NRS) + health-related quality of life (PedsQL)	

Note. AUC = area under the curve , ICC = intraclass correlation coefficient, PCS-C = Pain Catastrophizing Scale for Children, PCS-P = Pain Catastrophizing Scale for Parents.

References

1. Watson D, Clark LA, Tellegen A. Development and validation of brief measures of positive and negative affect: The PANAS scales. J Pers Soc Psychol. 1988;54(6):1063–70.
2. Flores-Kanter PE, Garrido LE, Moretti LS, Medrano LA. A modern network approach to revisiting the Positive and Negative Affective Schedule (PANAS) construct validity. J Clin Psychol. 2021;77(10):2370–404.
3. Barke A, Koechlin H, Korwisi B, Locher C. Emotional distress: Specifying a neglected part of chronic pain. Eur J Pain. 2020;24(3):477–80.
4. Treede RD, Rief W, Barke A, Aziz Q, Bennett MI, Benoliel R, et al. Chronic pain as a symptom or a disease: the IASP Classification of Chronic Pain for the International Classification of Diseases (ICD-11). PAIN. 2019 Jan;160(1):19.
5. McGrath PJ, Walco GA, Turk DC, Dworkin RH, Brown MT, Davidson K, et al. Core Outcome Domains and Measures for Pediatric Acute and Chronic/Recurrent Pain Clinical Trials: PedIMMPACT Recommendations. J Pain. 2008 Sep 1;9(9):771–83.
6. Simons LE, Smith A, Ibagon C, Coakley R, Logan DE, Schechter N, et al. Pediatric Pain Screening Tool (PPST): Rapid identification of risk in youth with pain complaints. Pain. 2015 Aug;156(8):1511–8.
7. Narayanasamy S, Yang F, Ding L, Geisler K, Glynn S, Ganesh A, et al. Pediatric Pain Screening Tool: A Simple 9-Item Questionnaire Predicts Functional and Chronic Postsurgical Pain Outcomes After Major Musculoskeletal Surgeries. J Pain [Internet]. 2021 Jul 17 [cited 2021 Sep 24]; Available from: https://www.sciencedirect.com/science/article/pii/S1526590021002911
8. Offenbächer M, Kohls N, Walker L, Hermann C, Hügle B, Jäger N, et al. Functional limitations in children and adolescents suffering from chronic pain: validation and psychometric properties of the German Functional Disability Inventory (FDI-G). Rheumatol Int. 2016 Oct 1;36(10):1439–48.
9. Flack F, Gerlach AL, Simons LE, Zernikow B, Hechler T. Validation of the German fear of pain questionnaire in a sample of children with mixed chronic pain conditions. Eur J Pain Lond Engl. 2017;21(7):1224–33.
10. Durand H, Birnie KA, Noel M, Vervoort T, Goubert L, Boerner KE, et al. State Versus Trait: Validating State Assessment of Child and Parental Catastrophic Thinking About Children's Acute Pain. J Pain. 2017 Apr 1;18(4):385–95.
11. Kerns KA, Klepac L, Cole A. Peer relationships and preadolescents' perceptions of security in the child-mother relationship. Dev Psychol. 1996 May;32(3):457–66.
12. Brumariu LE, Madigan S, Giuseppone KR, Movahed Abtahi M, Kerns KA. The Security Scale as a measure of attachment: Meta-analytic evidence of validity. Attach Hum Dev. 2018;20(6):600–25.
13. Ebesutani C, Reise SP, Chorpita BF, Ale C, Regan J, Young J, et al. The Revised Child Anxiety and Depression Scale-Short Version: Scale reduction via exploratory bifactor modeling of the broad anxiety factor. Psychol Assess. 2012;24(4):833–45.
14. Buysse DJ, Reynolds CFI, Monk TH, Berman SR, Kupfer DJ. The Pittsburgh Sleep Quality Index: A new instrument for psychiatric practice and research. Psychiatry Res. 1988;28:193–213.
15. Pluess M, Assary E, Lionetti F, Lester KJ, Krapohl E, Aron EN, et al. Environmental sensitivity in children: Development of the Highly Sensitive Child Scale and identification of sensitivity groups. Dev Psychol. 2018;54(1):51–70.

16. Koechlin H, Donado C, Locher C, Kossowsky J, Lionetti F, Pluess M. Sensory processing sensitivity in adolescents reporting chronic pain: an exploratory study. Pain Rep. 2023 Jan 6;8(1):e1053.
17. Gullone E, Taffe J. The Emotion Regulation Questionnaire for Children and Adolescents (ERQ-CA): a psychometric evaluation. Psychol Assess. 2012 Jun;24(2):409–17.
18. Abler B, Kessler H. Emotion Regulation Questionnaire – Eine deutschsprachige Fassung des ERQ von Gross und John. Diagnostica. 2009 Jul 1;55(3):144–52.
19. Koechlin H, Beeckman M, Meier AH, Locher C, Goubert L, Kossowsky J, et al. Association of parental and adolescent emotion-related factors with adolescent chronic pain behaviors. PAIN [Internet]. 2022 [cited 2021 Oct 28]; Available from: https://journals.lww.com/pain/Abstract/9000/Association_of_parental_and_adolescent.97860.aspx
20. Dorn C, Spindler G, Kullik A, Petermann F, Barnow S. Erfassung von Emotionsregulationsstrategien–eine Übersicht. Psychol Rundsch. 2013 Oct;64(4):217–27.
21. Varni JW, Seid M, Rode CA. The PedsQL™: Measurement Model for the Pediatric Quality of Life Inventory. Med Care. 1999 Feb;37(2):126.
22. Gordon-Hollingsworth AT, Thompson JE, Geary MA, Schexnaildre MA, Lai BS, Kelley ML. Social Support Questionnaire for Children. Meas Eval Couns Dev. 2016 Apr 1;49(2):122–44.
23. Gordon-Hollingsworth AT, Thompson JE, Geary MA, Schexnaildre MA, Lai BS, Kelley ML. Social Support Questionnaire for Children: Development and Initial Validation. Meas Eval Couns Dev. 2016 Apr;49(2):122–44.
24. Sachser C, Berliner L, Risch E, Rosner R, Birkeland MS, Eilers R, et al. The child and Adolescent Trauma Screen 2 (CATS-2) – validation of an instrument to measure DSM-5 and ICD-11 PTSD and complex PTSD in children and adolescents. Eur J Psychotraumatology. 2022 Dec 19;13(2):2105580.
25. Sachser C, Berliner L, Holt T, Jensen TK, Jungbluth N, Risch E, et al. International development and psychometric properties of the Child and Adolescent Trauma Screen (CATS). J Affect Disord. 2017 Mar 1;210:189–95.
26. Chambers CT, Reid GJ, McGrath PJ, Finley GA. Development and preliminary validation of a postoperative pain measure for parents 11This paper was presented at the meeting Pediatric Pain: Challenges, Innovations, and Costs in Atlanta, GA, November 4, 1995 (Voted Best Abstract). Pain. 1996 Dec 1;68(2):307–13.
27. Simons LE, Smith A, Kaczynski K, Basch M. Living in fear of your child's pain: the Parent Fear of Pain Questionnaire. Pain. 2015 Apr;156(4):694–702.
28. Snaith RP. The Hospital Anxiety And Depression Scale. Health Qual Life Outcomes. 2003 Aug 1;1(1):29.
29. Bocéréan C, Dupret E. A validation study of the Hospital Anxiety and Depression Scale (HADS) in a large sample of French employees. BMC Psychiatry. 2014;14(1):1–11.
30. Pluess M. Sensory-Processing Sensitivity: A potential mechanism of differential susceptibility. In: Biennial Meeting of the Society for Research in Child Development (SRCD), Seattle, WA. 2013.

31. Aron EN, Aron A. Sensory-processing sensitivity and its relation to introversion and emotionality. J Pers Soc Psychol. 1997 Aug;73(2):345–68.
32. Lionetti F, Aron A, Aron EN, Burns GL, Jagiellowicz J, Pluess M. Dandelions, tulips and orchids: Evidence for the existence of low-sensitive, medium-sensitive and high-sensitive individuals. Transl Psychiatry. 2018;8(1):24.
33. Gross JJ, John OP. Individual differences in two emotion regulation processes: implications for affect, relationships, and well-being. J Pers Soc Psychol. 2003 Aug;85(2):348–62.
34. Abler B, Kessler H. Emotion regulation questionnaire–Eine deutschsprachige Fassung des ERQ von Gross und John. Diagnostica. 2009;55(3):144–52.
35. Sala MN, Molina P, Abler B, Kessler H, Vanbrabant L, van de Schoot R. Measurement invariance of the Emotion Regulation Questionnaire (ERQ). A cross-national validity study. Eur J Dev Psychol. 2012;9(6):751–7.
36. Preece DA, Becerra R, Robinson K, Gross JJ. The emotion regulation questionnaire: psychometric properties in general community samples. J Pers Assess. 2019;
37. Bech P, Olsen LR, Kjoller M, Rasmussen NK. Measuring well-being rather than the absence of distress symptoms: a comparison of the SF-36 Mental Health subscale and the WHO-Five well-being scale. Int J Methods Psychiatr Res. 2003;12(2):85–91.
38. Topp CW, Østergaard SD, Søndergaard S, Bech P. The WHO-5 Well-Being Index: a systematic review of the literature. Psychother Psychosom. 2015;84(3):167–76.
