# Supplementary material for: Longitudinal Resilience and Risk Factors in Pediatric Postoperative Pain (LORRIS): Protocol for a Prospective Longitudinal Swiss University Children’s Hospitals-Based Study
Source: BMJ Open. 2024 Mar 28;14(3):e080174. doi: 10.1136/bmjopen-2023-080174 (PMC10982766; doi:10.1136/bmjopen-2023-080174)
Supplement: Author's manuscript [file bmjopen-2023-080174.draft_revisions.pdf]

BMJ Open

BMJ Open is committed to open peer review. As part of this commitment we make the peer review history of every article we publish publicly available.

When an article is published we post the peer reviewers' comments and the authors' responses online. We also post the versions of the paper that were used during peer review. These are the versions that the peer review comments apply to.

The versions of the paper that follow are the versions that were submitted during the peer review process. They are not the versions of record or the final published versions. They should not be cited or distributed as the published version of this manuscript.

BMJ Open is an open access journal and the full, final, typeset and author-corrected version of record of the manuscript is available on our site with no access controls, subscription charges or pay-per-view fees (http://bmjopen.bmj.com).

If you have any questions on BMJ Open's open peer review process please email info.bmjopen@bmj.com

BMJ Open

Longitudinal Resilience and Risk Factors in Pediatric Postoperative Pain (LORRIS):Protocol for a Prospective Longitudinal Study

Journal:	*BMJ Open*
Manuscript ID	bmjopen-2023-080174
Article Type:	Protocol
Date Submitted by the Author:	22-Sep-2023
Complete List of Authors:	Hochreuter, Jana; University Children's Hospital Zurich, Department of Psychosomatics and Psychiatry; University of Zurich, Department of Psychology<br>Dreher, Thomas ; University Children's Hospital Zürich, Department of Pediatric Orthopedics and Traumatology; Balgrist University Hospital, Department of Pediatric Orthopedics<br>Hasler, Carol-Claudius; Children’s Hospital Basel, Department of Orthopedics<br>Canonica, Sandro; University Children's Hospital Zürich, Pediatric Orthopedics and Traumatology; University Hospital Balgrist Zürich, Department of Pediatric Orthopedics<br>Locher, Cosima; University Hospital Zurich, Department of Consultation-Liaison Psychiatry and Psychosomatic Medicine; Plymouth University<br>Held, Ulrike; Epidemiology, Biostatistics and Prevention Institute, Biostatistics<br>Rabbitts, Jennifer; Stanford University, Anesthesiology Pediatric Anesthesiology<br>Koechlin, Helen; University Children's Hospital Zürich, Psychosomatics and Psychiatry; University of Zurich, Department of Psychology
Keywords:	SURGERY, Adolescents < Adolescent, Chronic Pain, Risk Factors

SCHOLARONE™
Manuscripts

Longitudinal Resilience and Risk Factors in Pediatric Postoperative Pain (LORRIS): Protocol for a Prospective Longitudinal Study

Hochreuter, J.[1,2,3], Dreher, T.[3,4,5], Hasler, C.-C.[6], Canonica, S.[3,4,5], Locher, C.[7,8], Held, U.[9], Rabbitts, J.[10], & Koechlin, H.[1,2,3]

[1]Department of Psychosomatics and Psychiatry, University Children's Hospital, University of Zurich, Zurich, Switzerland.

[2]Division of Child and Adolescent Health Psychology, Department of Psychology, University of Zurich, Zurich, Switzerland.

[3]Children's Research Centre, University Children's Hospital Zurich, University of Zurich, Zurich, Switzerland.

[4]Department of Pediatric Orthopedics and Traumatology, University Children's Hospital Zurich, Switzerland.

[5]Department of Pediatric Orthopedics, Orthopedic University Hospital Balgrist, Zurich, Switzerland

[6]Department of Orthopedics, Children's Hospital Basel, Switzerland.

[7]Department of Consultation-Liaison Psychiatry and Psychosomatic Medicine, University Hospital Zurich, Zurich, Switzerland.

[8]Faculty of Health, University of Plymouth, UK.

[9]Department of Biostatistics and Epidemiology, Biostatistics and Prevention Institute, University of Zurich, Switzerland.

[10]Anesthesiology, Perioperative and Pain Medicine, Pediatric Anesthesiology, Stanford University, USA.

For peer review only

Abstract

Introduction

Chronic postsurgical pain (CPSP) is defined as pain that persists after a surgical procedure, and has significant impact on quality of life. Previous studies show the importance of psychological factors in CPSP, yet there has been limited focus on the role of emotions. This longitudinal observational study aims to examine the role of emotional state, emotion variability, emotion regulation, and emotion differentiation on the child- and the parent-level for the development CPSP, and to describe pain and emotion-related trajectories following surgery.

Methods and analysis

Children and adolescents aged 8-18 years with a planned orthopedic surgery and their parents will be recruited. A total of five assessment time points is planned: 3 weeks before surgery (baseline), 2 weeks after surgery (post), and 3 (Follow-up [FU] 1), 6 and 12 months after surgery. At baseline and post, children and parents are asked to complete a daily diary where they rate their current emotional state and their pain severity (children only). Emotional state ratings will be used to calculate indeces of emotion variability, emotion regulation, and emotion differentiation. Children and parents will complete questionnaires at each time point, including measures on quality of life, social support, sleep, and symptoms of anxiety and depression.

To predict development of CPSP, generalized linear regression models will be used, resulting in odds ratios and 95% confidence intervals. Pearson product-moment correlations between predictors and outcomes will be evaluated at each time point. The primary outcome of the prediction model is CPSP at FU1. For the trajectory analysis, the classification method K-means for longitudinal data will be used to determine clusters in the data.

Ethics and dissemination

The Ethics Committee of the Canton of Zurich, Switzerland, is currently reviewing the study (ID: 2023-01475). Participants will be compensated, and a dissemination workshop will be held.

Registration details

Clinicaltrials.gov: NCT05816174

For peer review only

Strengths and limitations of this study

- Emotions and emotion-related factors are known to be closely associated with (chronic) pain. So far, most perioperative research has focused on negative emotions.
- This is the first study to specifically examine the role of emotion-related factors on the child and the parent level in the development of chronic pain after surgery.
- A longitudinal, prospective design allows to describe pain- and emotion-related trajectories up until 12 months after surgery.
- Repeated assessments design will lead to an in-depth understanding of changes of risk and resilience factors over time.

For peer review only

Introduction

Chronic postsurgical pain (CPSP) is defined as chronic pain that persists over time (at least 3 months) following a surgical procedure, and that has significant impact on physical and psychosocial quality of life (1,2). Meta-analytic estimates in children and adolescents describe that around 20% of pediatric patients undergoing major surgery report chronic pain three to twelve months after surgery (2). Highest pain intensity ratings are observed in two-weeks following surgery, with a steady decrease afterwards over the following six months (3), and pain ratings two weeks after surgery have been shown to predict the development of moderate to severe pain one year later (4,5). Further, most children and adolescents experience decreases in their physical activity levels following surgery, and it has been suggested that objectively monitored physical activity levels could be useful to indirectly assess functional recovery (6). A recent study in adolescents undergoing spinal fusion found that a majority of participants were characterized by a steep decrease of physical activity after surgery (7).

Previous research has identified psychosocial risk factors that are associated with the development of CPSP, among them presurgical pain intensity, child anxiety, child pain coping efficacy, child sleep patterns, and parental pain catastrophizing (2,8,9). Generally, emotions and emotion-related factors are known to influence pain, pain-related disability, and pain-related distress (10,11). While there is some research on the role of emotion regulation in chronic pain (see (12) for a review across age groups), and the role of negative emotions is fairly well-documented in the pain experience (10,13,14), emotion-related factors have yet to be studied in the context of pediatric CPSP.

Emotion variability

Emotion variability describes the range of emotional fluctuations around an individual's average emotional intensity (15,16). Greater emotion variability has been linked to more mental health symptoms in adolescents (17,18), and higher emotion regulation demands (19). In pediatric chronic pain, one study found greater positive emotion variability to be associated with less pain-related interference and more engagement in activities despite pain (16). Emotion variability can be measured using the within-person standard deviation of repeated assessments of current emotional state (16,18,20,21).

Emotion differentiation

Emotion differentiation defines the level of specificity people use when identifying their emotional experiences (22–24). Higher emotion differentiation indicates greater ability to differentiate between one's own emotional states on a more fine-grained level and by using precise terms (24). High or low emotion differentiation can be calculated by means of intraclass

For peer review only

correlations, where high consistency in emotion ratings across a predefined episode (e.g., a week-long daily diary phase) suggests poorer differentiation (24,25). Previous research has demonstrated that higher emotion differentiation is associated with better mental health (see (26) for a meta-analysis). To date, emotion differentiation has not been studied in the context of pediatric chronic pain.

Emotion regulation

Emotion regulation refers to a person's ability to influence what emotions they have, when they have them, and how they experience and express these emotions (27,28). A handful of previous studies have explored the role of emotion regulation in pediatric chronic pain (e.g., (16,20,29,30), yielding mixed results: While some found that higher emotion regulation was associated with more frequent hospitalization due to pain (29), and lower engagement in activities despite the pain (16), others did not identify significant associations between emotion regulation and pain intensity or pain-related disability (20,30). The habitual use of specific emotion regulation strategies is considered stable within individuals (31–33), and can be measured by means of questionnaires (19).

Familial influences on pediatric pain

Previous research has shown that around 50% of children and adolescents with chronic pain have a parent who has had chronic pain in the past or is currently living with it (34). Parents and the family environment have been identified as a "key context" (p. 702) regarding the understanding, assessment, and management of pediatric pain (35), and a bidirectional relationship between child functioning and parent factors is suggested (35–37). Regarding emotion-related factors, the family context is considered crucial: Children learn the appropriateness of valence, duration, and intensity of emotions and emotion expression by observing their parents and parents' emotion regulation behaviors (38,39).

Objectives

The aim of this observational longitudinal study is to specifically examine the potential role of emotion-related factors (namely emotion variability, emotion differentiation, and emotion regulation), as well as familial influences in the development of CPSP and describe pain- and emotion-related trajectories following major surgery up to 12 months after surgery. We aim to identify whether and which emotion-related factors increase or decrease the risk for the development of CPSP after planned orthopedic surgery. To the best of our knowledge, this is the first study to specifically examine these emotion-related factors as both potential risk *and* resilience factors in the development of CPSP

For peer review only

Methods and Analysis

Sample and Sample Size Calculation

The anticipated prevalence rate of CPSP is 20%, based on international estimates (2,40). The aim of this study is to recruit a sample of 281 child-parent dyads, based on a power calculation for a logistic regression model examining 13 predictor variables with CPSP status as the primary outcome. This sample size is based on the recommended number of events per variable recommended for logistic regression models (41). To achieve a large enough sample size, this number then needs to be doubled. With 13 primary predictors (see Table 1), the most conservative estimate is N=234; adding an expected drop-out rate of around 20% (based on similar previous longitudinal studies, (7,42,43)) results in this sample size.

Eligibility criteria

We will include participants between 8 and 18 years who have a planned in-patient orthopedic surgery at one of the study centers. The child-parent dyads must be able to read (for younger children: with help) and understand German. Exclusion criteria will be serious comorbid health condition (e.g., cancer, severe neurological impairment, chronic illness requiring daily medication), and prior major surgery (e.g., prior spine surgery). Mental health comorbidities are no reason for exclusion.

Study Design

This is a prospective longitudinal observational study in patients undergoing scheduled orthopedic surgery and their parents. As these orthopedic surgeries are planned well in advance, patients can be approached early and asked about their interest in participating in the study. See Figure 1 for an overview of assessment time points. The study is registered at clinicaltrials.gov (NCT05816174).

Procedures

Potential participants will be informed about the study by the clinical collaborators at each study site during one of their pre-surgical visits (e.g., anesthesia preparation interview, scheduling visit). Based on the clinical routine of the study centers, the procedures might differ slightly. If patients fall within the required age range, the clinical collaborators will provide the family with a leaflet with the most important study information. On the leaflet, a QR-Code brings families directly to a site on the website of the University Children's Hospital Zurich (main study center), where they can find the most important information again. If families express their initial interest about the study, the physician will take a note and inform the study team, which will then contact the family. A member of the study team will then approach the respective family at their next visit at the hospital and go through the

For peer review only

informed consent / assent procedure with them. In participating hospitals, patients typically have (at least) two visits before the surgery. Once the written informed consent and assent is given, families will complete a battery of questionnaires as baseline measures before the surgery via direct link to the online questionnaires (see next section and Table 1 for an overview), using Research Electronic Data Capture (RedCap; (44)). In addition, participants will be asked to download the smartphone application SEMA 3 (45), a free tool for ecological momentary assessment provided by the University of Melbourne, Australia, especially developed for research needs.

Families will be called again two weeks after the surgery, and asked to complete the post-assessment via direct link to the questionnaires. Families will be contacted again 3 months (FU 1), 6 months (FU 2), and 12 months (FU 3) after surgery to complete the follow-up surveys, and asked to complete the surveys at each assessment point within one week after receiving the link to the survey; up to three weekly reminders will be sent after the initial message.

In addition to the questionnaires at each assessment point, all participants (i.e., children and parents) will complete daily diaries: To estimate the influence of emotion-related factors on pain trajectories, children and adolescents will complete two daily diary phases (i.e., two weeks of daily diary) during which they rate both their pain severity and emotional state (referred to as "pain and emotions" throughout the protocol; at baseline and post assessment), and one daily diary episode during which they only rate their pain intensity (referred to as "pain only" throughout the protocol; at Follow-up 1. See Figure 1). Parents will complete two daily diary phases (at baseline and post) during which they exclusively rate their emotional state.

Daily diary - pain and emotions (two timepoints): At baseline and post assessment, children and adolescents will complete ratings of their pain severity on an 11-point numeric rating scale with anchors 0 = "no pain" and 10 = "worst pain possible". Pain severity includes pain intensity, pain-related distress, and pain-related interference; hence, participants will give three different ratings (41,42). The pain ratings will be used to classify the presence of acute postsurgical pain at the post assessment (in combination with a health-related quality of life rating; see overview of questionnaires for this time point in Table 1). In addition, all participants will rate the extent to which they experience positive and negative emotions three times each day on their smartphone and during their normal daily routine over the course of 7 days. These ratings will be used to calculate a score of emotion differentiation (i.e., the ability to differentiate between emotional states), a mean value of positive and negative emotions,

For peer review only

and an index of emotional variability (26,36,37; see below for more detailed description). The items of the short version of the Positive and Negative Affect Schedule for Children (PANAS-C) will be used to assess emotional state (38–40).

Daily diary - pain only (once): To diagnose CPSP, participants will be asked to rate their daily pain severity for 7 days starting at FU1. Participants will be prompted at the end of each day to respond on an 11-point numeric rating scale with anchors 0 = “no pain” and 10 = “worst pain possible”. In addition, daily medication use will be indicated (Yes/No and listing medication in a free text field).

Number of steps taken each day will be counted as an indicator of changes in physical activity before and after surgery (i.e., at the post assessment), measured by means of accelerometer (GENEActive, Activeinsights Ltd), an ambulatory, non-invasive activity-monitoring device that registers physical activity, and can be worn around the wrist.

Study Measures

See Table 1 for an overview. For all measures, we use the validated German version (if available) or translate and back-translate the items with the help of a professional interpreter or native speaker. Measures of primary interest are marked with an * in Table 1, all other variables are considered exploratory.

Child Measures

Emotional state: The Positive and Negative Affect Schedule for Children (PANAS-C) (46–48) is a measures of positive and negative affect by providing mood adjectives (e.g. “scared”, “happy”). Participants are asked to rate 10 items on a 5-point Likert-scale from 1 (= very slightly or not at all) to 5 (= very much) to assess the extent to which they currently feel each of 5 positively and 5 negatively valenced emotions.

Pain Screening Tool: The Pediatric Pain Screening Tool (PPST) (49,50) entails 9 self-reported items that belong to a physical and psychosocial subscale. Answers are given dichotomously (disagree = 0, agree = 1). Higher scores indicate higher risk status of poor pain-related outcomes.

Fear of Pain: The Fear of Pain Questionnaire for Children (FOPQ-C) (51) is a 24-item self-report inventory to assess fear of pain. Participants are asked to rate each item on a Likert-

For peer review only

scale from 0 (= strongly disagree) to 4 (= strongly agree). A higher total score indicates higher fear.

Functional Disability: Children's self-reported difficulty in physical and psychosocial functioning due to their physical health is assessed by the Functional Disability Inventory (FDI) (52). 15 items are rated on a Likert-scale from 0 (= no problems) to 4 (= not possible) and concern perceptions of activity limitations during the past 2 weeks. Higher scores indicate greater disability.

Security in Parent-Child Relationships: The Security Scale (53) consists of 15 items and assesses participants' perceptions of security in parent-child relationships. Items are presented in a format of statements, participants choose which statement is more characteristic of them and then rate the statement on a Likert-scale from 1 (= really true) to 4 (= sort of true). Higher scores indicate a more secure attachment.

Symptoms of Anxiety and Depression: The RCADS-short version (54) is a 25-item self-report questionnaire used to assess symptoms of depression and anxiety. Participants are asked to indicate how often each item applies to them according to a 4-point Likert-scale from 0 (= never) to 3 (= always). High scores indicate high anxiety and depression symptoms.

Sleep: The Pittsburgh Sleep Quality Index (PSQI) (55) is the most commonly used retrospective self-report questionnaire that measures sleep quality over the previous month. We will use a version that has been slightly adapted for children by researchers of the University Children's Hospital. This version contains of 25 items, and each item is rated on a scale from 0 (= no difficulty) to 3 (= severe difficulty). In addition to a global sleep quality factor, seven domains of sleep difficulties can be assessed: Sleep quality, sleep latency, sleep duration, habitual sleep efficiency, sleep disturbances, use of sleeping medications, and daytime dysfunction. A higher global score indicates more sleep difficulties.

Sensory Processing Sensitivity: The Highly Sensitive Child Scale (HSC) (56) is a 12-item self-reported questionnaire. Participants are asked to rate each item on a Likert-scale from 1 (= not at all) to 7 (= extremely). In addition to a total score, three subscales can be calculated: Low Sensory Threshold (LST; 3 items, e.g., "I don't like watching TV programs that have a lot of violence in them"), Ease of Excitation (EOE; 5 items, e.g., "I get nervous when I have

For peer review only

to do a lot in little time”), and Aesthetic Sensitivity (AES; 4 items, e.g., “I love nice smells”). Higher scores indicate higher sensitivity.

Emotion Regulation: The Emotion Regulation Questionnaire for Children and Adolescents (ERQ-CA) (19) is a self-report questionnaire containing 10 items. Items are rated on a Likert-scale from 1 (= strongly disagree) to 7 (= strongly agree), and assess individual differences in the habitual use of two emotion regulation strategies, namely Cognitive Reappraisal (e.g., “When I’m faced with a stressful situation, I make myself think about it in a way that helps me stay calm”) and Expressive Suppression (e.g., “I keep my emotions to myself”). Higher scores indicate more habitual use of the respective emotion regulation strategy.

Quality of Life: The Pediatric Quality of Life Inventory (PedsQL) (57) is a self-or parent-reported questionnaire for children and adolescents. 23 Items are rated on a Likert-scale from 0 (= never a problem) to 4 (= almost always a problem). The questionnaire consists of the subscales physical functioning (e.g., “It is hard for me to run”), emotional functioning (e.g., “I feel sad or blue”), social functioning (e.g., “It is hard to keep up with my peers”), and school functioning (e.g., “It is hard to pay attention in class”) in the past month. Higher scores indicate better quality of life.

Social Support: The Social Support Questionnaire for Children (SSQC) (58) consists of 50 items. It measures children’s social support across five distinct sources of support: parents, relatives, nonrelative adults, siblings, and peers. Items are rated on a 4-point Likert-scale ranging from 0 (= never or rarely true) to 3 (= often or always true). Higher scores indicate higher levels of perceived support.

Symptoms of Posttraumatic Stress: The Child and Adolescent Trauma Screen 2 (CATS-2) (59) contains a checklist of 15 potentially traumatic events (PTEs), followed by an item asking which of the PTEs bothers them most. Posttraumatic stress symptoms are measured by 20 items that are rated on a 4-point Likert-scale ranging from 0 (= never) to 3 (= almost always). Five yes/no items assess whether the previously rated posttraumatic stress symptoms interfere with five key areas of functioning and are used to assess psychosocial functioning.

<u>Parent Measures</u>

For peer review only

Postoperative Pain: With the Postoperative Pain Measure for Parents (PPMP) (60), parents can assess and estimate their child's postoperative pain. The checklist includes 15 items where the parents select “yes” (= 1 point) or “no” (= 0 points) as to whether the child exhibits pain-related behavior, with higher scores indicating more postoperative pain.

The Postoperative Pain Measure for Parents (PPMP) [illegible]

Fear of Pain Questionnaire: The Parent Fear of Pain Questionnaire (FOPQ-P) (35) is a 23-item self-report inventory to reflect a parent's own fear associated with their child's pain experience. Parents are asked to rate each item on a Likert-scale from 0 (= strongly disagree) to 4 (= strongly agree). A higher total score indicates higher fear of pain.

Symptoms of Anxiety and Depression: The Hospital Anxiety and Depression Scale (HADS) (61) is a widely used measure of psychological distress, and indicates the presence of symptoms of depression (seven items) and anxiety (seven items) over a 1-week period. The 14 items are rated on a four-point Likert-scale ranging from 0 to 3, with higher scores indicate greater symptom severity.

Sensory Processing Sensitivity: The Highly Sensitive Person Scale (HSP) (62,63) is a 12-item self-reported questionnaire, and participants are asked to rate each item on a Likert-scale from 1 (= strongly disagree) to 7 (= strongly agree). In addition to a total score, three subscales can be calculated, as for the HSC Scale: Low Sensory Threshold, Ease of Excitation, and Aesthetic Sensitivity. Higher scores reflect higher sensitivity.

Emotion Regulation: The Emotion Regulation Questionnaire (ERQ) (31) assesses the habitual use of two emotion regulation strategies, namely Cognitive Reappraisal (6 items) and Expressive Suppression (4 items). The 10 self-reported items are rated on a 7-point Likert-scale ranging from 1 (= strongly disagree) to 7 (= strongly agree). Higher scores indicate higher usage of the respective strategy.

Well-Being: Parental well-being will be measured using he WHO-5 index (64). It consists of 5 items that are rated on a 6-point Likert-scale ranging from 0 (= never) to 5 (=always), indicating how often participants have felt in “good spirits”, “active”, “relaxed”, etc. over the course of the last 2 weeks. Higher scores indicate more well-being.

For peer review only

Table 1. Measures, Tools, Domain and Time-Points for Data Collection

Domain		Measure	Completed by	BL	Post	FU 1	FU 2	FU 3
**Demographics**	Age, gender, school, school attendance and performance; family constellation, parental educational level and work status	Parent and self-report: online baseline questionnaire	Parent(s) and child	X				
**Medical history**	Neonatal intensive care (yes/no), previous surgeries (yes/no; which one), child's pain history (pain duration, pain location)	Parent report: online baseline questionnaire	Parents	X				
	Complications in the hospital (yes/no) Pain treatment immediately following surgery	Child's medication list in electronic health record	Data entered by study team		X			
**Experience-sampling method**	Positive and negative emotions*	Items from the Positive and Negative Affect Schedule (53)	Daily diary: child and parent	X	X			
	Pain severity	Numeric Rating Scale (NRS), 0-10	Daily diary: child	X	X	X		
**Psychosocial factors child**								
	Pain severity (intensity, distress, interference (72))*	Numeric Rating Scale (NRS), 0-10	Child self-report				X	X
	Preoperative screening	Pediatric Pain Screening Tool (56,57)	Child self-report	X				
	Functional Disability	Functional Disability Index (FDI; (59))	Child self-report	X	X	X	X	X
	Fear of Pain*	Fear of Pain Questionnaire (58)	Child self-report	X				
	Pain catastrophizing*	Pain Catastrophizing scale – 3 item version (73)	Child self-report	X				
	Attachment; parent–child relationship*	Security Scale (74)	Child self-report	X				
	Symptoms of anxiety and depression*	Revised Child Anxiety and Depression Scale-short version (61)	Child self-report	X	X	X	X	X
	Sleep*	Pittsburgh Sleep Quality Index (PSQI; (62))	Child self-report	X	X	X	X	X
	Sensory processing sensitivity	Highly Sensitive Child Scale (63)	Child self-report	X				
	Emotion regulation*	Emotion Regulation Questionnaire – Child and Adolescent version (26)	Child self-report	X				
	Quality of life	Pediatric Quality of Life Inventory (64)	Child self-report	X	X	X	X	X

	Social support*	Social Support Questionnaire for Children (65)	Child self-report	X				
	Posttraumatic stress symptoms	Child and Adolescent Trauma Screening (CATS) (75)	Child self-report	X		X		
**Psychosocial factors parents**								
	Parents' own pain history*	2 questions: "(1) Have you ever and (2) are you currently living with persistent or recurrent pain for at least 3 months?"	Parent self-report	X				
	Postoperative pain measure (child's pain)	Postoperative Pain Measure for Parents (67)	Parent report		X			
	Fear of pain	Parent Fear of Pain Questionnaire (42)	Parent self-report	X				
	Pain catastrophizing*	Pain Catastrophizing Scale – 3 item version (73)	Parent self-report	X				
	Anxiety and depression*	Anxiety and Depression Scale (68)	Parent self-report	X	X	X	X	X
	Sensory processing sensitivity	Highly Sensitive Person Scale (70)	Parent self-report	X				
	Emotion regulation*	Emotion Regulation Questionnaire (38)	Parent self-report	X				
	Quality of life	World Health Organization Well-Being Index (71)	Parent self-report	X	X	X	X	X
**Primary outcome**								
	Chronic postsurgical pain	Presence of CPSP: pain diary for 7 consecutive days (NRS) + health-related quality of life (PedsQL, see above)				X		

Note. Primary predictors are marked with an *. Unmarked variables are considered secondary or exploratory

Primary Outcome

The primary endpoint is CPSP at FU 1. CPSP is defined as greater than minimal pain (pain intensity ≥ 3) on more than 50% of days as measured by daily diary over 7 days, and impairment in health-related quality of life (score of ≤ 74.9 on the *Pediatric Quality of Life Inventory*, PedsQL (57), i.e., 1 SD below the population mean based on normative US data). The combination of pain and quality of life measures will result in a binary variable (CPSP yes/no). This measurement of CPSP is supported by previous research (6,65,66), and a

modified definition of CPSP by the International Association for the Study of Pain (IASP) (67).

Secondary Outcomes

Secondary endpoints are trajectories of pain and emotion-related factors over time (i.e., baseline through FU3), number of steps taken by patients, pain severity, and quality of life. Postoperative complications will be monitored using the modified Clavien-Dindo Classification (68).

Statistical methods

Descriptive statistics will include mean and standard deviation or median and inter quartile range for normally distributed and non-normally distributed or ordinal variables measured at baseline. Categorical variables will be reported as number and percentage of total. The primary outcome is binary, therefore generalized linear regression models or generalized linear mixed effects models will be used, using a logit link function. The resulting estimands will be odds ratios and 95% confidence intervals. Variables at baseline will be used in the prediction model, with performance measures area under the curve (AUC), calibration, and scaled Brier Score (69,70). Variables measured consecutively over multiple days at each assessment interval will be averaged over time. We will use the rule of 10 events per variable (EPV) that would determine the number of independent variables in the regression model. Potentially time-varying covariates will be addressed. Potentially non-linear relationships of the independent variables with outcome will be evaluated.

We will conduct bivariate analyses to compare study completers versus study dropouts to detect potential differences regarding participant demographic between the groups. Baseline daily pain ratings will be averaged for each participant to calculate a mean baseline pain intensity score. Post assessment and FU 1 daily pain ratings will be combined with health-related quality of life data to define binary outcome variables for acute postsurgical pain and CPSP. Acute postsurgical pain and CPSP will be defined independently.

For the trajectory analysis, the classification method K-means for longitudinal data will be used to determine clusters in the data (R package kml) (71). The analysis of clusters joint trajectories (R package kml3d) (71) will be applied, as this analysis allows summarizing several correlated continuous variables into a single nominal variable (72).

A detailed statistical analysis plan will be written up and finalized before data export for analysis. All analyses will be performed with the R programming language in a fully scripted way and dynamic reporting will be used to guarantee highest level of reproducibility.

For peer review only

Monitoring

The study will be monitored for quality and regulatory adherence. The monitor, who is not involved in our study and supervised by the Clinical Trials Center of the University Hospital Zurich, Switzerland, will ensure that the specifications of the project plan and the associated processes are adhered to.

Ethics and Dissemination

All participants will be compensated for their time by means of a voucher. We will reimburse participating families per assessment time point, so that families who drop out will still receive a certain amount of compensation. The first few participating families will be asked about general accessibility of the study. Their feedback will be implemented in study procedures if necessary.

The risk of adverse effects due to the study are considered to be minimal. All project data will be handled with uttermost discretion and will only be accessible to the study team. Data will be stored on a secured and password-protected server.

This study is registered at clinicaltrials.gov (identification number: NCT05816174). After study completion, the study team organizes a dissemination workshop aiming at youth with CPSP and their parents, researchers, clinicians, and the interested public, to discuss study results, clinical implications, and formulate future research questions.

Discussion

The study presented here is longitudinal, prospective, and observational and aims to estimate the influence of a range of risk and resilience factors on the development of CPSP. Emotion-related factors such as those measured in this study can be screened for, and they present as good targets for training modules within the prevention and treatment of pediatric CPSP: For example, adaptive use of emotion regulation strategies can be trained, with positive results in previous studies that included emotion regulation in chronic pain treatment options (73–76). The inclusion of potentially protective factors might lead to novel and innovative clinical approaches in the future (77,78).

Chronic pain in childhood and adolescence tends to persist into adulthood (79–81), is associated with symptoms of psychopathology (82–85), and can even lead to first lifetime

For peer review only

onset suicidality (86). Better knowledge of the development of CPSP is therefore crucial to prevent the negative and long-lasting consequences.

For peer review only

Full references

1. Schug SA, Lavand'homme P, Barke A, Korwisi B, Rief W, Treede RD, et al. The IASP classification of chronic pain for ICD-11: chronic postsurgical or posttraumatic pain. Pain. 2019 Jan;160(1):45–52.
2. Rabbitts JA, Fisher E, Rosenbloom BN, Palermo TM. Prevalence and predictors of chronic postsurgical pain in children: A systematic review and meta-analysis. J Pain. 2017 Jun;18(6):605–14.
3. Connelly M, Fulmer RD, Prohaska J, Anson L, Dryer L, Thomas V, et al. Predictors of Postoperative Pain Trajectories in Adolescent Idiopathic Scoliosis. Spine. 2014 Feb 1;39(3):E174.
4. Pagé MG, Stinson J, Campbell F, Isaac L, Katz J. Identification of pain-related psychological risk factors for the development and maintenance of pediatric chronic postsurgical pain. J Pain Res. 2013 Mar 5;6:167–80.
5. Rosenbloom BN, Katz J. Modeling the transition from acute to chronic postsurgical pain in youth: A narrative review of epidemiologic, perioperative, and psychosocial factors. Canadian Journal of Pain. 2022 Jun 15;6(2):166–74.
6. Rabbitts JA, Zhou C, Groenewald CB, Durkin L, Palermo TM. Trajectories of postsurgical pain in children: risk factors and impact of late pain recovery on long-term health outcomes after major surgery. PAIN. 2015 Nov;156(11):2383–9.
7. Beeckman M, Hughes S, Van der Kaap-Deeder J, Plasschaert F, Michielsen J, Moens P, et al. Risk and Resilience Predictors of Recovery after Spinal Fusion Surgery in Adolescents. The Clinical Journal of Pain [Internet]. 2021 Aug 23 [cited 2021 Sep 8];Publish Ahead of Print. Available from: https://journals.lww.com/10.1097/AJP.0000000000000971
8. Katz J, Weinrib AZ, Clarke H. Chronic postsurgical pain: From risk factor identification to multidisciplinary management at the Toronto General Hospital Transitional Pain Service. Canadian Journal of Pain. 2019 Jul 15;3(2):49–58.
9. Rabbitts JA, Groenewald CB, Tai GG, Palermo TM. Presurgical psychosocial predictors of acute postsurgical pain and quality of life in children undergoing major surgery. J Pain. 2015 Mar;16(3):226–34.
10. Wiech K, Tracey I. The influence of negative emotions on pain: behavioral effects and neural mechanisms. Neuroimage. 2009 Sep;47(3):987–94.
11. Barke A, Koechlin H, Korwisi B, Locher C. Emotional distress: Specifying a neglected part of chronic pain. European Journal of Pain. 2020;24(3):477–80.
12. Koechlin H, Coakley R, Schechter N, Werner C, Kossowsky J. The role of emotion regulation in chronic pain: A systematic literature review. Journal of Psychosomatic Research. 2018 Apr 1;107:38–45.
13. Berna C, Leknes S, Holmes EA, Edwards RR, Goodwin GM, Tracey I. Induction of depressed mood disrupts emotion regulation neurocircuitry and enhances pain unpleasantness. Biological psychiatry. 2010 Jun 1;67(11):1083–90.
14. Janssen SA. Negative affect and sensitization to pain. Scand J Psychol. 2002 Apr;43(2):131–7.
15. Reitsema AM, Jeronimus BF, Dijk M van, Jonge P de. Emotion dynamics in children and adolescents: A meta-analytic and descriptive review. Emotion [Internet]. 2021 Jan [cited 2021 Apr 19]; Available from: https://research.rug.nl/en/publications/emotion-dynamics-in-children-and-adolescents-a-meta-analytic-and-
16. Koechlin H, Beeckman M, Meier AH, Locher C, Goubert L, Kossowsky J, et al. Association of parental and adolescent emotion-related factors with adolescent chronic pain behaviors. PAIN [Internet]. 2021 Oct 20 [cited 2021 Oct 28]; Available from: https://journals.lww.com/pain/Abstract/9000/Association_of_parental_and_adolescent.97860.aspx
17. Neumann A, van Lier PAC, Frijns T, Meeus W, Koot HM. Emotional Dynamics in the

Development of Early Adolescent Psychopathology: A One-Year Longitudinal Study. J Abnorm Child Psychol. 2011 Jul 1;39(5):657–69.
18. Silk JS, Steinberg L, Morris AS. Adolescents' Emotion Regulation in Daily Life: Links to Depressive Symptoms and Problem Behavior. Child Development. 2003;74(6):1869–80.
19. Gullone E, Taffe J. The Emotion Regulation Questionnaire for Children and Adolescents (ERQ-CA): a psychometric evaluation. Psychol Assess. 2012 Jun;24(2):409–17.
20. Connelly M, Bromberg MH, Anthony KK, Gil KM, Franks L, Schanberg LE. Emotion Regulation Predicts Pain and Functioning in Children With Juvenile Idiopathic Arthritis: An Electronic Diary Study. J Pediatr Psychol. 2012 Jan 1;37(1):43–52.
21. Kalokerinos EK, Murphy SC, Koval P, Bailen NH, Crombez G, Hollenstein T, et al. Neuroticism may not reflect emotional variability. Proceedings of the National Academy of Sciences. 2020 Apr 28;117(17):9270–6.
22. Barrett LF, Gross J, Christensen TC, Benvenuto M. Knowing what you're feeling and knowing what to do about it: Mapping the relation between emotion differentiation and emotion regulation. Cognition and Emotion. 2001 Nov 1;15(6):713–24.
23. Kashdan TB, Barrett LF, McKnight PE. Unpacking Emotion Differentiation: Transforming Unpleasant Experience by Perceiving Distinctions in Negativity. Curr Dir Psychol Sci. 2015 Feb 1;24(1):10–6.
24. Nook E. Emotion Differentiation and Youth Mental Health: Current Understanding and Open Questions [Internet]. PsyArXiv; 2021 [cited 2021 Apr 26]. Available from: https://psyarxiv.com/qkdbu/
25. Kalokerinos EK, Erbas Y, Ceulemans E, Kuppens P. Differentiate to Regulate: Low Negative Emotion Differentiation Is Associated With Ineffective Use but Not Selection of Emotion-Regulation Strategies. Psychol Sci. 2019 Jun;30(6):863–79.
26. Seah THS, Coifman KG. Emotion differentiation and behavioral dysregulation in clinical and nonclinical samples: A meta-analysis. Emotion. 2021;No Pagination Specified-No Pagination Specified.
27. Gross JJ. Emotion Regulation. In: Handbook of emotions. 3rd ed. New York: The Guilford Press; 2008.
28. Gross JJ. Emotion Regulation: Current Status and Future Prospects. Psychological Inquiry. 2015 Jan 2;26(1):1–26.
29. Tsao JC, Jacob E, Seidman LC, Lewis MA, Zeltzer LK. Psychological aspects and hospitalization for pain crises in youth with sickle-cell disease. J Health Psychol. 2014 Mar;19(3):407–16.
30. Koechlin H, Donado C, Locher C, Kossowsky J, Lionetti F, Pluess M. Sensory processing sensitivity in adolescents reporting chronic pain: an exploratory study. Pain Rep. 2023 Jan 6;8(1):e1053.
31. Gross JJ, John OP. Individual differences in two emotion regulation processes: implications for affect, relationships, and well-being. J Pers Soc Psychol. 2003 Aug;85(2):348–62.
32. English T, Lee IA, John OP, Gross JJ. Emotion regulation strategy selection in daily life: The role of social context and goals. Motiv Emot. 2017 Apr;41(2):230–42.
33. Brans K, Koval P, Verduyn P, Lim YL, Kuppens P. The regulation of negative and positive affect in daily life. Emotion. 2013 Oct;13(5):926–39.
34. Poppert Cordts KM, Stone AL, Beveridge JK, Wilson AC, Noel M. The (Parental) Whole Is Greater Than the Sum of Its Parts: A Multifactorial Model of Parent Factors in Pediatric Chronic Pain. J Pain. 2019 Jul;20(7):786–95.
35. Simons LE, Smith A, Kaczynski K, Basch M. Living in fear of your child's pain: the Parent Fear of Pain Questionnaire. Pain. 2015 Apr;156(4):694–702.
36. Chambers CT, Craig KD, Bennett SM. The Impact of Maternal Behavior on

For peer review only

Children's Pain Experiences: An Experimental Analysis. J Pediatr Psychol. 2002 Apr 1;27(3):293–301.
37. Palermo TM, Chambers CT. Parent and family factors in pediatric chronic pain and disability: An integrative approach. PAIN. 2005 Dec;119(1–3):1–4.
38. Bariola E, Gullone E, Hughes EK. Child and adolescent emotion regulation: the role of parental emotion regulation and expression. Clin Child Fam Psychol Rev. 2011 Jun;14(2):198–212.
39. Morris AS, Silk JS, Steinberg L, Myers SS, Robinson LR. The Role of the Family Context in the Development of Emotion Regulation. Soc Dev. 2007 May 1;16(2):361–88.
40. Williams G, Howard RF, Liossi C. Persistent postsurgical pain in children and young people: prediction, prevention, and management. Pain Rep. 2017 Aug 21;2(5):e616.
41. Vittinghoff E, McCulloch CE. Relaxing the Rule of Ten Events per Variable in Logistic and Cox Regression. American Journal of Epidemiology. 2007 Mar 15;165(6):710–8.
42. Carter BD, Kronenberger WG, Cruce-Spurling S, Threlkeld BM, Brady CE, Jones LC, et al. Factors associated with dropout versus completion of a manualized treatment for pediatric chronic pain. Clinical Practice in Pediatric Psychology. 2015;3(4):327–39.
43. Gustavson K, von Soest T, Karevold E, Røysamb E. Attrition and generalizability in longitudinal studies: findings from a 15-year population-based study and a Monte Carlo simulation study. BMC Public Health. 2012 Oct 29;12(1):918.
44. Harris PA, Taylor R, Thielke R, Payne J, Gonzalez N, Conde JG. Research electronic data capture (REDCap) - A metadata-driven methodology and workflow process for providing translational research informatics support. Journal of Biomedical Informatics. 2009;42(2):377–81.
45. Koval P, Hinton J, Dozo N, Gleeson J, Alvarez M, Harrison A, et al. SEMA3: Smartphone Ecological Momentary Assessment. 2019.
46. Watson D, Clark LA, Tellegen A. Development and validation of brief measures of positive and negative affect: The PANAS scales. Journal of Personality and Social Psychology. 1988;54(6):1063–70.
47. Laurent J, Catanzaro SJ, Joiner Jr. TE, Rudolph KD, Potter KI, Lambert S, et al. A measure of positive and negative affect for children: Scale development and preliminary validation. Psychological Assessment. 1999;11(3):326–38.
48. Wilson K, Gullone E, Moss S. The Youth Version of the Positive and Negative Affect Schedule: A Psychometric Validation. Behaviour Change. 1998 Sep;15(3):187–93.
49. Simons LE, Smith A, Ibagon C, Coakley R, Logan DE, Schechter N, et al. Pediatric Pain Screening Tool (PPST): Rapid identification of risk in youth with pain complaints. Pain. 2015 Aug;156(8):1511–8.
50. Narayanasamy S, Yang F, Ding L, Geisler K, Glynn S, Ganesh A, et al. Pediatric Pain Screening Tool: A Simple 9-Item Questionnaire Predicts Functional and Chronic Postsurgical Pain Outcomes After Major Musculoskeletal Surgeries. The Journal of Pain [Internet]. 2021 Jul 17 [cited 2021 Sep 24]; Available from: https://www.sciencedirect.com/science/article/pii/S1526590021002911
51. Flack F, Gerlach AL, Simons LE, Zernikow B, Hechler T. Validation of the German fear of pain questionnaire in a sample of children with mixed chronic pain conditions. Eur J Pain. 2017;21(7):1224–33.
52. Offenbächer M, Kohls N, Walker L, Hermann C, Hügle B, Jäger N, et al. Functional limitations in children and adolescents suffering from chronic pain: validation and psychometric properties of the German Functional Disability Inventory (FDI-G). Rheumatol Int. 2016 Oct 1;36(10):1439–48.
53. Kerns KA, Klepac L, Cole A. Security Scale. Developmental Psychology [Internet]. 1996 [cited 2023 Apr 13]; Available from:

For peer review only

https://psycnet.apa.org/doiLanding?doi=10.1037%2Ft17560-000
54. Ebesutani C, Reise SP, Chorpita BF, Ale C, Regan J, Young J, et al. The Revised Child Anxiety and Depression Scale-Short Version: Scale reduction via exploratory bifactor modeling of the broad anxiety factor. Psychological Assessment. 2012;24(4):833–45.
55. Buysse DJ, Reynolds CFI, Monk TH, Berman SR, Kupfer DJ. The Pittsburgh Sleep Quality Index: A new instrument for psychiatric practice and research. Psychiatry Research. 1988;28:193–213.
56. Pluess M, Assary E, Lionetti F, Lester KJ, Krapohl E, Aron EN, et al. Environmental sensitivity in children: Development of the Highly Sensitive Child Scale and identification of sensitivity groups. Dev Psychol. 2018;54(1):51–70.
57. Varni JW, Seid M, Rode CA. The PedsQL™: Measurement Model for the Pediatric Quality of Life Inventory. Medical Care. 1999 Feb;37(2):126.
58. Gordon-Hollingsworth AT, Thompson JE, Geary MA, Schexnaildre MA, Lai BS, Kelley ML. Social Support Questionnaire for Children. Measurement and Evaluation in Counseling and Development. 2016 Apr 1;49(2):122–44.
59. Sachser C, Berliner L, Risch E, Rosner R, Birkeland MS, Eilers R, et al. The child and Adolescent Trauma Screen 2 (CATS-2) – validation of an instrument to measure DSM-5 and ICD-11 PTSD and complex PTSD in children and adolescents. European Journal of Psychotraumatology. 2022 Dec 19;13(2):2105580.
60. Chambers CT, Reid GJ, McGrath PJ, Finley GA. Development and preliminary validation of a postoperative pain measure for parents 11This paper was presented at the meeting Pediatric Pain: Challenges, Innovations, and Costs in Atlanta, GA, November 4, 1995 (Voted Best Abstract). Pain. 1996 Dec 1;68(2):307–13.
61. Snaith RP. The Hospital Anxiety And Depression Scale. Health and Quality of Life Outcomes. 2003 Aug 1;1(1):29.
62. Aron EN, Aron A. Sensory-processing sensitivity and its relation to introversion and emotionality. J Pers Soc Psychol. 1997 Aug;73(2):345–68.
63. Pluess M. Sensory-Processing Sensitivity: A potential mechanism of differential susceptibility. In: Biennial Meeting of the Society for Research in Child Development (SRCD), Seattle, WA. 2013.
64. Bech P, Olsen LR, Kjoller M, Rasmussen NK. Measuring well-being rather than the absence of distress symptoms: a comparison of the SF-36 Mental Health subscale and the WHO-Five well-being scale. International Journal of Methods in Psychiatric Research. 2003;12(2):85–91.
65. Rabbitts JA, Palermo TM, Zhou C, Meyyappan A, Chen L. Psychosocial Predictors of Acute and Chronic Pain in Adolescents Undergoing Major Musculoskeletal Surgery. The Journal of Pain. 2020 Nov 1;21(11):1236–46.
66. Rabbitts JA, Palermo TM, Zhou C, Mangione-Smith R. Pain and Health-Related Quality of Life After Pediatric Inpatient Surgery. The Journal of Pain. 2015 Dec 1;16(12):1334–41.
67. Werner MU, Kongsgaard UE. I. Defining persistent post-surgical pain: is an update required? Br J Anaesth. 2014 Jul;113(1):1–4.
68. Camino Willhuber G, Slullitel P, Taype Zamboni D, Albergo J, Terrasa S, Piuzzi N, et al. Validation of a modified Clavien-Dindo Classification for postoperative complications in orthopedic surgery. Rev Fac Cien Med Univ Nac Cordoba. 2020 Aug 21;77(3):161–7.
69. Steyerberg EW, Vickers AJ, Cook NR, Gerds T, Gonen M, Obuchowski N, et al. Assessing the performance of prediction models: a framework for some traditional and novel measures. Epidemiology. 2010 Jan;21(1):128–38.
70. Gerds TA, Cai T, Schumacher M. The performance of risk prediction models. Biom J. 2008 Aug;50(4):457–79.
71. Genolini C, Alacoque X, Sentenac M, Arnaud C. kml and kml3d: R Packages to

For peer review only

Cluster Longitudinal Data. Journal of Statistical Software. 2015 Jun 1;65(1):1–34.
72. Donado C, Friedrich Y, Kossowsky J, Locher C, Koechlin H. Exposure to Parental Depressive Symptoms: A Longitudinal Analysis on the Association with Adolescents' Depressive Symptoms and Adjustment Problems. J Dev Behav Pediatr. 2020 Sep;41(7):522–33.
73. Eadeh HM, Breaux R, Nikolas MA. A Meta-Analytic Review of Emotion Regulation Focused Psychosocial Interventions for Adolescents. Clin Child Fam Psychol Rev. 2021 Dec 1;24(4):684–706.
74. Nook EC, Satpute AB, Ochsner KN. Emotion Naming Impedes Both Cognitive Reappraisal and Mindful Acceptance Strategies of Emotion Regulation. Affec Sci [Internet]. 2021 Apr 20 [cited 2021 Apr 21]; Available from: https://doi.org/10.1007/s42761-021-00036-y
75. Vervoort T, Karos K, Johnson D, Sütterlin S, Van Ryckeghem D. Parental emotion and pain control behaviour when faced with child's pain : the emotion regulatory role of parental pain-related attention-set shifting and heart rate variability. PAIN [Internet]. 2018 [cited 2019 Apr 1]; Available from: http://hdl.handle.net/1854/LU-8573188
76. Allen LB, Tsao JCI, Seidman LC, Ehrenreich-May J, Zeltzer LK. A Unified, Transdiagnostic Treatment for Adolescents With Chronic Pain and Comorbid Anxiety and Depression. Cognitive and Behavioral Practice. 2012 Feb;19(1):56–67.
77. Cousins LA, Kalapurakkel S, Cohen LL, Simons LE. Topical Review: Resilience Resources and Mechanisms in Pediatric Chronic Pain. Journal of Pediatric Psychology. 2015 Oct 1;40(9):840–5.
78. Goubert L, Trompetter H. Towards a science and practice of resilience in the face of pain. European Journal of Pain. 2017;21(8):1301–15.
79. Hassett AL, Hilliard PE, Goesling J, Clauw DJ, Harte SE, Brummett CM. Reports of chronic pain in childhood and adolescence among patients at a tertiary care pain clinic. J Pain. 2013 Nov;14(11):1390–7.
80. Palermo TM. Pain prevention and management must begin in childhood: the key role of psychological interventions. PAIN. 2020 Sep;161:S114.
81. Walker LS, Dengler-Crish CM, Rippel S, Bruehl S. Functional abdominal pain in childhood and adolescence increases risk for chronic pain in adulthood. PAIN. 2010 Sep 1;150(3):568–72.
82. Tegethoff M, Belardi A, Stalujanis E, Meinlschmidt G. Comorbidity of Mental Disorders and Chronic Pain: Chronology of Onset in Adolescents of a National Representative Cohort. The Journal of Pain. 2015 Oct;16(10):1054–64.
83. Brennstuhl M, Tarquinio C, Montel S. Chronic pain and PTSD: Evolving views on their comorbidity. Perspectives in Psychiatric Care. 2015;51(4):295–304.
84. Jastrowski Mano KE, O'Bryan EM, Gibler RC, Beckmann E. The Co-occurrence of Pediatric Chronic Pain and Anxiety: A Theoretical Review of a Developmentally Informed Shared Vulnerability Model. Clin J Pain. 2019 Dec;35(12):989–1002.
85. Soltani S, Kopala-Sibley DC, Noel M. The Co-occurrence of Pediatric Chronic Pain and Depression: A Narrative Review and Conceptualization of Mutual Maintenance. Clin J Pain. 2019 Jul;35(7):633–43.
86. Soltani S, Noel M, Bernier E, Kopala-Sibley DC. Pain and insomnia as risk factors for first lifetime onsets of anxiety, depression, and suicidality in adolescence. PAIN. 2022 May 13;10.1097/j.pain.0000000000002879.

For peer review only

Authors' contributions

HK has designed the study, obtained funding, wrote the first draft of the manuscript, and acts as project leader and sponsor for the study described in this protocol.

JH has collaborated closely with HK in the process of detailing the study plan, has written and revised the manuscript, and is the PhD student in this project.

CCH, TD, and SC are the clinical collaborators of this study. They have all provided substantial input in the research plan and the study procedures, and have critically revised the manuscript.

JR has provided substantial input and feedback on the research plan, choice of outcome variables, and study procedures, and has critically revised the manuscript.

CL has provided substantial feedback on the design of the study and all study related procedures, and has critically revised the manuscript.

UH is responsible for data analysis in this project, has written the data analysis plan, provided advice on the power calculation, and has critically revised the manuscript.

All authors approved the final version of the manuscript, and agree to be accountable for all aspects of the work in ensuring that questions related to the accuracy or integrity of any part of the work are appropriately investigated and resolved.

Funding statement

This work is supported by a Swiss National Science Foundation grant awarded to HK: PZ00P1_208850 (Title: "Understanding and preventing chronic postsurgical pain in children")

Competing interest statement

The authors report no conflict of interest.

For peer review only

Figure 1. Overview of Assessment Time Points

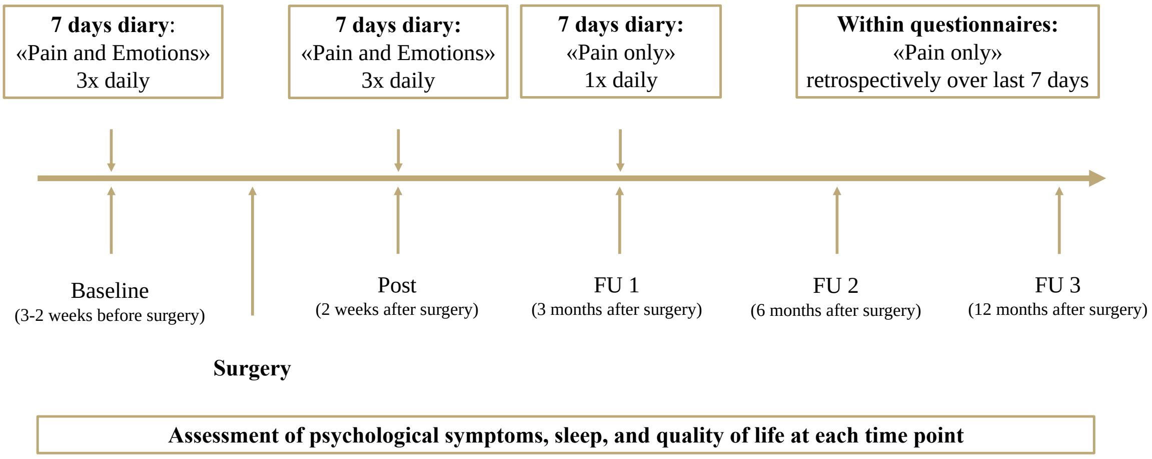

peer review only

BMJ Open

Longitudinal Resilience and Risk Factors in Pediatric Postoperative Pain (LORRIS): Protocol for a Prospective Longitudinal Hospital-Based Study

Journal:	*BMJ Open*
Manuscript ID	bmjopen-2023-080174.R1
Article Type:	Protocol
Date Submitted by the Author:	12-Feb-2024

Complete List of Authors:	Hochreuter, Jana; University Children's Hospital Zurich, Department of Psychosomatics and Psychiatry; University of Zurich, Department of Psychology<br>Dreher, Thomas ; University Children's Hospital Zürich, Department of Pediatric Orthopedics and Traumatology; Balgrist University Hospital, Department of Pediatric Orthopedics<br>Hasler, Carol-Claudius; Children’s Hospital Basel, Department of Orthopedics<br>Canonica, Sandro; University Children's Hospital Zürich, Pediatric Orthopedics and Traumatology; University Hospital Balgrist Zürich, Department of Pediatric Orthopedics<br>Locher, Cosima; University Hospital Zurich, Department of Consultation-Liaison Psychiatry and Psychosomatic Medicine; Plymouth University<br>Held, Ulrike; Epidemiology, Biostatistics and Prevention Institute, Biostatistics<br>Rabbitts, Jennifer; Stanford University, Anesthesiology Pediatric Anesthesiology<br>Koechlin, Helen; University Children's Hospital Zürich, Psychosomatics and Psychiatry; University of Zurich, Department of Psychology

**Primary Subject Heading**:	Paediatrics
Secondary Subject Heading:	Surgery
Keywords:	SURGERY, Adolescents < Adolescent, Chronic Pain, Risk Factors

SCHOLARONE™
Manuscripts

Longitudinal Resilience and Risk Factors in Pediatric Postoperative Pain (LORRIS): Protocol for a Prospective Longitudinal Hospital-Based Study

Hochreuter, J.[1,2,3], Dreher, T.[3,4,5], Hasler, C.-C.[6], Canonica, S.[3,4,5], Locher, C.[7,8], Held, U.[9], Rabbitts, J.A.[10], & Koechlin, H.[1,2,3]

[1]Department of Psychosomatics and Psychiatry, University Children's Hospital, University of Zurich, Zurich, Switzerland.

[2]Division of Child and Adolescent Health Psychology, Department of Psychology, University of Zurich, Zurich, Switzerland.

[3]Children's Research Centre, University Children's Hospital Zurich, University of Zurich, Zurich, Switzerland.

[4]Department of Pediatric Orthopedics and Traumatology, University Children's Hospital Zurich, Switzerland.

[5]Department of Pediatric Orthopedics, Orthopedic University Hospital Balgrist, Zurich, Switzerland

[6]Department of Orthopedics, Children's Hospital Basel, Switzerland.

[7]Department of Consultation-Liaison Psychiatry and Psychosomatic Medicine, University Hospital Zurich, Zurich, Switzerland.

[8]Faculty of Health, University of Plymouth, UK.

[9]Department of Biostatistics and Epidemiology, Biostatistics and Prevention Institute, University of Zurich, Switzerland.

[10]Anesthesiology, Perioperative and Pain Medicine, Pediatric Anesthesiology, Stanford University, USA.

Corresponding author: Helen Koechlin, PhD, University Children's Hospital Zurich, Bergstrasse 57, 8032 Zurich, Switzerland. Email address: helen.koechlin@kispi.uzh.ch

For peer review only - http://bmjopen.bmj.com/site/about/guidelines.xhtml

Abstract

Introduction

Chronic postsurgical pain (CPSP) is defined as pain that persists after a surgical procedure, and has significant impact on quality of life. Previous studies show the importance of psychological factors in CPSP, yet the majority of studies focused solely on negative emotions. This longitudinal observational study aims to broaden this knowledge base by examining the role of emotional state, emotion variability, emotion regulation, and emotion differentiation on the child- and the parent-level for the development CPSP, and to describe pain and emotion-related trajectories following surgery.

Methods and analysis

We intend to include 280 children and adolescents aged 8-18 years with a planned orthopedic surgery and their parents. A total of five assessment time points is planned: 3 weeks before surgery (baseline), 2 weeks after surgery (post), and 3 months (Follow-up [FU] 1), 6 months and 12 months after surgery. At baseline and post only, children and parents are asked to complete a daily diary thrice a day for a week where they rate their current emotional state and their pain severity (children only). Emotional state ratings will be used to calculate indices of emotion variability, emotion regulation, and emotion differentiation. Children and parents will complete questionnaires at each time point, including measures on quality of life, social support, sleep, and symptoms of anxiety and depression.

To predict development of CPSP, generalized linear regression models will be used, resulting in odds ratios and 95% confidence intervals. Pearson product-moment correlations between predictors and outcomes will be evaluated at each time point. The primary outcome of the prediction model is CPSP at FU1. For the trajectory analysis, the classification method K-means for longitudinal data will be used to determine clusters in the data.

Ethics and dissemination

The Ethics Committee of the Canton of Zurich, Switzerland, has approved the study (ID: 2023-01475). Participants will be compensated, and a dissemination workshop will be held.

Registration details

Clinicaltrials.gov: NCT05816174

For peer review only - http://bmjopen.bmj.com/site/about/guidelines.xhtml

Strengths and limitations of this study

- This is a prospective, multicenter, longitudinal hospital-based study and the sample size allows to examine a range of risk and resilience factors.
- The longitudinal study design permits measurement of pain- and emotion-related trajectories up until 12 months after surgery.
- The repeated assessments design will lead to an in-depth understanding of changes of risk and resilience factors over time.
- The primary limitation of this study is the potential loss to follow-up and missing datapoints due to several points of measurement.
- The results will be mainly based on self-reports, which are considered gold standard but can be subject to bias.

For peer review only

Introduction

Chronic postsurgical pain (CPSP) is defined as chronic pain that persists over time (at least 3 months) following a surgical procedure, and that has significant impact on physical and psychosocial quality of life (1,2). Meta-analytic estimates in children and adolescents describe that around 20% of pediatric patients undergoing major surgery report chronic pain three to twelve months after surgery (2). Highest pain intensity ratings are observed in two-weeks following surgery, with a steady decrease afterwards over the following six months (3), and pain ratings two weeks after surgery have been shown to predict the development of moderate to severe pain one year later (4,5). Further, most children and adolescents experience decreases in their physical activity levels following surgery, and it has been suggested that objectively monitored physical activity levels could be useful to indirectly assess functional recovery (6). A recent study in adolescents undergoing spinal fusion found that a majority of participants were characterized by a steep decrease of physical activity after surgery (7).

Previous research has identified psychosocial risk factors that are associated with the development of CPSP, among them presurgical pain intensity, child anxiety, child pain coping efficacy, child sleep patterns, and parental pain catastrophizing (2,8,9). Generally, emotions and emotion-related factors are known to influence pain, pain-related disability, and pain-related distress (10,11): There is some research on the role of emotion regulation in chronic pain (12), and the role of *negative* emotions is fairly well-documented in the pain experience (10,13,14). However, other emotion-related factors such as emotion differentiation and emotion variability have yet to be studied in the context of pediatric CPSP, as they present as good targets for prevention and treatment modules and can be screened for in clinical practice.

Emotion variability

Emotion variability describes the range of emotional fluctuations around an individual's average emotional intensity (15,16). Greater emotion variability has been linked to more mental health symptoms in adolescents (17,18), and higher emotion regulation demands (19). In pediatric chronic pain, one study found greater positive emotion variability to be associated with less pain-related interference and more engagement in activities despite pain (16). Emotion variability can be measured using the within-person standard deviation of repeated assessments of current emotional state (16,18,20,21).

Emotion differentiation

Emotion differentiation defines the level of specificity people use when identifying their emotional experiences (22–24). Higher emotion differentiation indicates greater ability to differentiate between one's own emotional states on a more fine-grained level and by using

For peer review only - http://bmjopen.bmj.com/site/about/guidelines.xhtml

precise terms (24). High or low emotion differentiation can be calculated by means of intraclass correlations, where high consistency in emotion ratings across a predefined episode (e.g., a week-long daily diary phase) suggests poorer differentiation (24,25). Previous research has demonstrated that higher emotion differentiation is associated with better mental health (see (26) for a meta-analysis). To date, emotion differentiation has not been studied in the context of pediatric chronic pain.

Emotion regulation

Emotion regulation refers to a person's ability to influence what emotions they have, when they have them, and how they experience and express these emotions (27,28). A handful of previous studies have explored the role of emotion regulation in pediatric chronic pain (e.g., (16,20,29,30), yielding mixed results: While some found that different types of emotion regulation, namely expressive suppression and cognitive reappraisal, were associated with more frequent hospitalization due to pain (29), and lower engagement in activities despite the pain (16), others did not identify significant associations between emotion regulation and pain intensity or pain-related disability (20,30). The habitual use of specific emotion regulation strategies is considered stable within individuals (31–33), and can be measured by means of questionnaires (19).

Familial influences on pediatric pain

Previous research has shown that around 50% of children and adolescents with chronic pain have a parent who has had chronic pain in the past or is currently living with it (34). Parents and the family environment have been identified as a "key context" (p. 702) regarding the understanding, assessment, and management of pediatric pain (35), and a bidirectional relationship between child functioning and parent factors is suggested (35–37). Regarding emotion-related factors, the family context is considered crucial: Children learn the appropriateness of valence, duration, and intensity of emotions and emotion expression by observing their parents and parents' emotion regulation behaviors (38,39).

In sum, previous studies have identified several risk factors for the development of CPSP, among them symptoms of anxiety, preoperative pain intensity, and poorer sleep quality as well as parental catastrophizing, even though the results were equivocal in part (5,6,40). The extent to which emotion-related factors can serve as *resilience* factors has had minimal investigation in the transition from acute to chronic postsurgical pain (5). Further, the promotion of resilience mechanisms as a prevention strategy has had limited focus in the context of pediatric chronic pain (7,41).

Objectives

For peer review only - http://bmjopen.bmj.com/site/about/guidelines.xhtml

The aim of this observational longitudinal study is to specifically examine the potential role of emotion-related factors (namely emotion variability, emotion differentiation, and emotion regulation), as well as familial influences in the development of CPSP and describe pain- and emotion-related trajectories following major surgery up to 12 months after surgery. We aim to identify whether and which emotion-related factors increase or decrease the risk for the development of CPSP after planned orthopedic surgery. To the best of our knowledge, this is the first study to specifically examine these emotion-related factors as both potential risk *and* resilience factors in the development of CPSP.

For peer review only

Methods and Analysis

Study design and setting

This is a prospective longitudinal observational study in patients undergoing scheduled orthopedic surgery and their parents. The study starts in January 2024 and will continue to run through the end of January 2027. As scheduled orthopedic surgeries are planned well in advance, patients can be approached early and asked about their interest in participating in the study. See Figure 1 for an overview of assessment time points. The study is registered at clinicaltrials.gov (NCT05816174). All participants will be recruited from three University Children's Hospitals in Switzerland.

Patient and Public Involvement

Patients were not involved in the design of the study, however, the first 10 families that complete the post assessment will be interviewed and asked about their experience in the study so far. This feedback will be used to adapt study procedures if needed. The results will be disseminated to study participants via a newsletter and a dissemination event where participants, clinical collaborators, and the interested public will be invited to discuss the study results.

Sample and Sample Size Calculation

The anticipated prevalence rate of CPSP is 25%, based on international estimates (2,42). The aim of this study is to recruit a sample of 280 child-parent dyads, resulting in an anticipated 70 children and adolescents with CPSP. In a power calculation for a logistic regression model with 10 events per variable, this allows to examine 7 predictor variables with CPSP status as the primary outcome. Assumptions regarding events per variable will be re-evaluated following recently proposed recommendations (43,44).

For peer review only - http://bmjopen.bmj.com/site/about/guidelines.xhtml

Eligibility criteria

We will include participants between 8 and 18 years who have a planned in-patient orthopedic surgery at one of the study centers. The child-parent dyads must be able to read (for younger children: with help) and understand German. Exclusion criteria will be serious comorbid health condition (e.g., cancer, severe neurological impairment, chronic illness requiring daily medication), and prior major surgery (e.g., prior spine surgery). Mental health comorbidities are no reason for exclusion.

Study Design

This is a prospective longitudinal observational study in patients undergoing scheduled orthopedic surgery and their parents. As scheduled orthopedic surgeries are planned well in advance, patients can be approached early and asked about their interest in participating in the study. See Figure 1 for an overview of assessment time points. The study is registered at clinicaltrials.gov (NCT05816174).

Procedures

Potential participants will be informed about the study by the clinical collaborators at each study site during one of their pre-surgical visits (e.g., anesthesia preparation interview, scheduling visit). Based on the clinical routine of the study centers, the procedures might differ slightly. If patients fall within the required age range, the clinical collaborators will provide the family with a leaflet with the most important study information. On the leaflet, a QR-Code brings families directly to a site on the website of the University Children's Hospital Zurich (main study center), where they can find the most important information again. If families express their initial interest about the study, the physician will take a note and inform the study team, which will then contact the family. A member of the study team will then approach the respective family at their next visit at the hospital or via phone call and go through the informed consent / assent procedure with them. In all cases, parents sign the consent form for their own participation. In children under the age of 14 years, parents also sign the consent form for their child's participation, while adolescents aged 14 years or more sign their own consent form. This is in line with the Human Research Act (45), that states that adolescents aged 14 years and more who are capable of judgement and who are to participate in a study with minimal risks can sign their own consent form. In participating hospitals, patients typically have (at least) two visits before the surgery. Once the written informed consent and assent is given, families will complete a battery of questionnaires as baseline measures before the surgery via direct link to the online questionnaires (see next section and Table 1 for an overview; see Supplement for an overview of reliability, validity, benefits, and

For peer review only

limitations of all questionnaires), using Research Electronic Data Capture (RedCap; (46)). In addition, participants will be asked to download the smartphone application SEMA 3 (47), a free tool for ecological momentary assessment provided by the University of Melbourne, Australia, especially developed for research needs.

Families will be called again two weeks after the surgery, and asked to complete the post-assessment via direct link to the questionnaires. Families will be contacted again 3 months (FU 1), 6 months (FU 2), and 12 months (FU 3) after surgery to complete the follow-up surveys, and asked to complete the surveys at each assessment point within one week after receiving the link to the survey; up to three weekly reminders will be sent after the initial message.

In addition to the questionnaires at each assessment point, all participants (i.e., children and parents) will complete daily diaries: To estimate the influence of emotion-related factors on pain trajectories, children and adolescents will complete two daily diary phases (i.e., two weeks of daily diary) during which they rate both their pain severity and emotional state (referred to as "pain and emotions" throughout the protocol; at baseline and post assessment), and one daily diary episode during which they only rate their pain intensity (referred to as "pain only" throughout the protocol; at Follow-up 1. See Figure 1). Parents will complete two daily diary phases (at baseline and post) during which they exclusively rate their emotional state.

Daily diary - pain and emotions (two timepoints): At baseline and post assessment, children and adolescents will complete ratings of their pain severity on an 11-point numeric rating scale with anchors 0 = "no pain" and 10 = "worst pain possible". Pain severity includes pain intensity, pain-related distress, and pain-related interference; hence, participants will give three different ratings (41,42). The pain ratings will be used to classify the presence of acute postsurgical pain at the post assessment (in combination with a health-related quality of life rating; see overview of questionnaires for this time point in Table 1). In addition, all participants will rate the extent to which they experience positive and negative emotions three times each day on their smartphone and during their normal daily routine over the course of 7 days. These ratings will be used to calculate a score of emotion differentiation (i.e., the ability to differentiate between emotional states), a mean value of positive and negative emotions, and an index of emotional variability (26,36,37; see below for more detailed description). The items of the short version of the Positive and Negative Affect Schedule for Children (PANAS-C) will be used to assess emotional state (38–40).

For peer review only - http://bmjopen.bmj.com/site/about/guidelines.xhtml

<u>Daily diary - pain only (once)</u>: To diagnose CPSP, participants will be asked to rate their daily pain severity for 7 days starting at FU1. Participants will be prompted at the end of each day to respond on an 11-point numeric rating scale with anchors 0 = "no pain" and 10 = "worst pain possible". In addition, daily medication use will be indicated (Yes/No and listing medication in a free text field).

Number of steps taken each day will be counted as an indicator of changes in physical activity before and after surgery (i.e., at the post assessment), measured by means of accelerometer (GENEActive, Activeinsights Ltd), an ambulatory, non-invasive activity-monitoring device that registers physical activity, and can be worn around the wrist.

Study Measures

See Table 1 for an overview, see Supplement for an overview of reliability, validity, benefits, and limitations of all questionnaires.. For all measures, we use the validated German version (if available) or translate and back-translate the items with the help of a professional interpreter or native speaker. Measures of primary interest are marked with an * in Table 1, all other variables are considered exploratory.

<u>Child Measures</u>

Emotional state: The Positive and Negative Affect Schedule for Children (PANAS-C) (48–50) is a measures of positive and negative affect by providing mood adjectives (e.g. "scared", "happy"). Participants are asked to rate 10 items on a 5-point Likert-scale from 1 (= very slightly or not at all) to 5 (= very much) to assess the extent to which they currently feel each of 5 positively and 5 negatively valenced emotions.

Pain Screening Tool: The Pediatric Pain Screening Tool (PPST) (51,52) entails 9 self-reported items that belong to a physical and psychosocial subscale. Answers are given dichotomously (disagree = 0, agree = 1). Higher scores indicate higher risk status of poor pain-related outcomes.

Fear of Pain: The Fear of Pain Questionnaire for Children (FOPQ-C) (53) is a 24-item self-report inventory to assess fear of pain. Participants are asked to rate each item on a Likert-scale from 0 (= strongly disagree) to 4 (= strongly agree). A higher total score indicates higher fear.

For peer review only

Functional Disability: Children's self-reported difficulty in physical and psychosocial functioning due to their physical health is assessed by the Functional Disability Inventory (FDI) (54). 15 items are rated on a Likert-scale from 0 (= no problems) to 4 (= not possible) and concern perceptions of activity limitations during the past 2 weeks. Higher scores indicate greater disability.

Security in Parent-Child Relationships: The Security Scale (55) consists of 15 items and assesses participants' perceptions of security in parent-child relationships. Items are presented in a format of statements, participants choose which statement is more characteristic of them and then rate the statement on a Likert-scale from 1 (= really true) to 4 (= sort of true). Higher scores indicate a more secure attachment.

Symptoms of Anxiety and Depression: The RCADS-short version (56) is a 25-item self-report questionnaire used to assess symptoms of depression and anxiety. Participants are asked to indicate how often each item applies to them according to a 4-point Likert-scale from 0 (= never) to 3 (= always). High scores indicate high anxiety and depression symptoms.

Sleep: The Pittsburgh Sleep Quality Index (PSQI) (57) is the most commonly used retrospective self-report questionnaire that measures sleep quality over the previous month. We will use a version that has been slightly adapted for children by researchers of the University Children's Hospital. This version contains of 25 items, and each item is rated on a scale from 0 (= no difficulty) to 3 (= severe difficulty). In addition to a global sleep quality factor, seven domains of sleep difficulties can be assessed: Sleep quality, sleep latency, sleep duration, habitual sleep efficiency, sleep disturbances, use of sleeping medications, and daytime dysfunction. A higher global score indicates more sleep difficulties.

Sensory Processing Sensitivity: The Highly Sensitive Child Scale (HSC) (58) is a 12-item self-reported questionnaire. Participants are asked to rate each item on a Likert-scale from 1 (= not at all) to 7 (= extremely). In addition to a total score, three subscales can be calculated: Low Sensory Threshold (LST; 3 items, e.g., "I don't like watching TV programs that have a lot of violence in them"), Ease of Excitation (EOE; 5 items, e.g., "I get nervous when I have to do a lot in little time"), and Aesthetic Sensitivity (AES; 4 items, e.g., "I love nice smells"). Higher scores indicate higher sensitivity.

For peer review only - http://bmjopen.bmj.com/site/about/guidelines.xhtml

Emotion Regulation: The Emotion Regulation Questionnaire for Children and Adolescents (ERQ-CA) (19) is a self-report questionnaire containing 10 items. Items are rated on a Likert-scale from 1 (= strongly disagree) to 7 (= strongly agree), and assess individual differences in the habitual use of two emotion regulation strategies, namely Cognitive Reappraisal (e.g., "When I'm faced with a stressful situation, I make myself think about it in a way that helps me stay calm") and Expressive Suppression (e.g., "I keep my emotions to myself"). Higher scores indicate more habitual use of the respective emotion regulation strategy.

Quality of Life: The Pediatric Quality of Life Inventory (PedsQL) (59) is a self-or parent-reported questionnaire for children and adolescents. 23 Items are rated on a Likert-scale from 0 (= never a problem) to 4 (= almost always a problem). The questionnaire consists of the subscales physical functioning (e.g., "It is hard for me to run"), emotional functioning (e.g., "I feel sad or blue"), social functioning (e.g., "It is hard to keep up with my peers"), and school functioning (e.g., "It is hard to pay attention in class") in the past month. Higher scores indicate better quality of life.

Social Support: The Social Support Questionnaire for Children (SSQC) (60) consists of 50 items. It measures children's social support across five distinct sources of support: parents, relatives, nonrelative adults, siblings, and peers. Items are rated on a 4-point Likert-scale ranging from 0 (= never or rarely true) to 3 (= often or always true). Higher scores indicate higher levels of perceived support.

Symptoms of Posttraumatic Stress: The Child and Adolescent Trauma Screen 2 (CATS-2) (61) contains a checklist of 15 potentially traumatic events (PTEs), followed by an item asking which of the PTEs bothers them most. Posttraumatic stress symptoms are measured by 20 items that are rated on a 4-point Likert-scale ranging from 0 (= never) to 3 (= almost always). Five yes/no items assess whether the previously rated posttraumatic stress symptoms interfere with five key areas of functioning and are used to assess psychosocial functioning.

Parent Measures

Postoperative Pain: With the Postoperative Pain Measure for Parents (PPMP) (62), parents can assess and estimate their child's postoperative pain. The checklist includes 15 items where the parents select "yes" (= 1 point) or "no" (= 0 points) as to whether the child exhibits pain-related behavior, with higher scores indicating more postoperative pain.

The Postoperative Pain Measure for Parents (PPMP) [illegible]

For peer review only - http://bmjopen.bmj.com/site/about/guidelines.xhtml

Fear of Pain Questionnaire: The Parent Fear of Pain Questionnaire (FOPQ-P) (35) is a 23-item self-report inventory to reflect a parent's own fear associated with their child's pain experience. Parents are asked to rate each item on a Likert-scale from 0 (= strongly disagree) to 4 (= strongly agree). A higher total score indicates higher fear of pain.

Symptoms of Anxiety and Depression: The Hospital Anxiety and Depression Scale (HADS) (63) is a widely used measure of psychological distress, and indicates the presence of symptoms of depression (seven items) and anxiety (seven items) over a 1-week period. The 14 items are rated on a four-point Likert-scale ranging from 0 to 3, with higher scores indicate greater symptom severity.

Sensory Processing Sensitivity: The Highly Sensitive Person Scale (HSP) (64,65) is a 12-item self-reported questionnaire, and participants are asked to rate each item on a Likert-scale from 1 (= strongly disagree) to 7 (= strongly agree). In addition to a total score, three subscales can be calculated, as for the HSC Scale: Low Sensory Threshold, Ease of Excitation, and Aesthetic Sensitivity. Higher scores reflect higher sensitivity.

Emotion Regulation: The Emotion Regulation Questionnaire (ERQ) (31) assesses the habitual use of two emotion regulation strategies, namely Cognitive Reappraisal (6 items) and Expressive Suppression (4 items). The 10 self-reported items are rated on a 7-point Likert-scale ranging from 1 (= strongly disagree) to 7 (= strongly agree). Higher scores indicate higher usage of the respective strategy.

Well-Being: Parental well-being will be measured using he WHO-5 index (66). It consists of 5 items that are rated on a 6-point Likert-scale ranging from 0 (= never) to 5 (=always), indicating how often participants have felt in “good spirits”, “active”, “relaxed”, etc. over the course of the last 2 weeks. Higher scores indicate more well-being.

Table 1. Measures, Tools, Domain and Time-Points for Data Collection

**Domain**		**Measure**	**Completed by**	**BL**	**Post**	**FU 1**	**FU 2**	**FU 3**
**Demographics**	Age, gender, school, school attendance and performance; family	Parent and self-report: online baseline questionnaire	Parent(s) and child	X				

For peer review only - http://bmjopen.bmj.com/site/about/guidelines.xhtml

	constellation, parental educational level and work status							
**Medical history**	Neonatal intensive care (yes/no), previous surgeries (yes/no; which one), child's pain history (pain duration, pain location)	Parent report: online baseline questionnaire	Parents	X				
	Complications in the hospital (yes/no) Pain treatment immediately following surgery	Child's medication list in electronic health record	Data entered by study team		X			
**Experience-sampling method**	Positive and negative emotions (emotion variability and emotion differentiation)*	Items from the Positive and Negative Affect Schedule (48)	Daily diary: child and parent	X	X			
	Pain severity	Numeric Rating Scale (NRS), 0-10	Daily diary: child	X	X	X		
**Psychosocial factors child**								
	Pain severity (intensity, distress, interference (67))*	Numeric Rating Scale (NRS), 0-10	Child self-report				X	X
	Preoperative screening	Pediatric Pain Screening Tool (51,52)	Child self-report	X				
	Functional Disability	Functional Disability Index (FDI; (54))	Child self-report	X	X	X	X	X
	Fear of Pain	Fear of Pain Questionnaire (53)	Child self-report	X				
	Pain catastrophizing	Pain Catastrophizing scale – 3 item version (68)	Child self-report	X				
	Attachment; parent–child relationship	Security Scale (69)	Child self-report	X				
	Symptoms of anxiety and depression*	Revised Child Anxiety and Depression Scale-short version (56)	Child self-report	X	X	X	X	X
	Sleep	Pittsburgh Sleep Quality Index (PSQI; (57))	Child self-report	X	X	X	X	X
	Sensory processing sensitivity	Highly Sensitive Child Scale (58)	Child self-report	X				
	Emotion regulation*	Emotion Regulation Questionnaire – Child and Adolescent version (19)	Child self-report	X				
	Quality of life	Pediatric Quality of Life Inventory (59)	Child self-report	X	X	X	X	X
	Social support	Social Support Questionnaire for Children (60)	Child self-report	X				

For peer review only - http://bmjopen.bmj.com/site/about/guidelines.xhtml

	Posttraumatic stress symptoms	Child and Adolescent Trauma Screening (CATS) (70)	Child self-report	X		X		
**Psychosocial factors parents**								
	Parents' own pain history	2 questions: "(1) Have you ever and (2) are you currently living with persistent or recurrent pain for at least 3 months?"	Parent self-report	X				
	Postoperative pain measure (child's pain)	Postoperative Pain Measure for Parents (62)	Parent report		X			
	Fear of pain	Parent Fear of Pain Questionnaire (35)	Parent self-report	X				
	Pain catastrophizing	Pain Catastrophizing Scale – 3 item version (68)	Parent self-report	X				
	Anxiety and depression*	Anxiety and Depression Scale (63)	Parent self-report	X	X	X	X	X
	Sensory processing sensitivity	Highly Sensitive Person Scale (65)	Parent self-report	X				
	Emotion regulation*	Emotion Regulation Questionnaire (31)	Parent self-report	X				
	Quality of life	World Health Organization Well-Being Index (66)	Parent self-report	X	X	X	X	X
**Primary outcome**								
	Chronic postsurgical pain	Presence of CPSP: pain diary for 7 consecutive days (NRS) + health-related quality of life (PedsQL, see above)				X		

Note. Primary predictors are marked with an *. Unmarked variables are considered secondary or exploratory.

Primary Outcome

The primary endpoint is CPSP at FU 1. CPSP is defined as greater than minimal pain (pain intensity ≥ 3) on more than 50% of days as measured by daily diary over 7 days, and impairment in health-related quality of life (score of ≤ 74.9 on the *Pediatric Quality of Life Inventory*, PedsQL (59), i.e., 1 SD below the population mean based on normative US data). The combination of pain and quality of life measures will result in a binary variable (CPSP yes/no). This measurement of CPSP is supported by previous research (6,71,72), and a modified definition of CPSP by the International Association for the Study of Pain (IASP) (73).

For peer review only - http://bmjopen.bmj.com/site/about/guidelines.xhtml

Secondary Outcomes

Secondary endpoints are trajectories of pain and emotion-related factors over time (i.e., baseline through FU3), number of steps taken by patients, pain severity, and quality of life. Postoperative complications will be monitored using the modified Clavien-Dindo Classification (74).

Statistical methods

Descriptive statistics will include mean and standard deviation or median and inter quartile range for normally distributed and non-normally distributed or ordinal variables measured at baseline. Categorical variables will be reported as number and percentage of total. The primary outcome is binary, therefore generalized linear regression models or generalized linear mixed effects models will be used, using a logit link function. Fixed effects will be estimated for the primary predictors (as marked in Table 1). Random effects will be included in the longitudinal model to account for repeated observations in the individual participants. The resulting estimands will be odds ratios and 95% confidence intervals. Variables at baseline will be used in the prediction model, with performance measures area under the curve (AUC), calibration, and scaled Brier Score (75,76). Variables measured consecutively over multiple days at each assessment interval will be averaged over time. We will use the rule of 10 events per variable (EPV) that would determine the number of independent variables in the regression model. Potentially time-varying covariates will be addressed. Potentially non-linear relationships of the independent variables with outcome will be evaluated. Further, the purpose of the multivariable regression analysis is to develop a risk score for CPSP. This will allow to identify variables that increase or decrease the risk for CPSP, by combining them into a multivariable model.

The pre-selected variables (i.e., the primary predictors) will be assessed for collinearity with pair-wise scatter plots, pair-wise interaction terms will be included successively into the model. For the pre-selected variables, no variable selection will be performed. In an exploratory analysis, potential other variables will be evaluated and the process will be described in more detail in the statistical analysis plan.

Any missing values in the independent variables or the outcome will be addressed with 100-fold multiple imputation, using chained equations. Resulting estimates will be pooled using Rubin's rule (77,78).

We will conduct bivariate analyses to compare study completers versus study dropouts to detect potential differences regarding participant demographic between the groups.

For peer review only - http://bmjopen.bmj.com/site/about/guidelines.xhtml

Baseline daily pain ratings will be averaged for each participant to calculate a mean baseline pain intensity score. Post assessment and FU 1 daily pain ratings will be combined with health-related quality of life data to define binary outcome variables for acute postsurgical pain and CPSP. Acute postsurgical pain and CPSP will be defined independently.

For the trajectory analysis, the classification method K-means for longitudinal data will be used to determine clusters in the data (R package kml) (79). The analysis of clusters joint trajectories (R package kml3d) (79) will be applied, as this analysis allows summarizing several correlated continuous variables into a single nominal variable (80). The trajectory analyses will be considered exploratory. Alternative clustering methods would be based on the risk score and participants with high risk for developing CPSP can be identified based on a cut-off of 10% predicted probability.

A detailed statistical analysis plan will be written up and finalized before data export for analysis. All analyses will be performed with the R programming language in a fully scripted way and dynamic reporting will be used to guarantee highest level of reproducibility.

Monitoring

The study will be monitored for quality and regulatory adherence. The monitor, who is not involved in our study and supervised by the Clinical Trials Center of the University Hospital Zurich, Switzerland, will ensure that the specifications of the project plan and the associated processes are adhered to.

Ethics and Dissemination

The Ethics Committee of the Canton of Zurich, Switzerland, has approved the study (ID: 2023-01475). All participants will be compensated for their time by means of a voucher. We will reimburse participating families per assessment time point, so that families who drop out will still receive a certain amount of compensation. The first few participating families will be asked about general accessibility of the study. Their feedback will be implemented in study procedures if necessary.

The risk of adverse effects due to the study are considered to be minimal. All project data will be handled with uttermost discretion and will only be accessible to the study team. Data will be stored on a secured and password-protected server.

For peer review only - http://bmjopen.bmj.com/site/about/guidelines.xhtml

This study is registered at clinicaltrials.gov (identification number: NCT05816174). After study completion, the study team organizes a dissemination workshop aiming at youth with CPSP and their parents, researchers, clinicians, and the interested public, to discuss study results, clinical implications, and formulate future research questions.

For peer review only

Full references

1. Schug SA, Lavand'homme P, Barke A, Korwisi B, Rief W, Treede RD, et al. The IASP classification of chronic pain for ICD-11: chronic postsurgical or posttraumatic pain. Pain. 2019 Jan;160(1):45–52.
2. Rabbitts JA, Fisher E, Rosenbloom BN, Palermo TM. Prevalence and predictors of chronic postsurgical pain in children: A systematic review and meta-analysis. J Pain. 2017 Jun;18(6):605–14.
3. Connelly M, Fulmer RD, Prohaska J, Anson L, Dryer L, Thomas V, et al. Predictors of Postoperative Pain Trajectories in Adolescent Idiopathic Scoliosis. Spine. 2014 Feb 1;39(3):E174.
4. Pagé MG, Stinson J, Campbell F, Isaac L, Katz J. Identification of pain-related psychological risk factors for the development and maintenance of pediatric chronic postsurgical pain. J Pain Res. 2013 Mar 5;6:167–80.
5. Rosenbloom BN, Katz J. Modeling the transition from acute to chronic postsurgical pain in youth: A narrative review of epidemiologic, perioperative, and psychosocial factors. Canadian Journal of Pain. 2022 Jun 15;6(2):166–74.
6. Rabbitts JA, Zhou C, Groenewald CB, Durkin L, Palermo TM. Trajectories of postsurgical pain in children: risk factors and impact of late pain recovery on long-term health outcomes after major surgery. PAIN. 2015 Nov;156(11):2383–9.
7. Beeckman M, Hughes S, Van der Kaap-Deeder J, Plasschaert F, Michielsen J, Moens P, et al. Risk and Resilience Predictors of Recovery after Spinal Fusion Surgery in Adolescents. The Clinical Journal of Pain [Internet]. 2021 Aug 23 [cited 2021 Sep 8];Publish Ahead of Print. Available from: https://journals.lww.com/10.1097/AJP.0000000000000971
8. Katz J, Weinrib AZ, Clarke H. Chronic postsurgical pain: From risk factor identification to multidisciplinary management at the Toronto General Hospital Transitional Pain Service. Canadian Journal of Pain. 2019 Jul 15;3(2):49–58.
9. Rabbitts JA, Groenewald CB, Tai GG, Palermo TM. Presurgical psychosocial predictors of acute postsurgical pain and quality of life in children undergoing major surgery. J Pain. 2015 Mar;16(3):226–34.
10. Wiech K, Tracey I. The influence of negative emotions on pain: behavioral effects and neural mechanisms. Neuroimage. 2009 Sep;47(3):987–94.
11. Barke A, Koechlin H, Korwisi B, Locher C. Emotional distress: Specifying a neglected part of chronic pain. European Journal of Pain. 2020;24(3):477–80.
12. Koechlin H, Coakley R, Schechter N, Werner C, Kossowsky J. The role of emotion regulation in chronic pain: A systematic literature review. Journal of Psychosomatic Research. 2018 Apr 1;107:38–45.
13. Berna C, Leknes S, Holmes EA, Edwards RR, Goodwin GM, Tracey I. Induction of depressed mood disrupts emotion regulation neurocircuitry and enhances pain unpleasantness. Biological psychiatry. 2010 Jun 1;67(11):1083–90.
14. Janssen SA. Negative affect and sensitization to pain. Scand J Psychol. 2002 Apr;43(2):131–7.
15. Reitsema AM, Jeronimus BF, Dijk M van, Jonge P de. Emotion dynamics in children and adolescents: A meta-analytic and descriptive review. Emotion [Internet]. 2021 Jan [cited 2021 Apr 19]; Available from: https://research.rug.nl/en/publications/emotion-dynamics-in-children-and-adolescents-a-meta-analytic-and-
16. Koechlin H, Beeckman M, Meier AH, Locher C, Goubert L, Kossowsky J, et al. Association of parental and adolescent emotion-related factors with adolescent chronic pain behaviors. PAIN [Internet]. 2022 [cited 2021 Oct 28]; Available from: https://journals.lww.com/pain/Abstract/9000/Association_of_parental_and_adolescent.97860.aspx
17. Neumann A, van Lier PAC, Frijns T, Meeus W, Koot HM. Emotional Dynamics in the

For peer review only - http://bmjopen.bmj.com/site/about/guidelines.xhtml

Development of Early Adolescent Psychopathology: A One-Year Longitudinal Study. J Abnorm Child Psychol. 2011 Jul 1;39(5):657–69.
18. Silk JS, Steinberg L, Morris AS. Adolescents' Emotion Regulation in Daily Life: Links to Depressive Symptoms and Problem Behavior. Child Development. 2003;74(6):1869–80.
19. Gullone E, Taffe J. The Emotion Regulation Questionnaire for Children and Adolescents (ERQ-CA): a psychometric evaluation. Psychol Assess. 2012 Jun;24(2):409–17.
20. Connelly M, Bromberg MH, Anthony KK, Gil KM, Franks L, Schanberg LE. Emotion Regulation Predicts Pain and Functioning in Children With Juvenile Idiopathic Arthritis: An Electronic Diary Study. J Pediatr Psychol. 2012 Jan 1;37(1):43–52.
21. Kalokerinos EK, Murphy SC, Koval P, Bailen NH, Crombez G, Hollenstein T, et al. Neuroticism may not reflect emotional variability. Proceedings of the National Academy of Sciences. 2020 Apr 28;117(17):9270–6.
22. Barrett LF, Gross J, Christensen TC, Benvenuto M. Knowing what you're feeling and knowing what to do about it: Mapping the relation between emotion differentiation and emotion regulation. Cognition and Emotion. 2001 Nov 1;15(6):713–24.
23. Kashdan TB, Barrett LF, McKnight PE. Unpacking Emotion Differentiation: Transforming Unpleasant Experience by Perceiving Distinctions in Negativity. Curr Dir Psychol Sci. 2015 Feb 1;24(1):10–6.
24. Nook E. Emotion Differentiation and Youth Mental Health: Current Understanding and Open Questions [Internet]. PsyArXiv; 2021 [cited 2021 Apr 26]. Available from: https://psyarxiv.com/qkdbu/
25. Kalokerinos EK, Erbas Y, Ceulemans E, Kuppens P. Differentiate to Regulate: Low Negative Emotion Differentiation Is Associated With Ineffective Use but Not Selection of Emotion-Regulation Strategies. Psychol Sci. 2019 Jun;30(6):863–79.
26. Seah THS, Coifman KG. Emotion differentiation and behavioral dysregulation in clinical and nonclinical samples: A meta-analysis. Emotion. 2021;No Pagination Specified-No Pagination Specified.
27. Gross JJ. Emotion Regulation. In: Handbook of emotions. 3rd ed. New York: The Guilford Press; 2008.
28. Gross JJ. Emotion Regulation: Current Status and Future Prospects. Psychological Inquiry. 2015 Jan 2;26(1):1–26.
29. Tsao JC, Jacob E, Seidman LC, Lewis MA, Zeltzer LK. Psychological aspects and hospitalization for pain crises in youth with sickle-cell disease. J Health Psychol. 2014 Mar;19(3):407–16.
30. Koechlin H, Donado C, Locher C, Kossowsky J, Lionetti F, Pluess M. Sensory processing sensitivity in adolescents reporting chronic pain: an exploratory study. Pain Rep. 2023 Jan 6;8(1):e1053.
31. Gross JJ, John OP. Individual differences in two emotion regulation processes: implications for affect, relationships, and well-being. J Pers Soc Psychol. 2003 Aug;85(2):348–62.
32. English T, Lee IA, John OP, Gross JJ. Emotion regulation strategy selection in daily life: The role of social context and goals. Motiv Emot. 2017 Apr;41(2):230–42.
33. Brans K, Koval P, Verduyn P, Lim YL, Kuppens P. The regulation of negative and positive affect in daily life. Emotion. 2013 Oct;13(5):926–39.
34. Poppert Cordts KM, Stone AL, Beveridge JK, Wilson AC, Noel M. The (Parental) Whole Is Greater Than the Sum of Its Parts: A Multifactorial Model of Parent Factors in Pediatric Chronic Pain. J Pain. 2019 Jul;20(7):786–95.
35. Simons LE, Smith A, Kaczynski K, Basch M. Living in fear of your child's pain: the Parent Fear of Pain Questionnaire. Pain. 2015 Apr;156(4):694–702.
36. Chambers CT, Craig KD, Bennett SM. The Impact of Maternal Behavior on

For peer review only - http://bmjopen.bmj.com/site/about/guidelines.xhtml

Children's Pain Experiences: An Experimental Analysis. J Pediatr Psychol. 2002 Apr 1;27(3):293–301.

37. Palermo TM, Chambers CT. Parent and family factors in pediatric chronic pain and disability: An integrative approach. PAIN. 2005 Dec;119(1–3):1–4.

38. Bariola E, Gullone E, Hughes EK. Child and adolescent emotion regulation: the role of parental emotion regulation and expression. Clin Child Fam Psychol Rev. 2011 Jun;14(2):198–212.

39. Morris AS, Silk JS, Steinberg L, Myers SS, Robinson LR. The Role of the Family Context in the Development of Emotion Regulation. Soc Dev. 2007 May 1;16(2):361–88.

40. Ellyson AM, Gordon G, Zhou C, Rabbitts JA. Trajectories, Risk Factors, and Impact of Persistent Pain After Major Musculoskeletal Surgery in Adolescents: A Replication Study. The Journal of Pain [Internet]. 2021 Dec 31 [cited 2022 Jun 2]; Available from: https://www.sciencedirect.com/science/article/pii/S1526590021003916

41. Goubert L, Trompetter H. Towards a science and practice of resilience in the face of pain. European Journal of Pain. 2017;21(8):1301–15.

42. Williams G, Howard RF, Liossi C. Persistent postsurgical pain in children and young people: prediction, prevention, and management. Pain Rep. 2017 Aug 21;2(5):e616.

43. Christodoulou E, van Smeden M, Edlinger M, Timmerman D, Wanitschek M, Steyerberg EW, et al. Adaptive sample size determination for the development of clinical prediction models. Diagnostic and Prognostic Research. 2021 Mar 22;5(1):6.

44. Riley RD, Ensor J, Snell KIE, Harrell FE, Martin GP, Reitsma JB, et al. Calculating the sample size required for developing a clinical prediction model. BMJ. 2020 Mar 18;368:m441.

45. Confederation TFA of the S. Federal Act on Research involving Human Beings: Human Research Act (HRA). 801.30 Sep 1, 2013.

46. Harris PA, Taylor R, Thielke R, Payne J, Gonzalez N, Conde JG. Research electronic data capture (REDCap) - A metadata-driven methodology and workflow process for providing translational research informatics support. Journal of Biomedical Informatics. 2009;42(2):377–81.

47. Koval P, Hinton J, Dozo N, Gleeson J, Alvarez M, Harrison A, et al. SEMA3: Smartphone Ecological Momentary Assessment. 2019.

48. Watson D, Clark LA, Tellegen A. Development and validation of brief measures of positive and negative affect: The PANAS scales. Journal of Personality and Social Psychology. 1988;54(6):1063–70.

49. Laurent J, Catanzaro SJ, Joiner Jr. TE, Rudolph KD, Potter KI, Lambert S, et al. A measure of positive and negative affect for children: Scale development and preliminary validation. Psychological Assessment. 1999;11(3):326–38.

50. Wilson K, Gullone E, Moss S. The Youth Version of the Positive and Negative Affect Schedule: A Psychometric Validation. Behaviour Change. 1998 Sep;15(3):187–93.

51. Simons LE, Smith A, Ibagon C, Coakley R, Logan DE, Schechter N, et al. Pediatric Pain Screening Tool (PPST): Rapid identification of risk in youth with pain complaints. Pain. 2015 Aug;156(8):1511–8.

52. Narayanasamy S, Yang F, Ding L, Geisler K, Glynn S, Ganesh A, et al. Pediatric Pain Screening Tool: A Simple 9-Item Questionnaire Predicts Functional and Chronic Postsurgical Pain Outcomes After Major Musculoskeletal Surgeries. The Journal of Pain [Internet]. 2021 Jul 17 [cited 2021 Sep 24]; Available from: https://www.sciencedirect.com/science/article/pii/S1526590021002911

53. Flack F, Gerlach AL, Simons LE, Zernikow B, Hechler T. Validation of the German fear of pain questionnaire in a sample of children with mixed chronic pain conditions. Eur J Pain. 2017;21(7):1224–33.

54. Offenbächer M, Kohls N, Walker L, Hermann C, Hügle B, Jäger N, et al. Functional

For peer review only - http://bmjopen.bmj.com/site/about/guidelines.xhtml

limitations in children and adolescents suffering from chronic pain: validation and psychometric properties of the German Functional Disability Inventory (FDI-G). Rheumatol Int. 2016 Oct 1;36(10):1439–48.
55. Kerns KA, Klepac L, Cole A. Security Scale. Developmental Psychology [Internet]. 1996 [cited 2023 Apr 13]; Available from: https://psycnet.apa.org/doiLanding?doi=10.1037%2Ft17560-000
56. Ebesutani C, Reise SP, Chorpita BF, Ale C, Regan J, Young J, et al. The Revised Child Anxiety and Depression Scale-Short Version: Scale reduction via exploratory bifactor modeling of the broad anxiety factor. Psychological Assessment. 2012;24(4):833–45.
57. Buysse DJ, Reynolds CFI, Monk TH, Berman SR, Kupfer DJ. The Pittsburgh Sleep Quality Index: A new instrument for psychiatric practice and research. Psychiatry Research. 1988;28:193–213.
58. Pluess M, Assary E, Lionetti F, Lester KJ, Krapohl E, Aron EN, et al. Environmental sensitivity in children: Development of the Highly Sensitive Child Scale and identification of sensitivity groups. Dev Psychol. 2018;54(1):51–70.
59. Varni JW, Seid M, Rode CA. The PedsQL™: Measurement Model for the Pediatric Quality of Life Inventory. Medical Care. 1999 Feb;37(2):126.
60. Gordon-Hollingsworth AT, Thompson JE, Geary MA, Schexnaildre MA, Lai BS, Kelley ML. Social Support Questionnaire for Children. Measurement and Evaluation in Counseling and Development. 2016 Apr 1;49(2):122–44.
61. Sachser C, Berliner L, Risch E, Rosner R, Birkeland MS, Eilers R, et al. The child and Adolescent Trauma Screen 2 (CATS-2) – validation of an instrument to measure DSM-5 and ICD-11 PTSD and complex PTSD in children and adolescents. European Journal of Psychotraumatology. 2022 Dec 19;13(2):2105580.
62. Chambers CT, Reid GJ, McGrath PJ, Finley GA. Development and preliminary validation of a postoperative pain measure for parents 11This paper was presented at the meeting Pediatric Pain: Challenges, Innovations, and Costs in Atlanta, GA, November 4, 1995 (Voted Best Abstract). Pain. 1996 Dec 1;68(2):307–13.
63. Snaith RP. The Hospital Anxiety And Depression Scale. Health and Quality of Life Outcomes. 2003 Aug 1;1(1):29.
64. Aron EN, Aron A. Sensory-processing sensitivity and its relation to introversion and emotionality. J Pers Soc Psychol. 1997 Aug;73(2):345–68.
65. Pluess M. Sensory-Processing Sensitivity: A potential mechanism of differential susceptibility. In: Biennial Meeting of the Society for Research in Child Development (SRCD), Seattle, WA. 2013.
66. Bech P, Olsen LR, Kjoller M, Rasmussen NK. Measuring well-being rather than the absence of distress symptoms: a comparison of the SF-36 Mental Health subscale and the WHO-Five well-being scale. International Journal of Methods in Psychiatric Research. 2003;12(2):85–91.
67. Treede RD, Rief W, Barke A, Aziz Q, Bennett MI, Benoliel R, et al. Chronic pain as a symptom or a disease: the IASP Classification of Chronic Pain for the International Classification of Diseases (ICD-11). Pain. 2019 Jan;160(1):19–27.
68. Durand H, Birnie KA, Noel M, Vervoort T, Goubert L, Boerner KE, et al. State Versus Trait: Validating State Assessment of Child and Parental Catastrophic Thinking About Children's Acute Pain. The Journal of Pain. 2017 Apr 1;18(4):385–95.
69. Kerns KA, Klepac L, Cole A. Peer relationships and preadolescents' perceptions of security in the child-mother relationship. Developmental Psychology. 1996 May;32(3):457–66.
70. Sachser C, Berliner L, Holt T, Jensen TK, Jungbluth N, Risch E, et al. International development and psychometric properties of the Child and Adolescent Trauma Screen (CATS). J Affect Disord. 2017 Mar 1;210:189–95.

For peer review only - http://bmjopen.bmj.com/site/about/guidelines.xhtml

71. Rabbitts JA, Palermo TM, Zhou C, Meyyappan A, Chen L. Psychosocial Predictors of Acute and Chronic Pain in Adolescents Undergoing Major Musculoskeletal Surgery. The Journal of Pain. 2020 Nov 1;21(11):1236–46.
72. Rabbitts JA, Palermo TM, Zhou C, Mangione-Smith R. Pain and Health-Related Quality of Life After Pediatric Inpatient Surgery. The Journal of Pain. 2015 Dec 1;16(12):1334–41.
73. Werner MU, Kongsgaard UE. I. Defining persistent post-surgical pain: is an update required? Br J Anaesth. 2014 Jul;113(1):1–4.
74. Camino Willhuber G, Slullitel P, Taype Zamboni D, Albergo J, Terrasa S, Piuzzi N, et al. Validation of a modified Clavien-Dindo Classification for postoperative complications in orthopedic surgery. Rev Fac Cien Med Univ Nac Cordoba. 2020 Aug 21;77(3):161–7.
75. Steyerberg EW, Vickers AJ, Cook NR, Gerds T, Gonen M, Obuchowski N, et al. Assessing the performance of prediction models: a framework for some traditional and novel measures. Epidemiology. 2010 Jan;21(1):128–38.
76. Gerds TA, Cai T, Schumacher M. The performance of risk prediction models. Biom J. 2008 Aug;50(4):457–79.
77. Rubin DB. Inference and missing data. Biometrika. 1976 Dec 1;63(3):581–92.
78. Little RJA, Rubin DB. Statistical Analysis with Missing Data. John Wiley & Sons; 2019. 465 p.
79. Genolini C, Alacoque X, Sentenac M, Arnaud C. kml and kml3d: R Packages to Cluster Longitudinal Data. Journal of Statistical Software. 2015 Jun 1;65(1):1–34.
80. Donado C, Friedrich Y, Kossowsky J, Locher C, Koechlin H. Exposure to Parental Depressive Symptoms: A Longitudinal Analysis on the Association with Adolescents' Depressive Symptoms and Adjustment Problems. J Dev Behav Pediatr. 2020 Sep;41(7):522–33.

For peer review only

For peer review only - http://bmjopen.bmj.com/site/about/guidelines.xhtml

Authors' contributions

HK has designed the study, obtained funding, wrote the first draft of the manuscript, and acts as project leader and sponsor for the study described in this protocol.

JH has collaborated closely with HK in the process of detailing the study plan, has written and revised the manuscript, and is the PhD student in this project.

CCH, TD, and SC are the clinical collaborators of this study. They have all provided substantial input in the research plan and the study procedures, and have critically revised the manuscript.

JR has provided substantial input and feedback on the research plan, choice of outcome variables, and study procedures, and has critically revised the manuscript.

CL has provided substantial feedback on the design of the study and all study related procedures, and has critically revised the manuscript.

UH is responsible for data analysis in this project, has written the data analysis plan, provided advice on the power calculation, and has critically revised the manuscript.

All authors approved the final version of the manuscript, and agree to be accountable for all aspects of the work in ensuring that questions related to the accuracy or integrity of any part of the work are appropriately investigated and resolved.

Funding statement

This work is supported by a Swiss National Science Foundation grant awarded to HK: PZ00P1_208850 (Title: "Understanding and preventing chronic postsurgical pain in children")

JR is supported by National Institutes of Health National Institutes of Arthritis, Musculoskeletal, and Skin Diseases under award number by K24AR080786 (PI: J. Rabbitts). The content is solely the responsibility of the authors and does not necessarily represent the official views of the National Institutes of Health.

Competing interest statement

The authors report no conflict of interest.

For peer review only

Figure 1. Overview of Assessment Time Points

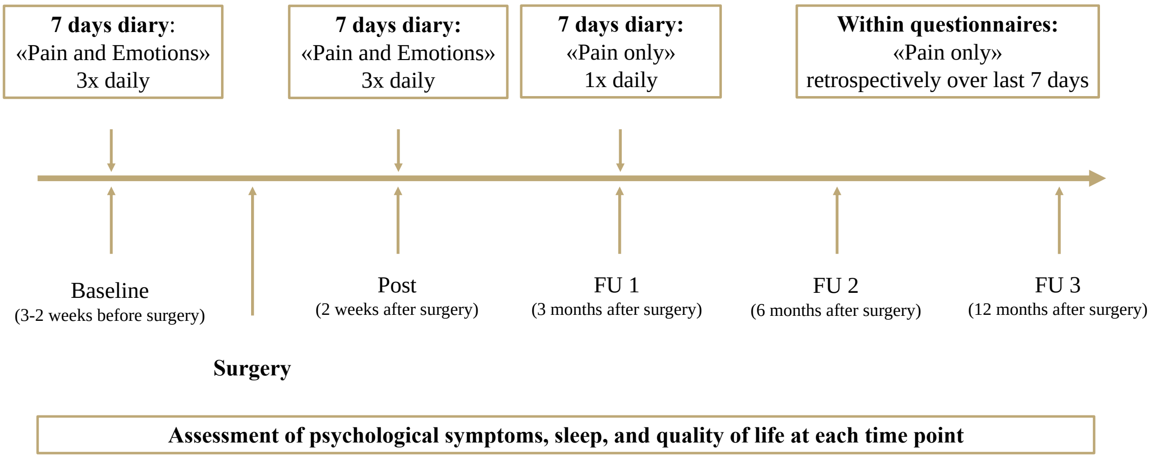

For peer review only

Supplement: Overview of reliability, validity, benefits, and limitations of questionnaires used in the study.

Domain		Measure	Reliability	Validity	Benefits	Limitations
**Experience-sampling method**	Positive and negative emotions*	Items from the Positive and Negative Affect Schedule (1)	Cronbach's Alpha high, .86 for PA and .87 for NA scale (1).	Excellent factorial validity (convergent correlation high: .89 to .95; discriminant correlation low: -.02 to -.18); items good markers of corresponding factors (1).	External validity demonstrated by correlation with other scales. Stability over 2-month time period (1).	Heterogeneity the factorial structure of the PANAS scores (2).
	Pain severity	Numeric Rating Scale (NRS), 0-10				
**Psychosocial factors child**						
	Pain severity (intensity, distress, inter-ference)*	Numeric Rating Scale (NRS), 0-10			Individual scores of pain severity can be converted into the ICD-11 categories of none (0), mild (1), moderate (2) or severe (3) and merged into one extension code endorsed by the WHO (3,4).	More field testing is needed to further validate NRS scales in pediatric populations (5).
	Pre-operative screening	Pediatric Pain Screening Tool (6,7)	Overall PPST: adequate to excellent discrimination; psychosocial subscale AUC	Sensitivity and specificity for the PPST ranged from adequate to excellent, with regard to significant disability (78%, 68%) and high	Defines risk groups to inform efficient treatment decision-making; valid screening tool that allows early	Generalizability of results needs further studies in surgeries with different pathology and requires further

		adequate to acceptable (6). Total score (ICC = 0.75) and psychosocial subscale (ICC = 0.70) acceptable 2-week test-retest reliability (6).	emotional distress (81%, 63%) (6).	identification of patients presenting with impairing pain-related comorbidities (6).	stratification by race and surgical/pain characteristics, and interventions (7). German translation not yet validated.
Functional Disability	Functional Disability Index (FDI; (8))	Good internal reliability of the total FDI in the German cohort (Cronbach's α = .9) similar to the original US version (8).	Good construct validity with significant correlations between the FDI and different variables (e.g. Childhood Health Assessment Questionnaire [total score], pain-related interference in everyday life, affective distress) (8).	Tested in a large cohort of German children and adolescents. Data confirm that the German version of the FDI retains the characteristics of the original FDI and is a reliable and valid instrument (8).	Majority of females subjects; inclusion of patients from a single specialized center restrict the generalizability of the results (8).
Fear of Pain*	Fear of Pain Questionnaire (9)	Good internal consistency of the total scale Cronbach's α = 0.87. Internal consistency also good for both subscales (Fear subscale: α = 0.89; avoidance subscale: α = 0.76) (9)	The two-factor structure of the GFOPQ-C shows that fear of pain includes two key dimensions: Fear of Pain and Avoidance of Activities (9).	Accurate translation of the English version conducted on the basis of the forward–backward translation. Instrument applied in a mixed pain sample (9).	To evaluate the degree of increased fear of pain in pain samples, it would be beneficial a comparison to healthy controls (9)

Pain catastrophizing *	Pain Catastrophizing Scale – 3 item version (10)	Chronbach's Alpha high α = .84 and .79 for PCS-C state (10)	Significant relationships with child pain-related outcomes (ie, child pain intensity and child state anxiety), no issue with multicollinearity (10)	Short (3-item scale) can be efficiently used (= high correlation with 6-item scale). Large, diverse sample of children (10)	Due to small number of items per factor, additional research needed to support the 1-factor model. (10)
Attachment parent–child relationship *	Security Scale (11)	Cronbach's α = .74 for both mothers and fathers of sixth graders; and α = .75 and α = .52 for mothers and fathers of third graders respectively (11)	Moderate stability and meaningful associations with other attachment measures, caregiver sensitivity, and indexes of child's adjustment (1).	One of the most widely used measures of attachment in middle childhood and early adolescence. Some associations of the scale with child's outcomes hold regardless of the child's sex and age (12).	Many cross-sectional rather than longitudinal studies (12).
Symptoms of anxiety and depression*	Revised Child Anxiety and Depression Scale-short version (13).	15-item Anxiety Total scale evidenced significant correspondence with anxiety diagnostic groups,10-item Depression Total scale associated with acceptable reliability (Cronbach's α =	Based on a cross-sample validation strategy, values fell in the "acceptable" classification category (AUC=.74, SE=.03) (13).	Potentially useful for repeated measurement in clinical settings as well as wide-scale screenings. Assesses both anxiety and depressive symptoms (13).	Additional test development efforts needed to ensure that the shortened RCADS meets the clinical demands of reduced test length, while maintaining adequate reliability of its broad scale scores (13).

		.80 and .79, respectively) (13).			
Sleep*	Pittsburgh Sleep Quality Index (PSQI; (14))	High internal consistency (Cronbach's α = 0.83) (14)	Sensitive and specific measure: Global PSQI score > 5, sensitivity of 89.6% and specificity of 86.5% (kappa = 0.75, p < 0.001) (14)	Provides a standardized, quantitative measure of sleep quality that quickly identifies good and poor sleepers (14)	Adult population sample (14)
Sensory processing sensitivity	Highly Sensitive Child Scale (15)	HSC-12-items: acceptable internal consistency with Cronbach's α = .71 and .74. The subscales showed lower internal consistency with α = .73/.69 (15).	Divergent validity was established (15).	12-item HSC scale is a psychometrically robust measure that performs well in both children and adolescents. Studies with large sample, replication of results, previous use in samples with chronic pain (15,16).	All data are based on self-report. Most data were provided by children and adolescents residing in the United Kingdom (15).
Emotion regulation*	Emotion Regulation Questionnaire – Child and Adolescent version (17,18)	Sound internal consistency: cognitive reappraisal subscale Cronbach's α = .82, expressive suppression subscale α = .79 (17).	Good factor structure, convergent validity supported (17).	Tested on participants aged between 10 and 18 years (17), previously used in samples with chronic pain (16,19).	Measures only two emotion regulation strategies, cognitive reappraisal and expressive suppression (20).
Quality of life	Pediatric Quality of Life Inventory (21)	High internal consistency (Cronbach's α =	Across scales, item-level internal consistency supported content	Cronbach α good in various samples, including	Issue of detecting small changes in quality of life only

			0.83 for self-report and α = 0.86 for parent-report). α for self-report ranged from .70 to .89 (19).	validity. Item-level discriminant validity tests showed more variability (21)	adolescent samples with chronic pain (16).	partially addressed, more longitudinal data could help to better understand this aspect (21).
	Social support*	Social Support Questionnaire for Children (22)	High internal consistency. Cronbach's α for total scale and five subscales ranging from .89 to .97 (23).	Good factorial and construct validity.Convergent validity was partially supported (23).	The SSQC identifies important sources of social support in varying samples (e.g. for particular ethnic populations the extended family and community member (23).	Substantially longer than a widely used measure of social support in children (23).
	Posttraumatic stress symptoms	Child and Adolescent Trauma Screening (CATS) (24).	Good to excellent reliability with Cronbach's α ranging between .88 and .94 for all three language samples (US, Germany, Norway) (25).	Convergent discriminant validity: medium to strong correlations with measures of depression (r =.62–.82) and anxiety (r =.40–.77). Low to medium correlations with externalizing symptoms (r =−.15–.43) (25).	Different countries involved in the validation (USA, Germany, and Norway) (25).	Pending external validation with a DSM-5 based semi-structured clinical interview (25).
**Psychosocial factors parents**						
	Parents' own pain history*	2 questions: "(1) Have you ever and (2) are you currently				

	living with persistent or recurrent pain for at least 3 months?”				
Postoperative pain measure (child’s pain)	Postoperative Pain Measure for Parents (26)	Cronbach’s α = .88 and .87 on postoperative days 1 and 2, respectively (26).	Excellent sensitivity (>80%) and specificity (>80%) in children who reported clinically significant levels of pain (26).	The minimal training required for parents to use this tool supports its utility as a clinical tool (26).	The items were developed based on parent reports and thus could miss important behavior changes in children (26).
Fear of pain	Parent Fear of Pain Questionnaire (27)	The 21-item scale showed a Cronbach's α = .91(27).	Solid construct validity, supported for the subscales (27).	Excellent fit for a complex model, after a minor modification to include parent avoidance as an indicator of parent behavior (27).	Tested in samples of predominantly mothers (92%), Caucasian (90%) and female children (74.8%) (27).
Pain cata-strophizing *	Pain Cata-strophizing Scale – 3 item version (10)	Chronbach’s Alpha high α = .79 and .73 for PCS-P state (10).	Significant relationships with parent pain-related outcomes (i.e., parental distress), no issue with multicollinearity (10).	Short (3-item scale) can be efficiently used (= high correlation with 6-item scale). Large, diverse sample of parents (10).	Due to small number of items per factor, additional research needed to support the 1-factor model (10).
Anxiety and depression*	Anxiety and Depression Scale (28)	Cronbach's alpha high α = .78 for the depression scale and α = .81 for the anxiety scale (29).	Original model (28) demonstrates the best fit. Good discriminant validity in large study and across various populations and countries (29).	Simple and reliable tool for medical practice. In addition to validation for use in the elderly the HADS has been validated for use in adolescents (28).	Few studies validated the questionnaire with a general population or included a control group taken from

					the general population (29).
Sensory processing sensitivity	Highly Sensitive Person Scale (30,31)	Reliability of 27-item version Cronbach's α = .87 and .85 in 2 different studies (31).	The 27-item version showed good content validity and the measure's discriminant, convergent (31). Study confirmed that the HSP scale reflects a unitary dimension of environmental sensitivity and identified in addition three sensitivity groups in the general population (high, low and medium sensitivity) (32).	Diversity of samples as well as qualitative and quantitative methods used to develop scale and support conlcusions (31).	Parental impact on sensitive individuals not explored in depth (31).
Emotion regulation*	Emotion Regulation Questionnaire (33)	Cronbach's alpha satisfactory α = .74 for cognitive reappraisal and .76 for expressive suppression (34).	Indices of CFA were satisfactory for the German sample; support for measurement invariance of the ERQ (35).	Good conformity of the translation with the original (34).	Mainly tested in student samples (36).
Quality of life	World Health Organization Well-Being Index (37)	Internal consistency of the WHO-Five and the Mental Health subscale acceptable (Cronbach's α = 0.84. and 0.8) (37).	Adequate validity both as a screening tool for depression and as an outcome measure in clinical trials (38).	Includes subscales measuring both physical health and mental health. Compare a scale containing a mixture of distress and well being items (37). Validated in both younger and	Has been researched predominantly in the field of depression (38).

		elderly persons (38).
**Primary outcome**		
Chronic postsurgical pain	Presence of CPSP: pain diary for 7 consecutive days (NRS) + health-related quality of life (PedsQL)	

Note. AUC = area under the curve , ICC = intraclass correlation coefficient, PCS-C = Pain Catastrophizing Scale for Children, PCS-P = Pain Catastrophizing Scale for Parents.

For peer review only

References

1. Watson D, Clark LA, Tellegen A. Development and validation of brief measures of positive and negative affect: The PANAS scales. J Pers Soc Psychol. 1988;54(6):1063–70.
2. Flores-Kanter PE, Garrido LE, Moretti LS, Medrano LA. A modern network approach to revisiting the Positive and Negative Affective Schedule (PANAS) construct validity. J Clin Psychol. 2021;77(10):2370–404.
3. Barke A, Koechlin H, Korwisi B, Locher C. Emotional distress: Specifying a neglected part of chronic pain. Eur J Pain. 2020;24(3):477–80.
4. Treede RD, Rief W, Barke A, Aziz Q, Bennett MI, Benoliel R, et al. Chronic pain as a symptom or a disease: the IASP Classification of Chronic Pain for the International Classification of Diseases (ICD-11). PAIN. 2019 Jan;160(1):19.
5. McGrath PJ, Walco GA, Turk DC, Dworkin RH, Brown MT, Davidson K, et al. Core Outcome Domains and Measures for Pediatric Acute and Chronic/Recurrent Pain Clinical Trials: PedIMMPACT Recommendations. J Pain. 2008 Sep 1;9(9):771–83.
6. Simons LE, Smith A, Ibagon C, Coakley R, Logan DE, Schechter N, et al. Pediatric Pain Screening Tool (PPST): Rapid identification of risk in youth with pain complaints. Pain. 2015 Aug;156(8):1511–8.
7. Narayanasamy S, Yang F, Ding L, Geisler K, Glynn S, Ganesh A, et al. Pediatric Pain Screening Tool: A Simple 9-Item Questionnaire Predicts Functional and Chronic Postsurgical Pain Outcomes After Major Musculoskeletal Surgeries. J Pain [Internet]. 2021 Jul 17 [cited 2021 Sep 24]; Available from: https://www.sciencedirect.com/science/article/pii/S1526590021002911
8. Offenbächer M, Kohls N, Walker L, Hermann C, Hügle B, Jäger N, et al. Functional limitations in children and adolescents suffering from chronic pain: validation and psychometric properties of the German Functional Disability Inventory (FDI-G). Rheumatol Int. 2016 Oct 1;36(10):1439–48.
9. Flack F, Gerlach AL, Simons LE, Zernikow B, Hechler T. Validation of the German fear of pain questionnaire in a sample of children with mixed chronic pain conditions. Eur J Pain Lond Engl. 2017;21(7):1224–33.
10. Durand H, Birnie KA, Noel M, Vervoort T, Goubert L, Boerner KE, et al. State Versus Trait: Validating State Assessment of Child and Parental Catastrophic Thinking About Children’s Acute Pain. J Pain. 2017 Apr 1;18(4):385–95.
11. Kerns KA, Klepac L, Cole A. Peer relationships and preadolescents’ perceptions of security in the child-mother relationship. Dev Psychol. 1996 May;32(3):457–66.
12. Brumariu LE, Madigan S, Giuseppone KR, Movahed Abtahi M, Kerns KA. The Security Scale as a measure of attachment: Meta-analytic evidence of validity. Attach Hum Dev. 2018;20(6):600–25.
13. Ebesutani C, Reise SP, Chorpita BF, Ale C, Regan J, Young J, et al. The Revised Child Anxiety and Depression Scale-Short Version: Scale reduction via exploratory bifactor modeling of the broad anxiety factor. Psychol Assess. 2012;24(4):833–45.
14. Buysse DJ, Reynolds CFI, Monk TH, Berman SR, Kupfer DJ. The Pittsburgh Sleep Quality Index: A new instrument for psychiatric practice and research. Psychiatry Res. 1988;28:193–213.
15. Pluess M, Assary E, Lionetti F, Lester KJ, Krapohl E, Aron EN, et al. Environmental sensitivity in children: Development of the Highly Sensitive Child Scale and identification of sensitivity groups. Dev Psychol. 2018;54(1):51–70.

For peer review only

16. Koechlin H, Donado C, Locher C, Kossowsky J, Lionetti F, Pluess M. Sensory processing sensitivity in adolescents reporting chronic pain: an exploratory study. Pain Rep. 2023 Jan 6;8(1):e1053.
17. Gullone E, Taffe J. The Emotion Regulation Questionnaire for Children and Adolescents (ERQ-CA): a psychometric evaluation. Psychol Assess. 2012 Jun;24(2):409–17.
18. Abler B, Kessler H. Emotion Regulation Questionnaire – Eine deutschsprachige Fassung des ERQ von Gross und John. Diagnostica. 2009 Jul 1;55(3):144–52.
19. Koechlin H, Beeckman M, Meier AH, Locher C, Goubert L, Kossowsky J, et al. Association of parental and adolescent emotion-related factors with adolescent chronic pain behaviors. PAIN [Internet]. 2022 [cited 2021 Oct 28]; Available from: https://journals.lww.com/pain/Abstract/9000/Association_of_parental_and_adolescent.97860.aspx
20. Dorn C, Spindler G, Kullik A, Petermann F, Barnow S. Erfassung von Emotionsregulationsstrategien–eine Übersicht. Psychol Rundsch. 2013 Oct;64(4):217–27.
21. Varni JW, Seid M, Rode CA. The PedsQL™: Measurement Model for the Pediatric Quality of Life Inventory. Med Care. 1999 Feb;37(2):126.
22. Gordon-Hollingsworth AT, Thompson JE, Geary MA, Schexnaildre MA, Lai BS, Kelley ML. Social Support Questionnaire for Children. Meas Eval Couns Dev. 2016 Apr 1;49(2):122–44.
23. Gordon-Hollingsworth AT, Thompson JE, Geary MA, Schexnaildre MA, Lai BS, Kelley ML. Social Support Questionnaire for Children: Development and Initial Validation. Meas Eval Couns Dev. 2016 Apr;49(2):122–44.
24. Sachser C, Berliner L, Risch E, Rosner R, Birkeland MS, Eilers R, et al. The child and Adolescent Trauma Screen 2 (CATS-2) – validation of an instrument to measure DSM-5 and ICD-11 PTSD and complex PTSD in children and adolescents. Eur J Psychotraumatology. 2022 Dec 19;13(2):2105580.
25. Sachser C, Berliner L, Holt T, Jensen TK, Jungbluth N, Risch E, et al. International development and psychometric properties of the Child and Adolescent Trauma Screen (CATS). J Affect Disord. 2017 Mar 1;210:189–95.
26. Chambers CT, Reid GJ, McGrath PJ, Finley GA. Development and preliminary validation of a postoperative pain measure for parents 11This paper was presented at the meeting Pediatric Pain: Challenges, Innovations, and Costs in Atlanta, GA, November 4, 1995 (Voted Best Abstract). Pain. 1996 Dec 1;68(2):307–13.
27. Simons LE, Smith A, Kaczynski K, Basch M. Living in fear of your child's pain: the Parent Fear of Pain Questionnaire. Pain. 2015 Apr;156(4):694–702.
28. Snaith RP. The Hospital Anxiety And Depression Scale. Health Qual Life Outcomes. 2003 Aug 1;1(1):29.
29. Bocéréan C, Dupret E. A validation study of the Hospital Anxiety and Depression Scale (HADS) in a large sample of French employees. BMC Psychiatry. 2014;14(1):1–11.
30. Pluess M. Sensory-Processing Sensitivity: A potential mechanism of differential susceptibility. In: Biennial Meeting of the Society for Research in Child Development (SRCD), Seattle, WA. 2013.

For peer review only

31. Aron EN, Aron A. Sensory-processing sensitivity and its relation to introversion and emotionality. J Pers Soc Psychol. 1997 Aug;73(2):345–68.
32. Lionetti F, Aron A, Aron EN, Burns GL, Jagiellowicz J, Pluess M. Dandelions, tulips and orchids: Evidence for the existence of low-sensitive, medium-sensitive and high-sensitive individuals. Transl Psychiatry. 2018;8(1):24.
33. Gross JJ, John OP. Individual differences in two emotion regulation processes: implications for affect, relationships, and well-being. J Pers Soc Psychol. 2003 Aug;85(2):348–62.
34. Abler B, Kessler H. Emotion regulation questionnaire–Eine deutschsprachige Fassung des ERQ von Gross und John. Diagnostica. 2009;55(3):144–52.
35. Sala MN, Molina P, Abler B, Kessler H, Vanbrabant L, van de Schoot R. Measurement invariance of the Emotion Regulation Questionnaire (ERQ). A cross-national validity study. Eur J Dev Psychol. 2012;9(6):751–7.
36. Preece DA, Becerra R, Robinson K, Gross JJ. The emotion regulation questionnaire: psychometric properties in general community samples. J Pers Assess. 2019;
37. Bech P, Olsen LR, Kjoller M, Rasmussen NK. Measuring well-being rather than the absence of distress symptoms: a comparison of the SF-36 Mental Health subscale and the WHO-Five well-being scale. Int J Methods Psychiatr Res. 2003;12(2):85–91.
38. Topp CW, Østergaard SD, Søndergaard S, Bech P. The WHO-5 Well-Being Index: a systematic review of the literature. Psychother Psychosom. 2015;84(3):167–76.

For peer review only

Longitudinal Resilience and Risk Factors in Pediatric Postoperative Pain (LORRIS): Protocol for a Prospective Longitudinal Swiss University Children's Hospitals-Based Study

Journal:	*BMJ Open*
Manuscript ID	bmjopen-2023-080174.R2
Article Type:	Protocol
Date Submitted by the Author:	10-Mar-2024
Complete List of Authors:	Hochreuter, Jana; University Children's Hospital Zurich, Department of Psychosomatics and Psychiatry; University of Zurich, Department of Psychology<br>Dreher, Thomas ; University Children's Hospital Zürich, Department of Pediatric Orthopedics and Traumatology; Balgrist University Hospital, Department of Pediatric Orthopedics<br>Hasler, Carol-Claudius; Children’s Hospital Basel, Department of Orthopedics<br>Canonica, Sandro; University Children's Hospital Zürich, Pediatric Orthopedics and Traumatology; University Hospital Balgrist Zürich, Department of Pediatric Orthopedics<br>Locher, Cosima; University Hospital Zurich, Department of Consultation-Liaison Psychiatry and Psychosomatic Medicine; Plymouth University<br>Held, Ulrike; Epidemiology, Biostatistics and Prevention Institute, Biostatistics<br>Rabbitts, Jennifer; Stanford University, Anesthesiology Pediatric Anesthesiology<br>Koechlin, Helen; University Children's Hospital Zürich, Psychosomatics and Psychiatry; University of Zurich, Department of Psychology
**Primary Subject Heading**:	Paediatrics
Secondary Subject Heading:	Surgery
Keywords:	SURGERY, Adolescents < Adolescent, Chronic Pain, Risk Factors

Longitudinal Resilience and Risk Factors in Pediatric Postoperative Pain (LORRIS): Protocol for a Prospective Longitudinal Swiss University Children's Hospitals-Based Study

Hochreuter, J.[1,2,3], Dreher, T.[3,4,5], Hasler, C.-C.[6], Canonica, S.[3,4,5], Locher, C.[7,8], Held, U.[9], Rabbitts, J.A.[10], & Koechlin, H.[1,2,3]

[1]Department of Psychosomatics and Psychiatry, University Children's Hospital, University of Zurich, Zurich, Switzerland.

[2]Division of Child and Adolescent Health Psychology, Department of Psychology, University of Zurich, Zurich, Switzerland.

[3]Children's Research Centre, University Children's Hospital Zurich, University of Zurich, Zurich, Switzerland.

[4]Department of Pediatric Orthopedics and Traumatology, University Children's Hospital Zurich, Switzerland.

[5]Department of Pediatric Orthopedics, Orthopedic University Hospital Balgrist, Zurich, Switzerland

[6]Department of Orthopedics, Children's Hospital Basel, Switzerland.

[7]Department of Consultation-Liaison Psychiatry and Psychosomatic Medicine, University Hospital Zurich, Zurich, Switzerland.

[8]Faculty of Health, University of Plymouth, UK.

[9]Department of Biostatistics and Epidemiology, Biostatistics and Prevention Institute, University of Zurich, Switzerland.

[10]Anesthesiology, Perioperative and Pain Medicine, Pediatric Anesthesiology, Stanford University, USA.

Corresponding author: Helen Koechlin, PhD, University Children's Hospital Zurich, Bergstrasse 57, 8032 Zurich, Switzerland. Email address: helen.koechlin@kispi.uzh.ch

For peer review only - http://bmjopen.bmj.com/site/about/guidelines.xhtml

Abstract

Introduction

Chronic postsurgical pain (CPSP) is defined as pain that persists after a surgical procedure, and has significant impact on quality of life. Previous studies show the importance of psychological factors in CPSP, yet the majority of studies focused solely on negative emotions. This longitudinal observational study aims to broaden this knowledge base by examining the role of emotional state, emotion variability, emotion regulation, and emotion differentiation on the child- and the parent-level for the development CPSP, and to describe pain and emotion-related trajectories following surgery.

Methods and analysis

We intend to include 280 children and adolescents aged 8-18 years with a planned orthopedic surgery and their parents. A total of five assessment time points is planned: 3 weeks before surgery (baseline), 2 weeks after surgery (post), and 3 months (Follow-up [FU] 1), 6 months and 12 months after surgery. At baseline and post only, children and parents are asked to complete a daily diary thrice a day for a week where they rate their current emotional state and their pain severity (children only). Emotional state ratings will be used to calculate indices of emotion variability, emotion regulation, and emotion differentiation. Children and parents will complete questionnaires at each time point, including measures on quality of life, social support, sleep, and symptoms of anxiety and depression.

To predict development of CPSP, generalized linear regression models will be used, resulting in odds ratios and 95% confidence intervals. Pearson product-moment correlations between predictors and outcomes will be evaluated at each time point. The primary outcome of the prediction model is CPSP at FU1. For the trajectory analysis, the classification method K-means for longitudinal data will be used to determine clusters in the data.

Ethics and dissemination

The Ethics Committee of the Canton of Zurich, Switzerland, has approved the study (ID: 2023-01475). Participants will be compensated, and a dissemination workshop will be held.

Registration details

Clinicaltrials.gov: NCT05816174

For peer review only - http://bmjopen.bmj.com/site/about/guidelines.xhtml

Strengths and limitations of this study

- This is a prospective, multicenter, longitudinal hospital-based study and the sample size allows to examine a range of risk and resilience factors.
- The longitudinal study design permits measurement of pain- and emotion-related trajectories up until 12 months after surgery.
- The repeated assessments design will lead to an in-depth understanding of changes of risk and resilience factors over time.
- The primary limitation of this study is the potential loss to follow-up and missing datapoints due to several points of measurement.
- The results will be mainly based on self-reports, which are considered gold standard but can be subject to bias.

For peer review only

Introduction

Chronic postsurgical pain (CPSP) is defined as chronic pain that persists over time (at least 3 months) following a surgical procedure, and that has significant impact on physical and psychosocial quality of life (1,2). Meta-analytic estimates in children and adolescents describe that around 20% of pediatric patients undergoing major surgery report chronic pain three to twelve months after surgery (2). Highest pain intensity ratings are observed in two-weeks following surgery, with a steady decrease afterwards over the following six months (3), and pain ratings two weeks after surgery have been shown to predict the development of moderate to severe pain one year later (4,5). Further, most children and adolescents experience decreases in their physical activity levels following surgery, and it has been suggested that objectively monitored physical activity levels could be useful to indirectly assess functional recovery (6). A recent study in adolescents undergoing spinal fusion found that a majority of participants were characterized by a steep decrease of physical activity after surgery (7).

Previous research has identified psychosocial risk factors that are associated with the development of CPSP, among them presurgical pain intensity, child anxiety, child pain coping efficacy, child sleep patterns, and parental pain catastrophizing (2,8,9). Generally, emotions and emotion-related factors are known to influence pain, pain-related disability, and pain-related distress (10,11): There is some research on the role of emotion regulation in chronic pain (12), and the role of *negative* emotions is fairly well-documented in the pain experience (10,13,14). However, other emotion-related factors such as emotion differentiation and emotion variability have yet to be studied in the context of pediatric CPSP, as they present as good targets for prevention and treatment modules and can be screened for in clinical practice.

Emotion variability

Emotion variability describes the range of emotional fluctuations around an individual's average emotional intensity (15,16). Greater emotion variability has been linked to more mental health symptoms in adolescents (17,18), and higher emotion regulation demands (19). In pediatric chronic pain, one study found greater positive emotion variability to be associated with less pain-related interference and more engagement in activities despite pain (16). Emotion variability can be measured using the within-person standard deviation of repeated assessments of current emotional state (16,18,20,21).

Emotion differentiation

Emotion differentiation defines the level of specificity people use when identifying their emotional experiences (22–24). Higher emotion differentiation indicates greater ability to differentiate between one's own emotional states on a more fine-grained level and by using

For peer review only

precise terms (24). High or low emotion differentiation can be calculated by means of intraclass correlations, where high consistency in emotion ratings across a predefined episode (e.g., a week-long daily diary phase) suggests poorer differentiation (24,25). Previous research has demonstrated that higher emotion differentiation is associated with better mental health (see (26) for a meta-analysis). To date, emotion differentiation has not been studied in the context of pediatric chronic pain.

Emotion regulation

Emotion regulation refers to a person's ability to influence what emotions they have, when they have them, and how they experience and express these emotions (27,28). A handful of previous studies have explored the role of emotion regulation in pediatric chronic pain (e.g., (16,20,29,30), yielding mixed results: While some found that different types of emotion regulation, namely expressive suppression and cognitive reappraisal, were associated with more frequent hospitalization due to pain (29), and lower engagement in activities despite the pain (16), others did not identify significant associations between emotion regulation and pain intensity or pain-related disability (20,30). The habitual use of specific emotion regulation strategies is considered stable within individuals (31–33), and can be measured by means of questionnaires (19).

Familial influences on pediatric pain

Previous research has shown that around 50% of children and adolescents with chronic pain have a parent who has had chronic pain in the past or is currently living with it (34). Parents and the family environment have been identified as a "key context" (p. 702) regarding the understanding, assessment, and management of pediatric pain (35), and a bidirectional relationship between child functioning and parent factors is suggested (35–37). Regarding emotion-related factors, the family context is considered crucial: Children learn the appropriateness of valence, duration, and intensity of emotions and emotion expression by observing their parents and parents' emotion regulation behaviors (38,39).

In sum, previous studies have identified several risk factors for the development of CPSP, among them symptoms of anxiety, preoperative pain intensity, and poorer sleep quality as well as parental catastrophizing, even though the results were equivocal in part (5,6,40). The extent to which emotion-related factors can serve as *resilience* factors has had minimal investigation in the transition from acute to chronic postsurgical pain (5). Further, the promotion of resilience mechanisms as a prevention strategy has had limited focus in the context of pediatric chronic pain (7,41).

Objectives

For peer review only - http://bmjopen.bmj.com/site/about/guidelines.xhtml

The aim of this observational longitudinal study is to specifically examine the potential role of emotion-related factors (namely emotion variability, emotion differentiation, and emotion regulation), as well as familial influences in the development of CPSP and describe pain- and emotion-related trajectories following major surgery up to 12 months after surgery. We aim to identify whether and which emotion-related factors increase or decrease the risk for the development of CPSP after planned orthopedic surgery. To the best of our knowledge, this is the first study to specifically examine these emotion-related factors as both potential risk *and* resilience factors in the development of CPSP.

For peer review only

Methods and Analysis

Study design and setting

This is a prospective longitudinal observational study in patients undergoing scheduled orthopedic surgery and their parents. The study starts in January 2024 and will continue to run through the end of January 2027. As scheduled orthopedic surgeries are planned well in advance, patients can be approached early and asked about their interest in participating in the study. See Figure 1 for an overview of assessment time points. The study is registered at clinicaltrials.gov (NCT05816174). All participants will be recruited from three University Children's Hospitals in Switzerland.

Patient and Public Involvement

Patients were not involved in the design of the study, however, the first 10 families that complete the post assessment will be interviewed and asked about their experience in the study so far. This feedback will be used to adapt study procedures if needed. The results will be disseminated to study participants via a newsletter and a dissemination event where participants, clinical collaborators, and the interested public will be invited to discuss the study results.

Sample and Sample Size Calculation

The anticipated prevalence rate of CPSP is 25%, based on international estimates (2,42). The aim of this study is to recruit a sample of 280 child-parent dyads, resulting in an anticipated 70 children and adolescents with CPSP. In a power calculation for a logistic regression model with 10 events per variable, this allows to examine 7 predictor variables with CPSP status as the primary outcome. Assumptions regarding events per variable will be re-evaluated following recently proposed recommendations (43,44).

For peer review only - http://bmjopen.bmj.com/site/about/guidelines.xhtml

Eligibility criteria

We will include participants between 8 and 18 years who have a planned in-patient orthopedic surgery at one of the study centers. The child-parent dyads must be able to read (for younger children: with help) and understand German. Exclusion criteria will be serious comorbid health condition (e.g., cancer, severe neurological impairment, chronic illness requiring daily medication), and prior major surgery (e.g., prior spine surgery). Mental health comorbidities are no reason for exclusion.

Study Design

This is a prospective longitudinal observational study in patients undergoing scheduled orthopedic surgery and their parents. As scheduled orthopedic surgeries are planned well in advance, patients can be approached early and asked about their interest in participating in the study. See Figure 1 for an overview of assessment time points. The study is registered at clinicaltrials.gov (NCT05816174).

Procedures

Potential participants will be informed about the study by the clinical collaborators at each study site during one of their pre-surgical visits (e.g., anesthesia preparation interview, scheduling visit). Based on the clinical routine of the study centers, the procedures might differ slightly. If patients fall within the required age range, the clinical collaborators will provide the family with a leaflet with the most important study information. On the leaflet, a QR-Code brings families directly to a site on the website of the University Children's Hospital Zurich (main study center), where they can find the most important information again. If families express their initial interest about the study, the physician will take a note and inform the study team, which will then contact the family. A member of the study team will then approach the respective family at their next visit at the hospital or via phone call and go through the informed consent / assent procedure with them. In all cases, parents sign the consent form for their own participation. In children under the age of 14 years, parents also sign the consent form for their child's participation, while adolescents aged 14 years or more sign their own consent form. This is in line with the Human Research Act (45), that states that adolescents aged 14 years and more who are capable of judgement and who are to participate in a study with minimal risks can sign their own consent form. In participating hospitals, patients typically have (at least) two visits before the surgery. Once the written informed consent and assent is given, families will complete a battery of questionnaires as baseline measures before the surgery via direct link to the online questionnaires (see next section and Table 1 for an overview; see Supplement for an overview of reliability, validity, benefits, and

For peer review only - http://bmjopen.bmj.com/site/about/guidelines.xhtml

limitations of all questionnaires), using Research Electronic Data Capture (RedCap; (46)). In addition, participants will be asked to download the smartphone application SEMA 3 (47), a free tool for ecological momentary assessment provided by the University of Melbourne, Australia, especially developed for research needs.

Families will be called again two weeks after the surgery, and asked to complete the post-assessment via direct link to the questionnaires. Families will be contacted again 3 months (FU 1), 6 months (FU 2), and 12 months (FU 3) after surgery to complete the follow-up surveys, and asked to complete the surveys at each assessment point within one week after receiving the link to the survey; up to three weekly reminders will be sent after the initial message.

In addition to the questionnaires at each assessment point, all participants (i.e., children and parents) will complete daily diaries: To estimate the influence of emotion-related factors on pain trajectories, children and adolescents will complete two daily diary phases (i.e., two weeks of daily diary) during which they rate both their pain severity and emotional state (referred to as "pain and emotions" throughout the protocol; at baseline and post assessment), and one daily diary episode during which they only rate their pain intensity (referred to as "pain only" throughout the protocol; at Follow-up 1. See Figure 1). Parents will complete two daily diary phases (at baseline and post) during which they exclusively rate their emotional state.

<u>Daily diary - pain and emotions (two timepoints):</u> At baseline and post assessment, children and adolescents will complete ratings of their pain severity on an 11-point numeric rating scale with anchors 0 = "no pain" and 10 = "worst pain possible". Pain severity includes pain intensity, pain-related distress, and pain-related interference; hence, participants will give three different ratings (41,42). The pain ratings will be used to classify the presence of acute postsurgical pain at the post assessment (in combination with a health-related quality of life rating; see overview of questionnaires for this time point in Table 1). In addition, all participants will rate the extent to which they experience positive and negative emotions three times each day on their smartphone and during their normal daily routine over the course of 7 days. These ratings will be used to calculate a score of emotion differentiation (i.e., the ability to differentiate between emotional states), a mean value of positive and negative emotions, and an index of emotional variability (26,36,37; see below for more detailed description). The items of the short version of the Positive and Negative Affect Schedule for Children (PANAS-C) will be used to assess emotional state (38–40).

For peer review only - http://bmjopen.bmj.com/site/about/guidelines.xhtml

Daily diary - pain only (once): To diagnose CPSP, participants will be asked to rate their daily pain severity for 7 days starting at FU1. Participants will be prompted at the end of each day to respond on an 11-point numeric rating scale with anchors 0 = "no pain" and 10 = "worst pain possible". In addition, daily medication use will be indicated (Yes/No and listing medication in a free text field).

Number of steps taken each day will be counted as an indicator of changes in physical activity before and after surgery (i.e., at the post assessment), measured by means of accelerometer (GENEActive, Activeinsights Ltd), an ambulatory, non-invasive activity-monitoring device that registers physical activity, and can be worn around the wrist.

Study Measures

See Table 1 for an overview, see Supplement for an overview of reliability, validity, benefits, and limitations of all questionnaires.. For all measures, we use the validated German version (if available) or translate and back-translate the items with the help of a professional interpreter or native speaker. Measures of primary interest are marked with an * in Table 1, all other variables are considered exploratory.

Child Measures

Emotional state: The Positive and Negative Affect Schedule for Children (PANAS-C) (48–50) is a measures of positive and negative affect by providing mood adjectives (e.g. "scared", "happy"). Participants are asked to rate 10 items on a 5-point Likert-scale from 1 (= very slightly or not at all) to 5 (= very much) to assess the extent to which they currently feel each of 5 positively and 5 negatively valenced emotions.

Pain Screening Tool: The Pediatric Pain Screening Tool (PPST) (51,52) entails 9 self-reported items that belong to a physical and psychosocial subscale. Answers are given dichotomously (disagree = 0, agree = 1). Higher scores indicate higher risk status of poor pain-related outcomes.

Fear of Pain: The Fear of Pain Questionnaire for Children (FOPQ-C) (53) is a 24-item self-report inventory to assess fear of pain. Participants are asked to rate each item on a Likert-scale from 0 (= strongly disagree) to 4 (= strongly agree). A higher total score indicates higher fear.

For peer review only - http://bmjopen.bmj.com/site/about/guidelines.xhtml

Functional Disability: Children's self-reported difficulty in physical and psychosocial functioning due to their physical health is assessed by the Functional Disability Inventory (FDI) (54). 15 items are rated on a Likert-scale from 0 (= no problems) to 4 (= not possible) and concern perceptions of activity limitations during the past 2 weeks. Higher scores indicate greater disability.

Security in Parent-Child Relationships: The Security Scale (55) consists of 15 items and assesses participants' perceptions of security in parent-child relationships. Items are presented in a format of statements, participants choose which statement is more characteristic of them and then rate the statement on a Likert-scale from 1 (= really true) to 4 (= sort of true). Higher scores indicate a more secure attachment.

Symptoms of Anxiety and Depression: The RCADS-short version (56) is a 25-item self-report questionnaire used to assess symptoms of depression and anxiety. Participants are asked to indicate how often each item applies to them according to a 4-point Likert-scale from 0 (= never) to 3 (= always). High scores indicate high anxiety and depression symptoms.

Sleep: The Pittsburgh Sleep Quality Index (PSQI) (57) is the most commonly used retrospective self-report questionnaire that measures sleep quality over the previous month. We will use a version that has been slightly adapted for children by researchers of the University Children's Hospital. This version contains of 25 items, and each item is rated on a scale from 0 (= no difficulty) to 3 (= severe difficulty). In addition to a global sleep quality factor, seven domains of sleep difficulties can be assessed: Sleep quality, sleep latency, sleep duration, habitual sleep efficiency, sleep disturbances, use of sleeping medications, and daytime dysfunction. A higher global score indicates more sleep difficulties.

Sensory Processing Sensitivity: The Highly Sensitive Child Scale (HSC) (58) is a 12-item self-reported questionnaire. Participants are asked to rate each item on a Likert-scale from 1 (= not at all) to 7 (= extremely). In addition to a total score, three subscales can be calculated: Low Sensory Threshold (LST; 3 items, e.g., "I don't like watching TV programs that have a lot of violence in them"), Ease of Excitation (EOE; 5 items, e.g., "I get nervous when I have to do a lot in little time"), and Aesthetic Sensitivity (AES; 4 items, e.g., "I love nice smells"). Higher scores indicate higher sensitivity.

For peer review only

For peer review only - http://bmjopen.bmj.com/site/about/guidelines.xhtml

Emotion Regulation: The Emotion Regulation Questionnaire for Children and Adolescents (ERQ-CA) (19) is a self-report questionnaire containing 10 items. Items are rated on a Likert-scale from 1 (= strongly disagree) to 7 (= strongly agree), and assess individual differences in the habitual use of two emotion regulation strategies, namely Cognitive Reappraisal (e.g., “When I’m faced with a stressful situation, I make myself think about it in a way that helps me stay calm”) and Expressive Suppression (e.g., “I keep my emotions to myself”). Higher scores indicate more habitual use of the respective emotion regulation strategy.

Quality of Life: The Pediatric Quality of Life Inventory (PedsQL) (59) is a self-or parent-reported questionnaire for children and adolescents. 23 Items are rated on a Likert-scale from 0 (= never a problem) to 4 (= almost always a problem). The questionnaire consists of the subscales physical functioning (e.g., “It is hard for me to run”), emotional functioning (e.g., “I feel sad or blue”), social functioning (e.g., “It is hard to keep up with my peers”), and school functioning (e.g., “It is hard to pay attention in class”) in the past month. Higher scores indicate better quality of life.

For peer review only

Social Support: The Social Support Questionnaire for Children (SSQC) (60) consists of 50 items. It measures children’s social support across five distinct sources of support: parents, relatives, nonrelative adults, siblings, and peers. Items are rated on a 4-point Likert-scale ranging from 0 (= never or rarely true) to 3 (= often or always true). Higher scores indicate higher levels of perceived support.

Symptoms of Posttraumatic Stress: The Child and Adolescent Trauma Screen 2 (CATS-2) (61) contains a checklist of 15 potentially traumatic events (PTEs), followed by an item asking which of the PTEs bothers them most. Posttraumatic stress symptoms are measured by 20 items that are rated on a 4-point Likert-scale ranging from 0 (= never) to 3 (= almost always). Five yes/no items assess whether the previously rated posttraumatic stress symptoms interfere with five key areas of functioning and are used to assess psychosocial functioning.

Parent Measures

Postoperative Pain: With the Postoperative Pain Measure for Parents (PPMP) (62), parents can assess and estimate their child's postoperative pain. The checklist includes 15 items where the parents select “yes” (= 1 point) or “no” (= 0 points) as to whether the child exhibits pain-related behavior, with higher scores indicating more postoperative pain.

The Postoperative Pain Measure for Parents (PPMP) [illegible]

Fear of Pain Questionnaire: The Parent Fear of Pain Questionnaire (FOPQ-P) (35) is a 23-item self-report inventory to reflect a parent's own fear associated with their child's pain experience. Parents are asked to rate each item on a Likert-scale from 0 (= strongly disagree) to 4 (= strongly agree). A higher total score indicates higher fear of pain.

Symptoms of Anxiety and Depression: The Hospital Anxiety and Depression Scale (HADS) (63) is a widely used measure of psychological distress, and indicates the presence of symptoms of depression (seven items) and anxiety (seven items) over a 1-week period. The 14 items are rated on a four-point Likert-scale ranging from 0 to 3, with higher scores indicate greater symptom severity.

Sensory Processing Sensitivity: The Highly Sensitive Person Scale (HSP) (64,65) is a 12-item self-reported questionnaire, and participants are asked to rate each item on a Likert-scale from 1 (= strongly disagree) to 7 (= strongly agree). In addition to a total score, three subscales can be calculated, as for the HSC Scale: Low Sensory Threshold, Ease of Excitation, and Aesthetic Sensitivity. Higher scores reflect higher sensitivity.

Emotion Regulation: The Emotion Regulation Questionnaire (ERQ) (31) assesses the habitual use of two emotion regulation strategies, namely Cognitive Reappraisal (6 items) and Expressive Suppression (4 items). The 10 self-reported items are rated on a 7-point Likert-scale ranging from 1 (= strongly disagree) to 7 (= strongly agree). Higher scores indicate higher usage of the respective strategy.

Well-Being: Parental well-being will be measured using he WHO-5 index (66). It consists of 5 items that are rated on a 6-point Likert-scale ranging from 0 (= never) to 5 (=always), indicating how often participants have felt in "good spirits", "active", "relaxed", etc. over the course of the last 2 weeks. Higher scores indicate more well-being.

Table 1. Measures, Tools, Domain and Time-Points for Data Collection

**Domain**		**Measure**	**Completed by**	**BL**	**Post**	**FU 1**	**FU 2**	**FU 3**
**Demographics**	Age, gender, school, school attendance and performance; family	Parent and self-report: online baseline questionnaire	Parent(s) and child	X				

For peer review only - http://bmjopen.bmj.com/site/about/guidelines.xhtml

For peer review only

	constellation, parental educational level and work status							
**Medical history**	Neonatal intensive care (yes/no), previous surgeries (yes/no; which one), child's pain history (pain duration, pain location)	Parent report: online baseline questionnaire	Parents	X				
	Complications in the hospital (yes/no) Pain treatment immediately following surgery	Child's medication list in electronic health record	Data entered by study team		X			
**Experience-sampling method**	Positive and negative emotions (emotion variability and emotion differentiation)*	Items from the Positive and Negative Affect Schedule (48)	Daily diary: child and parent	X	X			
	Pain severity	Numeric Rating Scale (NRS), 0-10	Daily diary: child	X	X	X		
**Psychosocial factors child**								
	Pain severity (intensity, distress, interference (67))*	Numeric Rating Scale (NRS), 0-10	Child self-report				X	X
	Preoperative screening	Pediatric Pain Screening Tool (51,52)	Child self-report	X				
	Functional Disability	Functional Disability Index (FDI; (54))	Child self-report	X	X	X	X	X
	Fear of Pain	Fear of Pain Questionnaire (53)	Child self-report	X				
	Pain catastrophizing	Pain Catastrophizing scale – 3 item version (68)	Child self-report	X				
	Attachment; parent–child relationship	Security Scale (69)	Child self-report	X				
	Symptoms of anxiety and depression*	Revised Child Anxiety and Depression Scale-short version (56)	Child self-report	X	X	X	X	X
	Sleep	Pittsburgh Sleep Quality Index (PSQI; (57))	Child self-report	X	X	X	X	X
	Sensory processing sensitivity	Highly Sensitive Child Scale (58)	Child self-report	X				
	Emotion regulation*	Emotion Regulation Questionnaire – Child and Adolescent version (19)	Child self-report	X				
	Quality of life	Pediatric Quality of Life Inventory (59)	Child self-report	X	X	X	X	X
	Social support	Social Support Questionnaire for Children (60)	Child self-report	X				

	Posttraumatic stress symptoms	Child and Adolescent Trauma Screening (CATS) (70)	Child self-report	X		X		
**Psychosocial factors parents**								
	Parents' own pain history	2 questions: "(1) Have you ever and (2) are you currently living with persistent or recurrent pain for at least 3 months?"	Parent self-report	X				
	Postoperative pain measure (child's pain)	Postoperative Pain Measure for Parents (62)	Parent report		X			
	Fear of pain	Parent Fear of Pain Questionnaire (35)	Parent self-report	X				
	Pain catastrophizing	Pain Catastrophizing Scale – 3 item version (68)	Parent self-report	X				
	Anxiety and depression*	Anxiety and Depression Scale (63)	Parent self-report	X	X	X	X	X
	Sensory processing sensitivity	Highly Sensitive Person Scale (65)	Parent self-report	X				
	Emotion regulation*	Emotion Regulation Questionnaire (31)	Parent self-report	X				
	Quality of life	World Health Organization Well-Being Index (66)	Parent self-report	X	X	X	X	X
**Primary outcome**								
	Chronic postsurgical pain	Presence of CPSP: pain diary for 7 consecutive days (NRS) + health-related quality of life (PedsQL, see above)				X		

Note. Primary predictors are marked with an *. Unmarked variables are considered secondary or exploratory.

Primary Outcome

The primary endpoint is CPSP at FU 1. CPSP is defined as greater than minimal pain (pain intensity ≥ 3) on more than 50% of days as measured by daily diary over 7 days, and impairment in health-related quality of life (score of ≤ 74.9 on the *Pediatric Quality of Life Inventory*, PedsQL (59), i.e., 1 SD below the population mean based on normative US data). The combination of pain and quality of life measures will result in a binary variable (CPSP yes/no). This measurement of CPSP is supported by previous research (6,71,72), and a modified definition of CPSP by the International Association for the Study of Pain (IASP) (73).

For peer review only

Secondary Outcomes

Secondary endpoints are trajectories of pain and emotion-related factors over time (i.e., baseline through FU3), number of steps taken by patients, pain severity, and quality of life. Postoperative complications will be monitored using the modified Clavien-Dindo Classification (74).

Statistical methods

Descriptive statistics will include mean and standard deviation or median and inter quartile range for normally distributed and non-normally distributed or ordinal variables measured at baseline. Categorical variables will be reported as number and percentage of total. The primary outcome is binary, therefore generalized linear regression models or generalized linear mixed effects models will be used, using a logit link function. Fixed effects will be estimated for the primary predictors (as marked in Table 1). Random effects will be included in the longitudinal model to account for repeated observations in the individual participants. The resulting estimands will be odds ratios and 95% confidence intervals. Variables at baseline will be used in the prediction model, with performance measures area under the curve (AUC), calibration, and scaled Brier Score (75,76). Variables measured consecutively over multiple days at each assessment interval will be averaged over time. We will use the rule of 10 events per variable (EPV) that would determine the number of independent variables in the regression model. Potentially time-varying covariates will be addressed. Potentially non-linear relationships of the independent variables with outcome will be evaluated. Further, the purpose of the multivariable regression analysis is to develop a risk score for CPSP. This will allow to identify variables that increase or decrease the risk for CPSP, by combining them into a multivariable model.

The pre-selected variables (i.e., the primary predictors) will be assessed for collinearity with pair-wise scatter plots, pair-wise interaction terms will be included successively into the model. For the pre-selected variables, no variable selection will be performed. In an exploratory analysis, potential other variables will be evaluated and the process will be described in more detail in the statistical analysis plan.

Any missing values in the independent variables or the outcome will be addressed with 100-fold multiple imputation, using chained equations. Resulting estimates will be pooled using Rubin's rule (77,78).

We will conduct bivariate analyses to compare study completers versus study dropouts to detect potential differences regarding participant demographic between the groups.

For peer review only - http://bmjopen.bmj.com/site/about/guidelines.xhtml

Baseline daily pain ratings will be averaged for each participant to calculate a mean baseline pain intensity score. Post assessment and FU 1 daily pain ratings will be combined with health-related quality of life data to define binary outcome variables for acute postsurgical pain and CPSP. Acute postsurgical pain and CPSP will be defined independently.

For the trajectory analysis, the classification method K-means for longitudinal data will be used to determine clusters in the data (R package kml) (79). The analysis of clusters joint trajectories (R package kml3d) (79) will be applied, as this analysis allows summarizing several correlated continuous variables into a single nominal variable (80). The trajectory analyses will be considered exploratory. Alternative clustering methods would be based on the risk score and participants with high risk for developing CPSP can be identified based on a cut-off of 10% predicted probability.

A detailed statistical analysis plan will be written up and finalized before data export for analysis. All analyses will be performed with the R programming language in a fully scripted way and dynamic reporting will be used to guarantee highest level of reproducibility.

Monitoring

The study will be monitored for quality and regulatory adherence. The monitor, who is not involved in our study and supervised by the Clinical Trials Center of the University Hospital Zurich, Switzerland, will ensure that the specifications of the project plan and the associated processes are adhered to.

Ethics and Dissemination

The Ethics Committee of the Canton of Zurich, Switzerland, has approved the study and all study-related procedures including the consent procedures for minors under the age of 18 years (ID: 2023-01475). All participants will be compensated for their time by means of a voucher. We will reimburse participating families per assessment time point, so that families who drop out will still receive a certain amount of compensation. The first few participating families will be asked about general accessibility of the study. Their feedback will be implemented in study procedures if necessary.

The risk of adverse effects due to the study are considered to be minimal. All project data will be handled with uttermost discretion and will only be accessible to the study team. Data will be stored on a secured and password-protected server.

For peer review only

This study is registered at clinicaltrials.gov (identification number: NCT05816174). After study completion, the study team organizes a dissemination workshop aiming at youth with CPSP and their parents, researchers, clinicians, and the interested public, to discuss study results, clinical implications, and formulate future research questions.

For peer review only

Full references

1. Schug SA, Lavand'homme P, Barke A, Korwisi B, Rief W, Treede RD, et al. The IASP classification of chronic pain for ICD-11: chronic postsurgical or posttraumatic pain. Pain. 2019 Jan;160(1):45–52.
2. Rabbitts JA, Fisher E, Rosenbloom BN, Palermo TM. Prevalence and predictors of chronic postsurgical pain in children: A systematic review and meta-analysis. J Pain. 2017 Jun;18(6):605–14.
3. Connelly M, Fulmer RD, Prohaska J, Anson L, Dryer L, Thomas V, et al. Predictors of Postoperative Pain Trajectories in Adolescent Idiopathic Scoliosis. Spine. 2014 Feb 1;39(3):E174.
4. Pagé MG, Stinson J, Campbell F, Isaac L, Katz J. Identification of pain-related psychological risk factors for the development and maintenance of pediatric chronic postsurgical pain. J Pain Res. 2013 Mar 5;6:167–80.
5. Rosenbloom BN, Katz J. Modeling the transition from acute to chronic postsurgical pain in youth: A narrative review of epidemiologic, perioperative, and psychosocial factors. Canadian Journal of Pain. 2022 Jun 15;6(2):166–74.
6. Rabbitts JA, Zhou C, Groenewald CB, Durkin L, Palermo TM. Trajectories of postsurgical pain in children: risk factors and impact of late pain recovery on long-term health outcomes after major surgery. PAIN. 2015 Nov;156(11):2383–9.
7. Beeckman M, Hughes S, Van der Kaap-Deeder J, Plasschaert F, Michielsen J, Moens P, et al. Risk and Resilience Predictors of Recovery after Spinal Fusion Surgery in Adolescents. The Clinical Journal of Pain [Internet]. 2021 Aug 23 [cited 2021 Sep 8];Publish Ahead of Print. Available from: https://journals.lww.com/10.1097/AJP.0000000000000971
8. Katz J, Weinrib AZ, Clarke H. Chronic postsurgical pain: From risk factor identification to multidisciplinary management at the Toronto General Hospital Transitional Pain Service. Canadian Journal of Pain. 2019 Jul 15;3(2):49–58.
9. Rabbitts JA, Groenewald CB, Tai GG, Palermo TM. Presurgical psychosocial predictors of acute postsurgical pain and quality of life in children undergoing major surgery. J Pain. 2015 Mar;16(3):226–34.
10. Wiech K, Tracey I. The influence of negative emotions on pain: behavioral effects and neural mechanisms. Neuroimage. 2009 Sep;47(3):987–94.
11. Barke A, Koechlin H, Korwisi B, Locher C. Emotional distress: Specifying a neglected part of chronic pain. European Journal of Pain. 2020;24(3):477–80.
12. Koechlin H, Coakley R, Schechter N, Werner C, Kossowsky J. The role of emotion regulation in chronic pain: A systematic literature review. Journal of Psychosomatic Research. 2018 Apr 1;107:38–45.
13. Berna C, Leknes S, Holmes EA, Edwards RR, Goodwin GM, Tracey I. Induction of depressed mood disrupts emotion regulation neurocircuitry and enhances pain unpleasantness. Biological psychiatry. 2010 Jun 1;67(11):1083–90.
14. Janssen SA. Negative affect and sensitization to pain. Scand J Psychol. 2002 Apr;43(2):131–7.
15. Reitsema AM, Jeronimus BF, Dijk M van, Jonge P de. Emotion dynamics in children and adolescents: A meta-analytic and descriptive review. Emotion [Internet]. 2021 Jan [cited 2021 Apr 19]; Available from: https://research.rug.nl/en/publications/emotion-dynamics-in-children-and-adolescents-a-meta-analytic-and-
16. Koechlin H, Beeckman M, Meier AH, Locher C, Goubert L, Kossowsky J, et al. Association of parental and adolescent emotion-related factors with adolescent chronic pain behaviors. PAIN [Internet]. 2022 [cited 2021 Oct 28]; Available from: https://journals.lww.com/pain/Abstract/9000/Association_of_parental_and_adolescent.97860.aspx
17. Neumann A, van Lier PAC, Frijns T, Meeus W, Koot HM. Emotional Dynamics in the

For peer review only - http://bmjopen.bmj.com/site/about/guidelines.xhtml

Development of Early Adolescent Psychopathology: A One-Year Longitudinal Study. J Abnorm Child Psychol. 2011 Jul 1;39(5):657–69.
18. Silk JS, Steinberg L, Morris AS. Adolescents' Emotion Regulation in Daily Life: Links to Depressive Symptoms and Problem Behavior. Child Development. 2003;74(6):1869–80.
19. Gullone E, Taffe J. The Emotion Regulation Questionnaire for Children and Adolescents (ERQ-CA): a psychometric evaluation. Psychol Assess. 2012 Jun;24(2):409–17.
20. Connelly M, Bromberg MH, Anthony KK, Gil KM, Franks L, Schanberg LE. Emotion Regulation Predicts Pain and Functioning in Children With Juvenile Idiopathic Arthritis: An Electronic Diary Study. J Pediatr Psychol. 2012 Jan 1;37(1):43–52.
21. Kalokerinos EK, Murphy SC, Koval P, Bailen NH, Crombez G, Hollenstein T, et al. Neuroticism may not reflect emotional variability. Proceedings of the National Academy of Sciences. 2020 Apr 28;117(17):9270–6.
22. Barrett LF, Gross J, Christensen TC, Benvenuto M. Knowing what you're feeling and knowing what to do about it: Mapping the relation between emotion differentiation and emotion regulation. Cognition and Emotion. 2001 Nov 1;15(6):713–24.
23. Kashdan TB, Barrett LF, McKnight PE. Unpacking Emotion Differentiation: Transforming Unpleasant Experience by Perceiving Distinctions in Negativity. Curr Dir Psychol Sci. 2015 Feb 1;24(1):10–6.
24. Nook E. Emotion Differentiation and Youth Mental Health: Current Understanding and Open Questions [Internet]. PsyArXiv; 2021 [cited 2021 Apr 26]. Available from: https://psyarxiv.com/qkdbu/
25. Kalokerinos EK, Erbas Y, Ceulemans E, Kuppens P. Differentiate to Regulate: Low Negative Emotion Differentiation Is Associated With Ineffective Use but Not Selection of Emotion-Regulation Strategies. Psychol Sci. 2019 Jun;30(6):863–79.
26. Seah THS, Coifman KG. Emotion differentiation and behavioral dysregulation in clinical and nonclinical samples: A meta-analysis. Emotion. 2021;No Pagination Specified-No Pagination Specified.
27. Gross JJ. Emotion Regulation. In: Handbook of emotions. 3rd ed. New York: The Guilford Press; 2008.
28. Gross JJ. Emotion Regulation: Current Status and Future Prospects. Psychological Inquiry. 2015 Jan 2;26(1):1–26.
29. Tsao JC, Jacob E, Seidman LC, Lewis MA, Zeltzer LK. Psychological aspects and hospitalization for pain crises in youth with sickle-cell disease. J Health Psychol. 2014 Mar;19(3):407–16.
30. Koechlin H, Donado C, Locher C, Kossowsky J, Lionetti F, Pluess M. Sensory processing sensitivity in adolescents reporting chronic pain: an exploratory study. Pain Rep. 2023 Jan 6;8(1):e1053.
31. Gross JJ, John OP. Individual differences in two emotion regulation processes: implications for affect, relationships, and well-being. J Pers Soc Psychol. 2003 Aug;85(2):348–62.
32. English T, Lee IA, John OP, Gross JJ. Emotion regulation strategy selection in daily life: The role of social context and goals. Motiv Emot. 2017 Apr;41(2):230–42.
33. Brans K, Koval P, Verduyn P, Lim YL, Kuppens P. The regulation of negative and positive affect in daily life. Emotion. 2013 Oct;13(5):926–39.
34. Poppert Cordts KM, Stone AL, Beveridge JK, Wilson AC, Noel M. The (Parental) Whole Is Greater Than the Sum of Its Parts: A Multifactorial Model of Parent Factors in Pediatric Chronic Pain. J Pain. 2019 Jul;20(7):786–95.
35. Simons LE, Smith A, Kaczynski K, Basch M. Living in fear of your child's pain: the Parent Fear of Pain Questionnaire. Pain. 2015 Apr;156(4):694–702.
36. Chambers CT, Craig KD, Bennett SM. The Impact of Maternal Behavior on

For peer review only - http://bmjopen.bmj.com/site/about/guidelines.xhtml

Children's Pain Experiences: An Experimental Analysis. J Pediatr Psychol. 2002 Apr 1;27(3):293–301.
37. Palermo TM, Chambers CT. Parent and family factors in pediatric chronic pain and disability: An integrative approach. PAIN. 2005 Dec;119(1–3):1–4.
38. Bariola E, Gullone E, Hughes EK. Child and adolescent emotion regulation: the role of parental emotion regulation and expression. Clin Child Fam Psychol Rev. 2011 Jun;14(2):198–212.
39. Morris AS, Silk JS, Steinberg L, Myers SS, Robinson LR. The Role of the Family Context in the Development of Emotion Regulation. Soc Dev. 2007 May 1;16(2):361–88.
40. Ellyson AM, Gordon G, Zhou C, Rabbitts JA. Trajectories, Risk Factors, and Impact of Persistent Pain After Major Musculoskeletal Surgery in Adolescents: A Replication Study. The Journal of Pain [Internet]. 2021 Dec 31 [cited 2022 Jun 2]; Available from: https://www.sciencedirect.com/science/article/pii/S1526590021003916
41. Goubert L, Trompetter H. Towards a science and practice of resilience in the face of pain. European Journal of Pain. 2017;21(8):1301–15.
42. Williams G, Howard RF, Liossi C. Persistent postsurgical pain in children and young people: prediction, prevention, and management. Pain Rep. 2017 Aug 21;2(5):e616.
43. Christodoulou E, van Smeden M, Edlinger M, Timmerman D, Wanitschek M, Steyerberg EW, et al. Adaptive sample size determination for the development of clinical prediction models. Diagnostic and Prognostic Research. 2021 Mar 22;5(1):6.
44. Riley RD, Ensor J, Snell KIE, Harrell FE, Martin GP, Reitsma JB, et al. Calculating the sample size required for developing a clinical prediction model. BMJ. 2020 Mar 18;368:m441.
45. Confederation TFA of the S. Federal Act on Research involving Human Beings: Human Research Act (HRA). 801.30 Sep 1, 2013.
46. Harris PA, Taylor R, Thielke R, Payne J, Gonzalez N, Conde JG. Research electronic data capture (REDCap) - A metadata-driven methodology and workflow process for providing translational research informatics support. Journal of Biomedical Informatics. 2009;42(2):377–81.
47. Koval P, Hinton J, Dozo N, Gleeson J, Alvarez M, Harrison A, et al. SEMA3: Smartphone Ecological Momentary Assessment. 2019.
48. Watson D, Clark LA, Tellegen A. Development and validation of brief measures of positive and negative affect: The PANAS scales. Journal of Personality and Social Psychology. 1988;54(6):1063–70.
49. Laurent J, Catanzaro SJ, Joiner Jr. TE, Rudolph KD, Potter KI, Lambert S, et al. A measure of positive and negative affect for children: Scale development and preliminary validation. Psychological Assessment. 1999;11(3):326–38.
50. Wilson K, Gullone E, Moss S. The Youth Version of the Positive and Negative Affect Schedule: A Psychometric Validation. Behaviour Change. 1998 Sep;15(3):187–93.
51. Simons LE, Smith A, Ibagon C, Coakley R, Logan DE, Schechter N, et al. Pediatric Pain Screening Tool (PPST): Rapid identification of risk in youth with pain complaints. Pain. 2015 Aug;156(8):1511–8.
52. Narayanasamy S, Yang F, Ding L, Geisler K, Glynn S, Ganesh A, et al. Pediatric Pain Screening Tool: A Simple 9-Item Questionnaire Predicts Functional and Chronic Postsurgical Pain Outcomes After Major Musculoskeletal Surgeries. The Journal of Pain [Internet]. 2021 Jul 17 [cited 2021 Sep 24]; Available from: https://www.sciencedirect.com/science/article/pii/S1526590021002911
53. Flack F, Gerlach AL, Simons LE, Zernikow B, Hechler T. Validation of the German fear of pain questionnaire in a sample of children with mixed chronic pain conditions. Eur J Pain. 2017;21(7):1224–33.
54. Offenbächer M, Kohls N, Walker L, Hermann C, Hügle B, Jäger N, et al. Functional

For peer review only

limitations in children and adolescents suffering from chronic pain: validation and psychometric properties of the German Functional Disability Inventory (FDI-G). Rheumatol Int. 2016 Oct 1;36(10):1439–48.
55. Kerns KA, Klepac L, Cole A. Security Scale. Developmental Psychology [Internet]. 1996 [cited 2023 Apr 13]; Available from: https://psycnet.apa.org/doiLanding?doi=10.1037%2Ft17560-000
56. Ebesutani C, Reise SP, Chorpita BF, Ale C, Regan J, Young J, et al. The Revised Child Anxiety and Depression Scale-Short Version: Scale reduction via exploratory bifactor modeling of the broad anxiety factor. Psychological Assessment. 2012;24(4):833–45.
57. Buysse DJ, Reynolds CFI, Monk TH, Berman SR, Kupfer DJ. The Pittsburgh Sleep Quality Index: A new instrument for psychiatric practice and research. Psychiatry Research. 1988;28:193–213.
58. Pluess M, Assary E, Lionetti F, Lester KJ, Krapohl E, Aron EN, et al. Environmental sensitivity in children: Development of the Highly Sensitive Child Scale and identification of sensitivity groups. Dev Psychol. 2018;54(1):51–70.
59. Varni JW, Seid M, Rode CA. The PedsQL™: Measurement Model for the Pediatric Quality of Life Inventory. Medical Care. 1999 Feb;37(2):126.
60. Gordon-Hollingsworth AT, Thompson JE, Geary MA, Schexnaildre MA, Lai BS, Kelley ML. Social Support Questionnaire for Children. Measurement and Evaluation in Counseling and Development. 2016 Apr 1;49(2):122–44.
61. Sachser C, Berliner L, Risch E, Rosner R, Birkeland MS, Eilers R, et al. The child and Adolescent Trauma Screen 2 (CATS-2) – validation of an instrument to measure DSM-5 and ICD-11 PTSD and complex PTSD in children and adolescents. European Journal of Psychotraumatology. 2022 Dec 19;13(2):2105580.
62. Chambers CT, Reid GJ, McGrath PJ, Finley GA. Development and preliminary validation of a postoperative pain measure for parents 11This paper was presented at the meeting Pediatric Pain: Challenges, Innovations, and Costs in Atlanta, GA, November 4, 1995 (Voted Best Abstract). Pain. 1996 Dec 1;68(2):307–13.
63. Snaith RP. The Hospital Anxiety And Depression Scale. Health and Quality of Life Outcomes. 2003 Aug 1;1(1):29.
64. Aron EN, Aron A. Sensory-processing sensitivity and its relation to introversion and emotionality. J Pers Soc Psychol. 1997 Aug;73(2):345–68.
65. Pluess M. Sensory-Processing Sensitivity: A potential mechanism of differential susceptibility. In: Biennial Meeting of the Society for Research in Child Development (SRCD), Seattle, WA. 2013.
66. Bech P, Olsen LR, Kjoller M, Rasmussen NK. Measuring well-being rather than the absence of distress symptoms: a comparison of the SF-36 Mental Health subscale and the WHO-Five well-being scale. International Journal of Methods in Psychiatric Research. 2003;12(2):85–91.
67. Treede RD, Rief W, Barke A, Aziz Q, Bennett MI, Benoliel R, et al. Chronic pain as a symptom or a disease: the IASP Classification of Chronic Pain for the International Classification of Diseases (ICD-11). Pain. 2019 Jan;160(1):19–27.
68. Durand H, Birnie KA, Noel M, Vervoort T, Goubert L, Boerner KE, et al. State Versus Trait: Validating State Assessment of Child and Parental Catastrophic Thinking About Children's Acute Pain. The Journal of Pain. 2017 Apr 1;18(4):385–95.
69. Kerns KA, Klepac L, Cole A. Peer relationships and preadolescents' perceptions of security in the child-mother relationship. Developmental Psychology. 1996 May;32(3):457–66.
70. Sachser C, Berliner L, Holt T, Jensen TK, Jungbluth N, Risch E, et al. International development and psychometric properties of the Child and Adolescent Trauma Screen (CATS). J Affect Disord. 2017 Mar 1;210:189–95.

For peer review only - http://bmjopen.bmj.com/site/about/guidelines.xhtml

71. Rabbitts JA, Palermo TM, Zhou C, Meyyappan A, Chen L. Psychosocial Predictors of Acute and Chronic Pain in Adolescents Undergoing Major Musculoskeletal Surgery. The Journal of Pain. 2020 Nov 1;21(11):1236–46.
72. Rabbitts JA, Palermo TM, Zhou C, Mangione-Smith R. Pain and Health-Related Quality of Life After Pediatric Inpatient Surgery. The Journal of Pain. 2015 Dec 1;16(12):1334–41.
73. Werner MU, Kongsgaard UE. I. Defining persistent post-surgical pain: is an update required? Br J Anaesth. 2014 Jul;113(1):1–4.
74. Camino Willhuber G, Slullitel P, Taype Zamboni D, Albergo J, Terrasa S, Piuzzi N, et al. Validation of a modified Clavien-Dindo Classification for postoperative complications in orthopedic surgery. Rev Fac Cien Med Univ Nac Cordoba. 2020 Aug 21;77(3):161–7.
75. Steyerberg EW, Vickers AJ, Cook NR, Gerds T, Gonen M, Obuchowski N, et al. Assessing the performance of prediction models: a framework for some traditional and novel measures. Epidemiology. 2010 Jan;21(1):128–38.
76. Gerds TA, Cai T, Schumacher M. The performance of risk prediction models. Biom J. 2008 Aug;50(4):457–79.
77. Rubin DB. Inference and missing data. Biometrika. 1976 Dec 1;63(3):581–92.
78. Little RJA, Rubin DB. Statistical Analysis with Missing Data. John Wiley & Sons; 2019. 465 p.
79. Genolini C, Alacoque X, Sentenac M, Arnaud C. kml and kml3d: R Packages to Cluster Longitudinal Data. Journal of Statistical Software. 2015 Jun 1;65(1):1–34.
80. Donado C, Friedrich Y, Kossowsky J, Locher C, Koechlin H. Exposure to Parental Depressive Symptoms: A Longitudinal Analysis on the Association with Adolescents' Depressive Symptoms and Adjustment Problems. J Dev Behav Pediatr. 2020 Sep;41(7):522–33.

For peer review only

For peer review only - http://bmjopen.bmj.com/site/about/guidelines.xhtml

Authors' contributions

HK has designed the study, obtained funding, wrote the first draft of the manuscript, and acts as project leader and sponsor for the study described in this protocol.

JH has collaborated closely with HK in the process of detailing the study plan, has written and revised the manuscript, and is the PhD student in this project.

CCH, TD, and SC are the clinical collaborators of this study. They have all provided substantial input in the research plan and the study procedures, and have critically revised the manuscript.

JR has provided substantial input and feedback on the research plan, choice of outcome variables, and study procedures, and has critically revised the manuscript.

CL has provided substantial feedback on the design of the study and all study related procedures, and has critically revised the manuscript.

UH is responsible for data analysis in this project, has written the data analysis plan, provided advice on the power calculation, and has critically revised the manuscript.

All authors approved the final version of the manuscript, and agree to be accountable for all aspects of the work in ensuring that questions related to the accuracy or integrity of any part of the work are appropriately investigated and resolved.

Funding statement

This work is supported by a Swiss National Science Foundation grant awarded to HK: PZ00P1_208850 (Title: "Understanding and preventing chronic postsurgical pain in children")

JR is supported by National Institutes of Health National Institutes of Arthritis, Musculoskeletal, and Skin Diseases under award number by K24AR080786 (PI: J. Rabbitts). The content is solely the responsibility of the authors and does not necessarily represent the official views of the National Institutes of Health.

Competing interest statement

The authors report no conflict of interest.

Figure Captions.

Figure 1. Overview of Assessment Time Points

For peer review only

Figure 1. Overview of Assessment Time Points

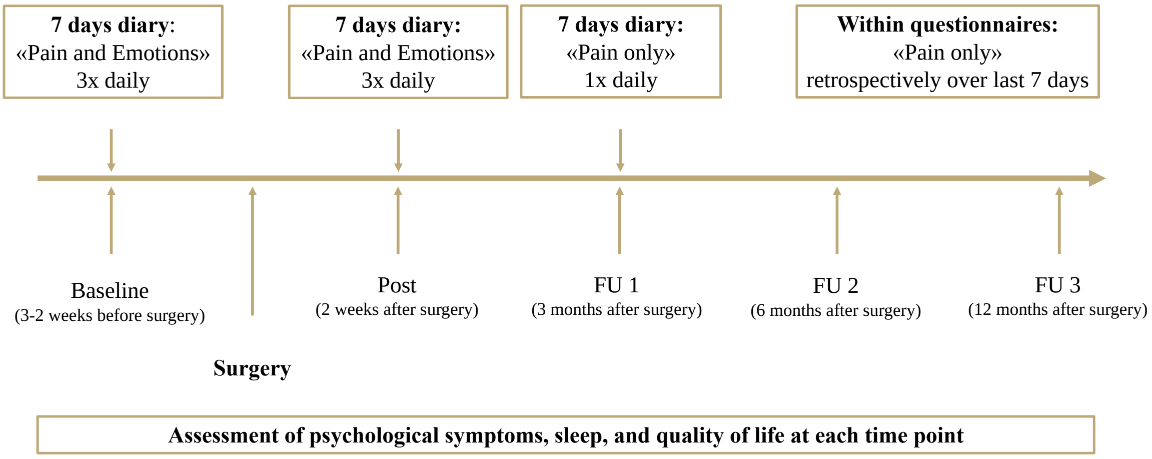

peer review only

Supplement: Overview of reliability, validity, benefits, and limitations of questionnaires used in the study.

Domain		Measure	Reliability	Validity	Benefits	Limitations
**Experience-sampling method**	Positive and negative emotions*	Items from the Positive and Negative Affect Schedule (1)	Cronbach's Alpha high, .86 for PA and .87 for NA scale (1).	Excellent factorial validity (convergent correlation high: .89 to .95; discriminant correlation low: -.02 to -.18); items good markers of corresponding factors (1).	External validity demonstrated by correlation with other scales. Stability over 2-month time period (1).	Heterogeneity the factorial structure of the PANAS scores (2).
	Pain severity	Numeric Rating Scale (NRS), 0-10				
**Psychosocial factors child**						
	Pain severity (intensity, distress, inter-ference)*	Numeric Rating Scale (NRS), 0-10			Individual scores of pain severity can be converted into the ICD-11 categories of none (0), mild (1), moderate (2) or severe (3) and merged into one extension code endorsed by the WHO (3,4).	More field testing is needed to further validate NRS scales in pediatric populations (5).
	Pre-operative screening	Pediatric Pain Screening Tool (6,7)	Overall PPST: adequate to excellent discrimination; psychosocial subscale AUC	Sensitivity and specificity for the PPST ranged from adequate to excellent, with regard to significant disability (78%, 68%) and high	Defines risk groups to inform efficient treatment decision-making; valid screening tool that allows early	Generalizability of results needs further studies in surgeries with different pathology and requires further

		adequate to acceptable (6). Total score (ICC = 0.75) and psychosocial subscale (ICC = 0.70) acceptable 2-week test-retest reliability (6).	emotional distress (81%, 63%) (6).	identification of patients presenting with impairing pain-related comorbidities (6).	stratification by race and surgical/pain characteristics, and interventions (7). German translation not yet validated.
Functional Disability	Functional Disability Index (FDI; (8))	Good internal reliability of the total FDI in the German cohort (Cronbach's α = .9) similar to the original US version (8).	Good construct validity with significant correlations between the FDI and different variables (e.g. Childhood Health Assessment Questionnaire [total score], pain-related interference in everyday life, affective distress) (8).	Tested in a large cohort of German children and adolescents. Data confirm that the German version of the FDI retains the characteristics of the original FDI and is a reliable and valid instrument (8).	Majority of females subjects; inclusion of patients from a single specialized center restrict the generalizability of the results (8).
Fear of Pain*	Fear of Pain Questionnaire (9)	Good internal consistency of the total scale Cronbach's α = 0.87. Internal consistency also good for both subscales (Fear subscale: α = 0.89; avoidance subscale: α = 0.76) (9)	The two-factor structure of the GFOPQ-C shows that fear of pain includes two key dimensions: Fear of Pain and Avoidance of Activities (9).	Accurate translation of the English version conducted on the basis of the forward–backward translation. Instrument applied in a mixed pain sample (9).	To evaluate the degree of increased fear of pain in pain samples, it would be beneficial a comparison to healthy controls (9)

Pain catastrophizing *	Pain Catastrophizing Scale – 3 item version (10)	Chronbach's Alpha high α = .84 and .79 for PCS-C state (10)	Significant relationships with child pain-related outcomes (ie, child pain intensity and child state anxiety), no issue with multicollinearity (10)	Short (3-item scale) can be efficiently used (= high correlation with 6-item scale). Large, diverse sample of children (10)	Due to small number of items per factor, additional research needed to support the 1-factor model. (10)
Attachment parent–child relationship *	Security Scale (11)	Cronbach's α = .74 for both mothers and fathers of sixth graders; and α = .75 and α = .52 for mothers and fathers of third graders respectively (11)	Moderate stability and meaningful associations with other attachment measures, caregiver sensitivity, and indexes of child's adjustment (1).	One of the most widely used measures of attachment in middle childhood and early adolescence. Some associations of the scale with child's outcomes hold regardless of the child's sex and age (12).	Many cross-sectional rather than longitudinal studies (12).
Symptoms of anxiety and depression*	Revised Child Anxiety and Depression Scale-short version (13).	15-item Anxiety Total scale evidenced significant correspondence with anxiety diagnostic groups,10-item Depression Total scale associated with acceptable reliability (Cronbach's α =	Based on a cross-sample validation strategy, values fell in the "acceptable" classification category (AUC=.74, SE=.03) (13).	Potentially useful for repeated measurement in clinical settings as well as wide-scale screenings. Assesses both anxiety and depressive symptoms (13).	Additional test development efforts needed to ensure that the shortened RCADS meets the clinical demands of reduced test length, while maintaining adequate reliability of its broad scale scores (13).

		.80 and .79, respectively) (13).			
Sleep*	Pittsburgh Sleep Quality Index (PSQI; (14))	High internal consistency (Cronbach's α = 0.83) (14)	Sensitive and specific measure: Global PSQI score > 5, sensitivity of 89.6% and specificity of 86.5% (kappa = 0.75, p < 0.001) (14)	Provides a standardized, quantitative measure of sleep quality that quickly identifies good and poor sleepers (14)	Adult population sample (14)
Sensory processing sensitivity	Highly Sensitive Child Scale (15)	HSC-12-items: acceptable internal consistency with Cronbach's α = .71 and .74. The subscales showed lower internal consistency with α = .73/.69 (15).	Divergent validity was established (15).	12-item HSC scale is a psychometrically robust measure that performs well in both children and adolescents. Studies with large sample, replication of results, previous use in samples with chronic pain (15,16).	All data are based on self-report. Most data were provided by children and adolescents residing in the United Kingdom (15).
Emotion regulation*	Emotion Regulation Questionnaire – Child and Adolescent version (17,18)	Sound internal consistency: cognitive reappraisal subscale Cronbach's α = .82, expressive suppression subscale α = .79 (17).	Good factor structure, convergent validity supported (17).	Tested on participants aged between 10 and 18 years (17), previously used in samples with chronic pain (16,19).	Measures only two emotion regulation strategies, cognitive reappraisal and expressive suppression (20).
Quality of life	Pediatric Quality of Life Inventory (21)	High internal consistency (Cronbach's α =	Across scales, item-level internal consistency supported content	Cronbach α good in various samples, including	Issue of detecting small changes in quality of life only

			0.83 for self-report and α = 0.86 for parent-report). α for self-report ranged from .70 to .89 (19).	validity. Item-level discriminant validity tests showed more variability (21)	adolescent samples with chronic pain (16).	partially addressed, more longitudinal data could help to better understand this aspect (21).
	Social support*	Social Support Questionnaire for Children (22)	High internal consistency. Cronbach's α for total scale and five subscales ranging from .89 to .97 (23).	Good factorial and construct validity.Convergent validity was partially supported (23).	The SSQC identifies important sources of social support in varying samples (e.g. for particular ethnic populations the extended family and community member (23).	Substantially longer than a widely used measure of social support in children (23).
	Posttraumatic stress symptoms	Child and Adolescent Trauma Screening (CATS) (24).	Good to excellent reliability with Cronbach's α ranging between .88 and .94 for all three language samples (US, Germany, Norway) (25).	Convergent discriminant validity: medium to strong correlations with measures of depression (r =.62–.82) and anxiety (r =.40–.77). Low to medium correlations with externalizing symptoms (r =−.15–.43) (25).	Different countries involved in the validation (USA, Germany, and Norway) (25).	Pending external validation with a DSM-5 based semi-structured clinical interview (25).
**Psychosocial factors parents**						
	Parents' own pain history*	2 questions: "(1) Have you ever and (2) are you currently				

	living with persistent or recurrent pain for at least 3 months?”				
Postoperative pain measure (child’s pain)	Postoperative Pain Measure for Parents (26)	Cronbach’s α = .88 and .87 on postoperative days 1 and 2, respectively (26).	Excellent sensitivity (>80%) and specificity (>80%) in children who reported clinically significant levels of pain (26).	The minimal training required for parents to use this tool supports its utility as a clinical tool (26).	The items were developed based on parent reports and thus could miss important behavior changes in children (26).
Fear of pain	Parent Fear of Pain Questionnaire (27)	The 21-item scale showed a Cronbach's α = .91(27).	Solid construct validity, supported for the subscales (27).	Excellent fit for a complex model, after a minor modification to include parent avoidance as an indicator of parent behavior (27).	Tested in samples of predominantly mothers (92%), Caucasian (90%) and female children (74.8%) (27).
Pain cata-strophizing *	Pain Cata-strophizing Scale – 3 item version (10)	Chronbach’s Alpha high α = .79 and .73 for PCS-P state (10).	Significant relationships with parent pain-related outcomes (i.e., parental distress), no issue with multicollinearity (10).	Short (3-item scale) can be efficiently used (= high correlation with 6-item scale). Large, diverse sample of parents (10).	Due to small number of items per factor, additional research needed to support the 1-factor model (10).
Anxiety and depression*	Anxiety and Depression Scale (28)	Cronbach's alpha high α = .78 for the depression scale and α = .81 for the anxiety scale (29).	Original model (28) demonstrates the best fit. Good discriminant validity in large study and across various populations and countries (29).	Simple and reliable tool for medical practice. In addition to validation for use in the elderly the HADS has been validated for use in adolescents (28).	Few studies validated the questionnaire with a general population or included a control group taken from

					the general population (29).
Sensory processing sensitivity	Highly Sensitive Person Scale (30,31)	Reliability of 27-item version Cronbach's α = .87 and .85 in 2 different studies (31).	The 27-item version showed good content validity and the measure's discriminant, convergent (31). Study confirmed that the HSP scale reflects a unitary dimension of environmental sensitivity and identified in addition three sensitivity groups in the general population (high, low and medium sensitivity) (32).	Diversity of samples as well as qualitative and quantitative methods used to develop scale and support conlcusions (31).	Parental impact on sensitive individuals not explored in depth (31).
Emotion regulation*	Emotion Regulation Questionnaire (33)	Cronbach's alpha satisfactory α = .74 for cognitive reappraisal and .76 for expressive suppression (34).	Indices of CFA were satisfactory for the German sample; support for measurement invariance of the ERQ (35).	Good conformity of the translation with the original (34).	Mainly tested in student samples (36).
Quality of life	World Health Organization Well-Being Index (37)	Internal consistency of the WHO-Five and the Mental Health subscale acceptable (Cronbach's α = 0.84. and 0.8) (37).	Adequate validity both as a screening tool for depression and as an outcome measure in clinical trials (38).	Includes subscales measuring both physical health and mental health. Compare a scale containing a mixture of distress and well being items (37). Validated in both younger and	Has been researched predominantly in the field of depression (38).

		elderly persons (38).
**Primary outcome**		
Chronic postsurgical pain	Presence of CPSP: pain diary for 7 consecutive days (NRS) + health-related quality of life (PedsQL)	

Note. AUC = area under the curve , ICC = intraclass correlation coefficient, PCS-C = Pain Catastrophizing Scale for Children, PCS-P = Pain Catastrophizing Scale for Parents.

For peer review only

References

1. Watson D, Clark LA, Tellegen A. Development and validation of brief measures of positive and negative affect: The PANAS scales. J Pers Soc Psychol. 1988;54(6):1063–70.
2. Flores-Kanter PE, Garrido LE, Moretti LS, Medrano LA. A modern network approach to revisiting the Positive and Negative Affective Schedule (PANAS) construct validity. J Clin Psychol. 2021;77(10):2370–404.
3. Barke A, Koechlin H, Korwisi B, Locher C. Emotional distress: Specifying a neglected part of chronic pain. Eur J Pain. 2020;24(3):477–80.
4. Treede RD, Rief W, Barke A, Aziz Q, Bennett MI, Benoliel R, et al. Chronic pain as a symptom or a disease: the IASP Classification of Chronic Pain for the International Classification of Diseases (ICD-11). PAIN. 2019 Jan;160(1):19.
5. McGrath PJ, Walco GA, Turk DC, Dworkin RH, Brown MT, Davidson K, et al. Core Outcome Domains and Measures for Pediatric Acute and Chronic/Recurrent Pain Clinical Trials: PedIMMPACT Recommendations. J Pain. 2008 Sep 1;9(9):771–83.
6. Simons LE, Smith A, Ibagon C, Coakley R, Logan DE, Schechter N, et al. Pediatric Pain Screening Tool (PPST): Rapid identification of risk in youth with pain complaints. Pain. 2015 Aug;156(8):1511–8.
7. Narayanasamy S, Yang F, Ding L, Geisler K, Glynn S, Ganesh A, et al. Pediatric Pain Screening Tool: A Simple 9-Item Questionnaire Predicts Functional and Chronic Postsurgical Pain Outcomes After Major Musculoskeletal Surgeries. J Pain [Internet]. 2021 Jul 17 [cited 2021 Sep 24]; Available from: https://www.sciencedirect.com/science/article/pii/S1526590021002911
8. Offenbächer M, Kohls N, Walker L, Hermann C, Hügle B, Jäger N, et al. Functional limitations in children and adolescents suffering from chronic pain: validation and psychometric properties of the German Functional Disability Inventory (FDI-G). Rheumatol Int. 2016 Oct 1;36(10):1439–48.
9. Flack F, Gerlach AL, Simons LE, Zernikow B, Hechler T. Validation of the German fear of pain questionnaire in a sample of children with mixed chronic pain conditions. Eur J Pain Lond Engl. 2017;21(7):1224–33.
10. Durand H, Birnie KA, Noel M, Vervoort T, Goubert L, Boerner KE, et al. State Versus Trait: Validating State Assessment of Child and Parental Catastrophic Thinking About Children's Acute Pain. J Pain. 2017 Apr 1;18(4):385–95.
11. Kerns KA, Klepac L, Cole A. Peer relationships and preadolescents' perceptions of security in the child-mother relationship. Dev Psychol. 1996 May;32(3):457–66.
12. Brumariu LE, Madigan S, Giuseppone KR, Movahed Abtahi M, Kerns KA. The Security Scale as a measure of attachment: Meta-analytic evidence of validity. Attach Hum Dev. 2018;20(6):600–25.
13. Ebesutani C, Reise SP, Chorpita BF, Ale C, Regan J, Young J, et al. The Revised Child Anxiety and Depression Scale-Short Version: Scale reduction via exploratory bifactor modeling of the broad anxiety factor. Psychol Assess. 2012;24(4):833–45.
14. Buysse DJ, Reynolds CFI, Monk TH, Berman SR, Kupfer DJ. The Pittsburgh Sleep Quality Index: A new instrument for psychiatric practice and research. Psychiatry Res. 1988;28:193–213.
15. Pluess M, Assary E, Lionetti F, Lester KJ, Krapohl E, Aron EN, et al. Environmental sensitivity in children: Development of the Highly Sensitive Child Scale and identification of sensitivity groups. Dev Psychol. 2018;54(1):51–70.

For peer review only

16. Koechlin H, Donado C, Locher C, Kossowsky J, Lionetti F, Pluess M. Sensory processing sensitivity in adolescents reporting chronic pain: an exploratory study. Pain Rep. 2023 Jan 6;8(1):e1053.
17. Gullone E, Taffe J. The Emotion Regulation Questionnaire for Children and Adolescents (ERQ-CA): a psychometric evaluation. Psychol Assess. 2012 Jun;24(2):409–17.
18. Abler B, Kessler H. Emotion Regulation Questionnaire – Eine deutschsprachige Fassung des ERQ von Gross und John. Diagnostica. 2009 Jul 1;55(3):144–52.
19. Koechlin H, Beeckman M, Meier AH, Locher C, Goubert L, Kossowsky J, et al. Association of parental and adolescent emotion-related factors with adolescent chronic pain behaviors. PAIN [Internet]. 2022 [cited 2021 Oct 28]; Available from: https://journals.lww.com/pain/Abstract/9000/Association_of_parental_and_adolescent.97860.aspx
20. Dorn C, Spindler G, Kullik A, Petermann F, Barnow S. Erfassung von Emotionsregulationsstrategien–eine Übersicht. Psychol Rundsch. 2013 Oct;64(4):217–27.
21. Varni JW, Seid M, Rode CA. The PedsQL™: Measurement Model for the Pediatric Quality of Life Inventory. Med Care. 1999 Feb;37(2):126.
22. Gordon-Hollingsworth AT, Thompson JE, Geary MA, Schexnaildre MA, Lai BS, Kelley ML. Social Support Questionnaire for Children. Meas Eval Couns Dev. 2016 Apr 1;49(2):122–44.
23. Gordon-Hollingsworth AT, Thompson JE, Geary MA, Schexnaildre MA, Lai BS, Kelley ML. Social Support Questionnaire for Children: Development and Initial Validation. Meas Eval Couns Dev. 2016 Apr;49(2):122–44.
24. Sachser C, Berliner L, Risch E, Rosner R, Birkeland MS, Eilers R, et al. The child and Adolescent Trauma Screen 2 (CATS-2) – validation of an instrument to measure DSM-5 and ICD-11 PTSD and complex PTSD in children and adolescents. Eur J Psychotraumatology. 2022 Dec 19;13(2):2105580.
25. Sachser C, Berliner L, Holt T, Jensen TK, Jungbluth N, Risch E, et al. International development and psychometric properties of the Child and Adolescent Trauma Screen (CATS). J Affect Disord. 2017 Mar 1;210:189–95.
26. Chambers CT, Reid GJ, McGrath PJ, Finley GA. Development and preliminary validation of a postoperative pain measure for parents 11This paper was presented at the meeting Pediatric Pain: Challenges, Innovations, and Costs in Atlanta, GA, November 4, 1995 (Voted Best Abstract). Pain. 1996 Dec 1;68(2):307–13.
27. Simons LE, Smith A, Kaczynski K, Basch M. Living in fear of your child's pain: the Parent Fear of Pain Questionnaire. Pain. 2015 Apr;156(4):694–702.
28. Snaith RP. The Hospital Anxiety And Depression Scale. Health Qual Life Outcomes. 2003 Aug 1;1(1):29.
29. Bocéréan C, Dupret E. A validation study of the Hospital Anxiety and Depression Scale (HADS) in a large sample of French employees. BMC Psychiatry. 2014;14(1):1–11.
30. Pluess M. Sensory-Processing Sensitivity: A potential mechanism of differential susceptibility. In: Biennial Meeting of the Society for Research in Child Development (SRCD), Seattle, WA. 2013.

For peer review only

31. Aron EN, Aron A. Sensory-processing sensitivity and its relation to introversion and emotionality. J Pers Soc Psychol. 1997 Aug;73(2):345–68.
32. Lionetti F, Aron A, Aron EN, Burns GL, Jagiellowicz J, Pluess M. Dandelions, tulips and orchids: Evidence for the existence of low-sensitive, medium-sensitive and high-sensitive individuals. Transl Psychiatry. 2018;8(1):24.
33. Gross JJ, John OP. Individual differences in two emotion regulation processes: implications for affect, relationships, and well-being. J Pers Soc Psychol. 2003 Aug;85(2):348–62.
34. Abler B, Kessler H. Emotion regulation questionnaire–Eine deutschsprachige Fassung des ERQ von Gross und John. Diagnostica. 2009;55(3):144–52.
35. Sala MN, Molina P, Abler B, Kessler H, Vanbrabant L, van de Schoot R. Measurement invariance of the Emotion Regulation Questionnaire (ERQ). A cross-national validity study. Eur J Dev Psychol. 2012;9(6):751–7.
36. Preece DA, Becerra R, Robinson K, Gross JJ. The emotion regulation questionnaire: psychometric properties in general community samples. J Pers Assess. 2019;
37. Bech P, Olsen LR, Kjoller M, Rasmussen NK. Measuring well-being rather than the absence of distress symptoms: a comparison of the SF-36 Mental Health subscale and the WHO-Five well-being scale. Int J Methods Psychiatr Res. 2003;12(2):85–91.
38. Topp CW, Østergaard SD, Søndergaard S, Bech P. The WHO-5 Well-Being Index: a systematic review of the literature. Psychother Psychosom. 2015;84(3):167–76.

For peer review only
